# Supplementary material for: Deletion of wfs1 Impairs Oligodendrocyte Precursor Cells Dorsal Distribution and Myelination Through the wfs1-hmgcs1 Axis in Zebrafish
Source: Biology (Basel). 2026 Aug 16;15(16):1402. doi: 10.3390/biology15161402 (PMC13510151; doi:10.3390/biology15161402)
Supplement: Supplementary file 1 [file biology-15-01402-s001.zip › File S1-Transcriptome sequencing data and differentially expressed genes (DEGs) in wfs1-- zebrafish at 5 dpf/2.KEGG/wfs1KOvsWT/GSEA/wfs1KO/METABOLIC_PATHWAYS/METABOLIC_PATHWAYS(DRE01100).html]

Details for gene set METABOLIC\_PATHWAYS(DRE01100)[GSEA]

|  || Dataset | deg.sample |
| Phenotype | sample.cls |
| Upregulated in class | wfs1KO |
| GeneSet | METABOLIC\_PATHWAYS(DRE01100) |
| Enrichment Score (ES) | 0.2473075 |
| Normalized Enrichment Score (NES) | 1.3369148 |
| Nominal p-value | 0.0 |
| FDR q-value | 0.25320122 |
| FWER p-Value | 0.931 |
Table: GSEA Results Summary

  

Fig 1: Enrichment plot: METABOLIC\_PATHWAYS(DRE01100)      
 Profile of the Running ES Score & Positions of GeneSet Members on the Rank Ordered List

  

| PROBE | DESCRIPTION (from dataset) | GENE SYMBOL | GENE\_TITLE | RANK IN GENE LIST | RANK METRIC SCORE | RUNNING ES | CORE ENRICHMENT || 1 | 724010\_1 | dgkaa |  |  | 12 | 4.962 | 0.0030 | Yes |
| 2 | 573619 | ndufab1a |  |  | 14 | 4.959 | 0.0064 | Yes |
| 3 | 541329 | gcsha |  |  | 17 | 4.955 | 0.0098 | Yes |
| 4 | 337236\_2 | synj1 |  |  | 33 | 4.929 | 0.0127 | Yes |
| 5 | 447801\_1 | aldh8a1 |  |  | 45 | 4.909 | 0.0158 | Yes |
| 6 | 560753\_1 | tpi1b |  |  | 49 | 4.890 | 0.0191 | Yes |
| 7 | 406370 | pla2g6 |  |  | 54 | 4.870 | 0.0223 | Yes |
| 8 | 447914\_1 | cox17 |  |  | 57 | 4.862 | 0.0256 | Yes |
| 9 | 406449\_1 | ptges3a |  |  | 59 | 4.859 | 0.0289 | Yes |
| 10 | 406762\_1 | pgd |  |  | 66 | 4.835 | 0.0321 | Yes |
| 11 | 100330864 | LOC100330864 |  |  | 70 | 4.818 | 0.0353 | Yes |
| 12 | 678558\_1 | mtmr7a |  |  | 123 | 4.623 | 0.0368 | Yes |
| 13 | 116517 | adh5 |  |  | 148 | 4.502 | 0.0391 | Yes |
| 14 | 555317 | si:ch211-276a23.5 |  |  | 156 | 4.461 | 0.0420 | Yes |
| 15 | 368717 | itpka |  |  | 192 | 4.239 | 0.0437 | Yes |
| 16 | 335715 | mdh1ab |  |  | 215 | 4.103 | 0.0458 | Yes |
| 17 | 565235\_1 | aldh5a1 |  |  | 226 | 4.030 | 0.0483 | Yes |
| 18 | 560827\_1 | prps1b |  |  | 229 | 4.021 | 0.0510 | Yes |
| 19 | 394103 | sardh |  |  | 267 | 3.820 | 0.0524 | Yes |
| 20 | 359840 | atp6v1b2 |  |  | 274 | 3.761 | 0.0548 | Yes |
| 21 | 100002875 | hmox2a |  |  | 277 | 3.753 | 0.0574 | Yes |
| 22 | 436725\_1 | acp5b |  |  | 287 | 3.706 | 0.0596 | Yes |
| 23 | 100002015\_1 | cds1 |  |  | 298 | 3.655 | 0.0618 | Yes |
| 24 | 437019\_1 | pcca |  |  | 307 | 3.602 | 0.0640 | Yes |
| 25 | 559054\_1 | hmgcra |  |  | 326 | 3.550 | 0.0659 | Yes |
| 26 | 324510\_1 | gcnt4a |  |  | 360 | 3.443 | 0.0672 | Yes |
| 27 | 403070\_1 | rgn |  |  | 363 | 3.424 | 0.0695 | Yes |
| 28 | 447881 | gbgt1l3 |  |  | 366 | 3.401 | 0.0718 | Yes |
| 29 | 393592 | cyp2p9 |  |  | 370 | 3.378 | 0.0740 | Yes |
| 30 | 100333665 | gbe1a |  |  | 374 | 3.364 | 0.0763 | Yes |
| 31 | 337236 | synj1 |  |  | 397 | 3.305 | 0.0778 | Yes |
| 32 | 562840 | rdh10a |  |  | 403 | 3.287 | 0.0799 | Yes |
| 33 | 561471\_1 | pigl |  |  | 423 | 3.219 | 0.0815 | Yes |
| 34 | 317744\_1 | dnmt3bb.1 |  |  | 424 | 3.201 | 0.0837 | Yes |
| 35 | 565117 | cel.2 |  |  | 426 | 3.195 | 0.0859 | Yes |
| 36 | 553523\_1 | trit1 |  |  | 449 | 3.128 | 0.0873 | Yes |
| 37 | 334195\_1 | mat1a |  |  | 452 | 3.119 | 0.0894 | Yes |
| 38 | 406473 | pck2 |  |  | 487 | 3.031 | 0.0904 | Yes |
| 39 | 561416\_1 | pfkpb |  |  | 497 | 2.998 | 0.0922 | Yes |
| 40 | 557675\_1 | c1galt1a |  |  | 517 | 2.933 | 0.0936 | Yes |
| 41 | 492517 | zgc:103586 |  |  | 528 | 2.910 | 0.0953 | Yes |
| 42 | 553639\_1 | gmpr |  |  | 557 | 2.830 | 0.0963 | Yes |
| 43 | 103911233 | LOC103911233 |  |  | 562 | 2.827 | 0.0981 | Yes |
| 44 | 445287 | hpdb |  |  | 629 | 2.726 | 0.0978 | Yes |
| 45 | 30505 | dbh |  |  | 690 | 2.615 | 0.0976 | Yes |
| 46 | 406702 | pdha1a |  |  | 691 | 2.614 | 0.0994 | Yes |
| 47 | 406539 | amy2a |  |  | 699 | 2.591 | 0.1009 | Yes |
| 48 | 570579\_1 | g6pd |  |  | 730 | 2.516 | 0.1017 | Yes |
| 49 | 447942 | cox6a2 |  |  | 746 | 2.477 | 0.1029 | Yes |
| 50 | 406575\_1 | lta4h |  |  | 766 | 2.441 | 0.1039 | Yes |
| 51 | 560225 | cept1a |  |  | 775 | 2.429 | 0.1054 | Yes |
| 52 | 321892\_1 | ckmt1 |  |  | 776 | 2.428 | 0.1070 | Yes |
| 53 | 100003615 | prdm2a |  |  | 778 | 2.426 | 0.1087 | Yes |
| 54 | 100006223 | si:dkey-266f7.9 |  |  | 781 | 2.419 | 0.1103 | Yes |
| 55 | 445818 | cthl |  |  | 799 | 2.387 | 0.1114 | Yes |
| 56 | 450083 | gla |  |  | 814 | 2.368 | 0.1125 | Yes |
| 57 | 394130\_1 | acad8 |  |  | 829 | 2.343 | 0.1137 | Yes |
| 58 | 445083 | dhrs3a |  |  | 831 | 2.340 | 0.1153 | Yes |
| 59 | 450084\_1 | gss |  |  | 836 | 2.334 | 0.1168 | Yes |
| 60 | 447815 | rimkla |  |  | 841 | 2.325 | 0.1183 | Yes |
| 61 | 541429 | aoc2 |  |  | 862 | 2.303 | 0.1192 | Yes |
| 62 | 431773\_1 | enoph1 |  |  | 869 | 2.294 | 0.1206 | Yes |
| 63 | 317746 | suclg2 |  |  | 871 | 2.285 | 0.1221 | Yes |
| 64 | 100006589\_1 | idh1 |  |  | 876 | 2.275 | 0.1236 | Yes |
| 65 | 393799 | pycr1a |  |  | 890 | 2.250 | 0.1247 | Yes |
| 66 | 791448\_1 | qdprb1 |  |  | 911 | 2.212 | 0.1255 | Yes |
| 67 | 436909 | khk |  |  | 947 | 2.172 | 0.1259 | Yes |
| 68 | 797311 | LOC797311 |  |  | 976 | 2.119 | 0.1264 | Yes |
| 69 | 445049 | zgc:92137 |  |  | 984 | 2.113 | 0.1276 | Yes |
| 70 | 556077\_1 | plod3 |  |  | 1001 | 2.091 | 0.1285 | Yes |
| 71 | 103910413\_1 | tymp |  |  | 1012 | 2.083 | 0.1297 | Yes |
| 72 | 393444 | pygmb |  |  | 1013 | 2.082 | 0.1311 | Yes |
| 73 | 393441 | hacd3 |  |  | 1017 | 2.075 | 0.1324 | Yes |
| 74 | 559020 | ptgs2b |  |  | 1021 | 2.074 | 0.1338 | Yes |
| 75 | 103911241 | LOC103911241 |  |  | 1022 | 2.074 | 0.1352 | Yes |
| 76 | 334431\_1 | adsl |  |  | 1025 | 2.072 | 0.1366 | Yes |
| 77 | 557552 | zgc:153031 |  |  | 1026 | 2.071 | 0.1380 | Yes |
| 78 | 555344 | si:dkey-202e17.1 |  |  | 1028 | 2.068 | 0.1394 | Yes |
| 79 | 322481 | cel.1 |  |  | 1043 | 2.053 | 0.1404 | Yes |
| 80 | 492735 | si:ch211-212o1.2 |  |  | 1044 | 2.053 | 0.1418 | Yes |
| 81 | 558466\_1 | uckl1b |  |  | 1053 | 2.045 | 0.1429 | Yes |
| 82 | 30659\_1 | dnmt3bb.2 |  |  | 1058 | 2.040 | 0.1442 | Yes |
| 83 | 100005375 | si:ch211-135f11.4 |  |  | 1072 | 2.023 | 0.1452 | Yes |
| 84 | 550560\_1 | entpd6 |  |  | 1083 | 2.011 | 0.1462 | Yes |
| 85 | 406662\_2 | msmo1 |  |  | 1111 | 1.984 | 0.1467 | Yes |
| 86 | 100005763 | uck2a |  |  | 1117 | 1.979 | 0.1479 | Yes |
| 87 | 393860 | gcdha |  |  | 1119 | 1.976 | 0.1493 | Yes |
| 88 | 561410\_1 | tat |  |  | 1121 | 1.974 | 0.1506 | Yes |
| 89 | 571967 | ido1 |  |  | 1123 | 1.972 | 0.1519 | Yes |
| 90 | 767750\_2 | atp5ib |  |  | 1131 | 1.967 | 0.1531 | Yes |
| 91 | 100330410 | alp3 |  |  | 1139 | 1.962 | 0.1542 | Yes |
| 92 | 560649 | si:ch211-214p16.3 |  |  | 1157 | 1.949 | 0.1550 | Yes |
| 93 | 100004700 | cyp24a1 |  |  | 1170 | 1.931 | 0.1559 | Yes |
| 94 | 393542\_1 | selenbp1 |  |  | 1193 | 1.905 | 0.1565 | Yes |
| 95 | 394230 | st3gal5 |  |  | 1201 | 1.894 | 0.1576 | Yes |
| 96 | 565743\_1 | pld2 |  |  | 1216 | 1.885 | 0.1584 | Yes |
| 97 | 405800 | dao.2 |  |  | 1217 | 1.883 | 0.1597 | Yes |
| 98 | 568726 | adcy7 |  |  | 1220 | 1.881 | 0.1609 | Yes |
| 99 | 368239 | aldh2.2 |  |  | 1221 | 1.881 | 0.1622 | Yes |
| 100 | 569989\_1 | ca5a |  |  | 1225 | 1.874 | 0.1634 | Yes |
| 101 | 140425 | gc2 |  |  | 1232 | 1.865 | 0.1645 | Yes |
| 102 | 101886891 | LOC101886891 |  |  | 1249 | 1.852 | 0.1653 | Yes |
| 103 | 550579\_1 | idh3g |  |  | 1275 | 1.830 | 0.1657 | Yes |
| 104 | 402953\_1 | pnp6 |  |  | 1279 | 1.829 | 0.1669 | Yes |
| 105 | 799099\_1 | lpin1 |  |  | 1367 | 1.749 | 0.1652 | Yes |
| 106 | 100003383 | pmvk |  |  | 1388 | 1.734 | 0.1657 | Yes |
| 107 | 555401 | aoc1 |  |  | 1405 | 1.721 | 0.1663 | Yes |
| 108 | 333997\_1 | ampd3b |  |  | 1407 | 1.721 | 0.1675 | Yes |
| 109 | 393744\_1 | dctpp1 |  |  | 1425 | 1.706 | 0.1681 | Yes |
| 110 | 445326\_1 | akr1a1a |  |  | 1428 | 1.705 | 0.1692 | Yes |
| 111 | 560387 | c17h14orf159 |  |  | 1431 | 1.704 | 0.1703 | Yes |
| 112 | 394142 | hpda |  |  | 1435 | 1.701 | 0.1714 | Yes |
| 113 | 553969 | cyp3a65 |  |  | 1441 | 1.696 | 0.1724 | Yes |
| 114 | 393493 | gstt2 |  |  | 1479 | 1.665 | 0.1723 | Yes |
| 115 | 81882 | dhfr |  |  | 1508 | 1.649 | 0.1725 | Yes |
| 116 | 103911886 | si:dkey-93h22.4 |  |  | 1514 | 1.645 | 0.1735 | Yes |
| 117 | 100151036\_1 | ipmka |  |  | 1520 | 1.644 | 0.1745 | Yes |
| 118 | 574009 | alpi.2 |  |  | 1560 | 1.616 | 0.1743 | Yes |
| 119 | 337131 | c1galt1b |  |  | 1564 | 1.614 | 0.1753 | Yes |
| 120 | 550266 | acer1 |  |  | 1565 | 1.614 | 0.1764 | Yes |
| 121 | 140615 | fads2 |  |  | 1590 | 1.600 | 0.1767 | Yes |
| 122 | 406789 | pank1a |  |  | 1591 | 1.599 | 0.1778 | Yes |
| 123 | 571738 | pla2g1b |  |  | 1592 | 1.599 | 0.1789 | Yes |
| 124 | 553401 | hadhaa |  |  | 1608 | 1.593 | 0.1795 | Yes |
| 125 | 571356 | pik3c2a |  |  | 1634 | 1.580 | 0.1798 | Yes |
| 126 | 572841 | mboat1 |  |  | 1662 | 1.561 | 0.1800 | Yes |
| 127 | 393113 | upp2 |  |  | 1678 | 1.557 | 0.1805 | Yes |
| 128 | 393580 | bco1l |  |  | 1682 | 1.555 | 0.1815 | Yes |
| 129 | 431727 | mgat4b |  |  | 1713 | 1.529 | 0.1816 | Yes |
| 130 | 767680 | zgc:153760 |  |  | 1748 | 1.506 | 0.1815 | Yes |
| 131 | 393764 | ndufb2 |  |  | 1769 | 1.481 | 0.1818 | Yes |
| 132 | 777632 | mccc1 |  |  | 1800 | 1.466 | 0.1818 | Yes |
| 133 | 564201\_1 | ca7 |  |  | 1805 | 1.463 | 0.1827 | Yes |
| 134 | 560486 | xdh |  |  | 1812 | 1.458 | 0.1835 | Yes |
| 135 | 447920 | aldh3a2b |  |  | 1830 | 1.444 | 0.1840 | Yes |
| 136 | 493602 | asah2 |  |  | 1865 | 1.427 | 0.1838 | Yes |
| 137 | 567720 | idua |  |  | 1901 | 1.412 | 0.1836 | Yes |
| 138 | 561117\_1 | atp6v0a2b |  |  | 1905 | 1.412 | 0.1845 | Yes |
| 139 | 58068 | pcxb |  |  | 1914 | 1.406 | 0.1852 | Yes |
| 140 | 559198 | mtmr7b |  |  | 1919 | 1.402 | 0.1860 | Yes |
| 141 | 324134\_1 | asmtl |  |  | 1924 | 1.399 | 0.1869 | Yes |
| 142 | 322506 | nqo1 |  |  | 1931 | 1.396 | 0.1876 | Yes |
| 143 | 110438097 | LOC110438097 |  |  | 1946 | 1.392 | 0.1881 | Yes |
| 144 | 445129\_1 | sdhc |  |  | 1953 | 1.389 | 0.1889 | Yes |
| 145 | 402826\_1 | si:ch73-71d17.1 |  |  | 1957 | 1.386 | 0.1897 | Yes |
| 146 | 100526683 | aldh1l1 |  |  | 1969 | 1.380 | 0.1903 | Yes |
| 147 | 559403\_1 | acaca |  |  | 1982 | 1.374 | 0.1909 | Yes |
| 148 | 431762\_1 | mgst1.2 |  |  | 1991 | 1.372 | 0.1916 | Yes |
| 149 | 751785 | aldh1a3 |  |  | 2030 | 1.355 | 0.1912 | Yes |
| 150 | 560807 | adcy6b |  |  | 2040 | 1.353 | 0.1919 | Yes |
| 151 | 100384896 | ugt1a6 |  |  | 2054 | 1.346 | 0.1924 | Yes |
| 152 | 100331714 | fut8b |  |  | 2093 | 1.328 | 0.1920 | Yes |
| 153 | 100384897 | ugt1a5 |  |  | 2102 | 1.324 | 0.1926 | Yes |
| 154 | 554145 | elovl8b |  |  | 2103 | 1.324 | 0.1936 | Yes |
| 155 | 550398 | srd5a2a |  |  | 2104 | 1.324 | 0.1945 | Yes |
| 156 | 563724 | adcy2a |  |  | 2111 | 1.321 | 0.1952 | Yes |
| 157 | 108179658\_1 | LOC108179658 |  |  | 2115 | 1.319 | 0.1960 | Yes |
| 158 | 334217\_1 | elovl7a |  |  | 2141 | 1.310 | 0.1961 | Yes |
| 159 | 393778 | prdx6 |  |  | 2148 | 1.307 | 0.1968 | Yes |
| 160 | 556849 | entpd5a |  |  | 2149 | 1.307 | 0.1977 | Yes |
| 161 | 406688\_1 | got2a |  |  | 2167 | 1.300 | 0.1980 | Yes |
| 162 | 553440 | acot20 |  |  | 2200 | 1.287 | 0.1978 | Yes |
| 163 | 571365 | gstm.2 |  |  | 2205 | 1.283 | 0.1986 | Yes |
| 164 | 563020 | hpse |  |  | 2211 | 1.282 | 0.1993 | Yes |
| 165 | 393793 | uqcrc1 |  |  | 2224 | 1.277 | 0.1998 | Yes |
| 166 | 570866 | pank2 |  |  | 2250 | 1.266 | 0.1998 | Yes |
| 167 | 436627 | bpnt1 |  |  | 2257 | 1.262 | 0.2005 | Yes |
| 168 | 767661 | blvra |  |  | 2260 | 1.261 | 0.2013 | Yes |
| 169 | 100034406 | cyp2p7 |  |  | 2273 | 1.257 | 0.2018 | Yes |
| 170 | 567170\_1 | pafah1b2 |  |  | 2274 | 1.257 | 0.2026 | Yes |
| 171 | 100334830 | kynu |  |  | 2298 | 1.249 | 0.2027 | Yes |
| 172 | 100003996 | si:ch73-195i19.3 |  |  | 2320 | 1.241 | 0.2029 | Yes |
| 173 | 562316 | kl |  |  | 2333 | 1.236 | 0.2033 | Yes |
| 174 | 393135 | zgc:55621 |  |  | 2338 | 1.233 | 0.2041 | Yes |
| 175 | 792506 | zgc:172315 |  |  | 2366 | 1.221 | 0.2040 | Yes |
| 176 | 556557 | zmp:0000001048 |  |  | 2373 | 1.220 | 0.2046 | Yes |
| 177 | 555330 | etnk2 |  |  | 2397 | 1.210 | 0.2047 | Yes |
| 178 | 280648 | rdh8a |  |  | 2400 | 1.208 | 0.2055 | Yes |
| 179 | 793635\_1 | b4galnt1b |  |  | 2414 | 1.203 | 0.2059 | Yes |
| 180 | 553981 | sptlc1 |  |  | 2420 | 1.200 | 0.2065 | Yes |
| 181 | 565370\_1 | comtb |  |  | 2427 | 1.198 | 0.2072 | Yes |
| 182 | 393191 | gpx8 |  |  | 2428 | 1.197 | 0.2080 | Yes |
| 183 | 569014 | ggt1b |  |  | 2440 | 1.194 | 0.2085 | Yes |
| 184 | 100148922 | si |  |  | 2448 | 1.191 | 0.2091 | Yes |
| 185 | 565700 | sat1a.1 |  |  | 2458 | 1.187 | 0.2096 | Yes |
| 186 | 447891 | sc5d |  |  | 2509 | 1.170 | 0.2087 | Yes |
| 187 | 751638 | cox5b2 |  |  | 2518 | 1.168 | 0.2092 | Yes |
| 188 | 100150934 | fuk |  |  | 2532 | 1.164 | 0.2096 | Yes |
| 189 | 394156\_1 | acadl |  |  | 2548 | 1.160 | 0.2099 | Yes |
| 190 | 798864\_1 | pla2g4f.1 |  |  | 2559 | 1.155 | 0.2104 | Yes |
| 191 | 619259 | dao.1 |  |  | 2605 | 1.142 | 0.2097 | Yes |
| 192 | 246227 | ptgs2a |  |  | 2612 | 1.141 | 0.2102 | Yes |
| 193 | 563642 | sdsl |  |  | 2618 | 1.139 | 0.2109 | Yes |
| 194 | 393677 | aanat1 |  |  | 2622 | 1.139 | 0.2116 | Yes |
| 195 | 100006303 | gls2a |  |  | 2628 | 1.135 | 0.2122 | Yes |
| 196 | 393350 | xylb |  |  | 2641 | 1.131 | 0.2126 | Yes |
| 197 | 100331172 | epx |  |  | 2649 | 1.129 | 0.2131 | Yes |
| 198 | 393293\_1 | hsd11b1la |  |  | 2650 | 1.129 | 0.2139 | Yes |
| 199 | 569945 | gfpt2 |  |  | 2656 | 1.125 | 0.2145 | Yes |
| 200 | 553575 | gsr |  |  | 2662 | 1.122 | 0.2151 | Yes |
| 201 | 402830 | ehmt1b |  |  | 2669 | 1.121 | 0.2157 | Yes |
| 202 | 449816 | elovl1a |  |  | 2673 | 1.120 | 0.2164 | Yes |
| 203 | 100885772 | cmasb |  |  | 2694 | 1.112 | 0.2165 | Yes |
| 204 | 100384899 | ugt1b5 |  |  | 2727 | 1.103 | 0.2161 | Yes |
| 205 | 447905 | entpd2a.1 |  |  | 2728 | 1.102 | 0.2169 | Yes |
| 206 | 394190 | hmgcl |  |  | 2729 | 1.102 | 0.2177 | Yes |
| 207 | 321224 | hkdc1 |  |  | 2737 | 1.100 | 0.2182 | Yes |
| 208 | 492476 | csad |  |  | 2749 | 1.096 | 0.2186 | Yes |
| 209 | 565785 | l3hypdh |  |  | 2770 | 1.091 | 0.2187 | Yes |
| 210 | 80374 | cyp11a1 |  |  | 2771 | 1.091 | 0.2194 | Yes |
| 211 | 359839 | atp6v1ba |  |  | 2778 | 1.087 | 0.2200 | Yes |
| 212 | 436725\_2 | acp5b |  |  | 2788 | 1.085 | 0.2204 | Yes |
| 213 | 557628\_1 | si:dkey-239i20.4 |  |  | 2797 | 1.084 | 0.2209 | Yes |
| 214 | 327274 | elovl7b |  |  | 2805 | 1.083 | 0.2214 | Yes |
| 215 | 407673\_1 | rpia |  |  | 2807 | 1.082 | 0.2221 | Yes |
| 216 | 572492 | pld1b |  |  | 2854 | 1.069 | 0.2213 | Yes |
| 217 | 114551 | pklr |  |  | 2861 | 1.068 | 0.2219 | Yes |
| 218 | 571356\_2 | pik3c2a |  |  | 2879 | 1.063 | 0.2220 | Yes |
| 219 | 333997 | ampd3b |  |  | 2884 | 1.062 | 0.2226 | Yes |
| 220 | 556535 | setd1a |  |  | 2902 | 1.058 | 0.2228 | Yes |
| 221 | 368367\_1 | hsd20b2 |  |  | 2911 | 1.057 | 0.2233 | Yes |
| 222 | 368262\_1 | dlst |  |  | 2921 | 1.056 | 0.2237 | Yes |
| 223 | 572649 | bbox1 |  |  | 2922 | 1.056 | 0.2244 | Yes |
| 224 | 321193 | paics |  |  | 2933 | 1.054 | 0.2248 | Yes |
| 225 | 394181\_1 | cox15 |  |  | 2942 | 1.052 | 0.2253 | Yes |
| 226 | 791647 | pnp5a |  |  | 2948 | 1.051 | 0.2258 | Yes |
| 227 | 559124 | ppap2d |  |  | 2971 | 1.043 | 0.2258 | Yes |
| 228 | 101886255 | LOC101886255 |  |  | 2978 | 1.042 | 0.2263 | Yes |
| 229 | 559959 | ids |  |  | 2985 | 1.041 | 0.2269 | Yes |
| 230 | 724080 | b4galt5 |  |  | 3003 | 1.035 | 0.2270 | Yes |
| 231 | 60640 | cyp19a1b |  |  | 3007 | 1.034 | 0.2276 | Yes |
| 232 | 561372 | comta |  |  | 3009 | 1.034 | 0.2283 | Yes |
| 233 | 553712\_1 | zgc:112332 |  |  | 3019 | 1.031 | 0.2287 | Yes |
| 234 | 101884069 | si:ch73-59p9.2 |  |  | 3023 | 1.030 | 0.2293 | Yes |
| 235 | 436762 | sdhda |  |  | 3054 | 1.024 | 0.2290 | Yes |
| 236 | 116991 | ugdh |  |  | 3091 | 1.016 | 0.2285 | Yes |
| 237 | 322670 | aco2 |  |  | 3094 | 1.015 | 0.2292 | Yes |
| 238 | 103909985 | LOC103909985 |  |  | 3098 | 1.013 | 0.2298 | Yes |
| 239 | 108190047 | LOC108190047 |  |  | 3138 | 1.004 | 0.2292 | Yes |
| 240 | 387530 | ahcy |  |  | 3139 | 1.004 | 0.2299 | Yes |
| 241 | 494054 | tm7sf2 |  |  | 3158 | 1.001 | 0.2299 | Yes |
| 242 | 436981\_2 | pde6d |  |  | 3163 | 1.000 | 0.2305 | Yes |
| 243 | 334270 | agk |  |  | 3184 | 0.996 | 0.2305 | Yes |
| 244 | 790919 | g6pc3 |  |  | 3212 | 0.991 | 0.2303 | Yes |
| 245 | 556439 | si:ch211-212g7.6 |  |  | 3230 | 0.987 | 0.2304 | Yes |
| 246 | 445182 | auh |  |  | 3246 | 0.983 | 0.2306 | Yes |
| 247 | 100037342 | acadsb |  |  | 3276 | 0.978 | 0.2303 | Yes |
| 248 | 393622 | acsl4a |  |  | 3304 | 0.972 | 0.2300 | Yes |
| 249 | 562627\_1 | nt5c1aa |  |  | 3317 | 0.969 | 0.2303 | Yes |
| 250 | 566479 | plch1 |  |  | 3324 | 0.968 | 0.2308 | Yes |
| 251 | 394083 | slc33a1 |  |  | 3334 | 0.966 | 0.2312 | Yes |
| 252 | 100144569 | dpys |  |  | 3351 | 0.963 | 0.2313 | Yes |
| 253 | 791613\_1 | ugt2a4 |  |  | 3374 | 0.959 | 0.2312 | Yes |
| 254 | 556544\_1 | si:dkey-199f5.8 |  |  | 3380 | 0.958 | 0.2317 | Yes |
| 255 | 436914 | acss2 |  |  | 3398 | 0.954 | 0.2318 | Yes |
| 256 | 550524 | cbsa |  |  | 3402 | 0.952 | 0.2324 | Yes |
| 257 | 406338 | chia.2 |  |  | 3412 | 0.950 | 0.2327 | Yes |
| 258 | 553676 | mgat3a |  |  | 3418 | 0.948 | 0.2332 | Yes |
| 259 | 553804 | mcee |  |  | 3424 | 0.947 | 0.2337 | Yes |
| 260 | 570720 | cept1b |  |  | 3426 | 0.946 | 0.2343 | Yes |
| 261 | 100384895 | ugt2b1 |  |  | 3468 | 0.936 | 0.2336 | Yes |
| 262 | 565122 | si:dkey-172j4.3 |  |  | 3472 | 0.936 | 0.2341 | Yes |
| 263 | 100415799 | ugt5b2 |  |  | 3477 | 0.935 | 0.2346 | Yes |
| 264 | 558239 | si:ch211-113j14.1 |  |  | 3492 | 0.932 | 0.2348 | Yes |
| 265 | 321224\_1 | hkdc1 |  |  | 3521 | 0.925 | 0.2345 | Yes |
| 266 | 100384898 | ugt2b3 |  |  | 3526 | 0.924 | 0.2350 | Yes |
| 267 | 559072 | gba |  |  | 3533 | 0.923 | 0.2355 | Yes |
| 268 | 352943\_1 | tph1a |  |  | 3540 | 0.921 | 0.2359 | Yes |
| 269 | 792824 | pgp |  |  | 3560 | 0.918 | 0.2359 | Yes |
| 270 | 327590 | tk1 |  |  | 3561 | 0.918 | 0.2365 | Yes |
| 271 | 570652 | adcy6a |  |  | 3581 | 0.914 | 0.2365 | Yes |
| 272 | 492773 | pomk |  |  | 3622 | 0.907 | 0.2358 | Yes |
| 273 | 30685 | aanat2 |  |  | 3624 | 0.907 | 0.2364 | Yes |
| 274 | 100002310 | si:dkey-152b24.6 |  |  | 3649 | 0.902 | 0.2362 | Yes |
| 275 | 751668 | gck |  |  | 3655 | 0.901 | 0.2367 | Yes |
| 276 | 767750 | atp5ib |  |  | 3673 | 0.897 | 0.2367 | Yes |
| 277 | 557324 | dgkb |  |  | 3678 | 0.897 | 0.2372 | Yes |
| 278 | 493783 | extl3 |  |  | 3713 | 0.889 | 0.2367 | Yes |
| 279 | 393657 | zgc:66024 |  |  | 3724 | 0.885 | 0.2370 | Yes |
| 280 | 407614 | alg11 |  |  | 3750 | 0.878 | 0.2367 | Yes |
| 281 | 554143 | gnpnat1 |  |  | 3790 | 0.869 | 0.2360 | Yes |
| 282 | 550425 | b4galt1 |  |  | 3791 | 0.869 | 0.2366 | Yes |
| 283 | 324947 | sephs1 |  |  | 3798 | 0.868 | 0.2370 | Yes |
| 284 | 793524\_1 | ndst2a |  |  | 3801 | 0.868 | 0.2376 | Yes |
| 285 | 572324 | b3galt6 |  |  | 3811 | 0.866 | 0.2379 | Yes |
| 286 | 404731 | uros |  |  | 3827 | 0.863 | 0.2379 | Yes |
| 287 | 327506\_1 | ckmt2a |  |  | 3841 | 0.861 | 0.2381 | Yes |
| 288 | 100004557 | hyal3 |  |  | 3842 | 0.861 | 0.2387 | Yes |
| 289 | 570828 | mgat3b |  |  | 3843 | 0.861 | 0.2393 | Yes |
| 290 | 555725 | pcyt1bb |  |  | 3857 | 0.858 | 0.2395 | Yes |
| 291 | 337333 | chia.4 |  |  | 3858 | 0.858 | 0.2401 | Yes |
| 292 | 445288 | acads |  |  | 3897 | 0.848 | 0.2394 | Yes |
| 293 | 406662 | msmo1 |  |  | 3921 | 0.844 | 0.2392 | Yes |
| 294 | 678614 | elovl2 |  |  | 3951 | 0.839 | 0.2388 | Yes |
| 295 | 406696 | pgk1 |  |  | 3987 | 0.833 | 0.2382 | Yes |
| 296 | 560682 | pycr3 |  |  | 4000 | 0.831 | 0.2384 | Yes |
| 297 | 567861 | gfpt1 |  |  | 4078 | 0.815 | 0.2363 | Yes |
| 298 | 793362 | b3gnt2b |  |  | 4115 | 0.809 | 0.2357 | Yes |
| 299 | 556667\_1 | pigt |  |  | 4138 | 0.803 | 0.2355 | Yes |
| 300 | 326906 | suv39h1b |  |  | 4156 | 0.801 | 0.2355 | Yes |
| 301 | 406278 | ggctb |  |  | 4161 | 0.800 | 0.2359 | Yes |
| 302 | 402802 | pla2g12a |  |  | 4164 | 0.800 | 0.2364 | Yes |
| 303 | 30643 | acat2 |  |  | 4174 | 0.798 | 0.2366 | Yes |
| 304 | 394114 | kdsr |  |  | 4175 | 0.797 | 0.2372 | Yes |
| 305 | 101886093 | si:ch211-264e16.1 |  |  | 4181 | 0.797 | 0.2376 | Yes |
| 306 | 793249\_1 | cyp21a2 |  |  | 4183 | 0.796 | 0.2381 | Yes |
| 307 | 393931 | ptdss1a |  |  | 4185 | 0.796 | 0.2386 | Yes |
| 308 | 767665 | galnt10 |  |  | 4188 | 0.796 | 0.2391 | Yes |
| 309 | 436672 | pdha1b |  |  | 4206 | 0.792 | 0.2391 | Yes |
| 310 | 393448 | pigb |  |  | 4214 | 0.791 | 0.2394 | Yes |
| 311 | 100317129 | ndst3 |  |  | 4216 | 0.790 | 0.2399 | Yes |
| 312 | 565370 | comtb |  |  | 4234 | 0.788 | 0.2399 | Yes |
| 313 | 406830 | zgc:55413 |  |  | 4240 | 0.787 | 0.2402 | Yes |
| 314 | 100536873 | acot12 |  |  | 4243 | 0.787 | 0.2407 | Yes |
| 315 | 100004018\_1 | si:ch211-198n5.11 |  |  | 4269 | 0.781 | 0.2404 | Yes |
| 316 | 393997 | lipca |  |  | 4285 | 0.778 | 0.2405 | Yes |
| 317 | 406477 | galnt6 |  |  | 4301 | 0.776 | 0.2405 | Yes |
| 318 | 562007 | irg1l |  |  | 4311 | 0.774 | 0.2407 | Yes |
| 319 | 570547 | uckl1a |  |  | 4313 | 0.774 | 0.2412 | Yes |
| 320 | 334420\_1 | ugp2b |  |  | 4329 | 0.772 | 0.2413 | Yes |
| 321 | 568645 | LOC568645 |  |  | 4335 | 0.772 | 0.2416 | Yes |
| 322 | 791612 | mat2ab |  |  | 4341 | 0.771 | 0.2420 | Yes |
| 323 | 793926 | tkfc |  |  | 4368 | 0.766 | 0.2417 | Yes |
| 324 | 558050 | si:ch211-127i16.2 |  |  | 4370 | 0.766 | 0.2421 | Yes |
| 325 | 406605 | fam213b |  |  | 4383 | 0.764 | 0.2423 | Yes |
| 326 | 563347 | si:ch211-264e16.2 |  |  | 4389 | 0.764 | 0.2426 | Yes |
| 327 | 777716\_1 | ocrl |  |  | 4402 | 0.760 | 0.2428 | Yes |
| 328 | 571274 | setd1bb |  |  | 4438 | 0.755 | 0.2421 | Yes |
| 329 | 436692 | entpd4 |  |  | 4461 | 0.750 | 0.2419 | Yes |
| 330 | 30068 | cat |  |  | 4462 | 0.750 | 0.2424 | Yes |
| 331 | 569568\_1 | LOC569568 |  |  | 4465 | 0.750 | 0.2429 | Yes |
| 332 | 566188 | inpp5kb |  |  | 4470 | 0.749 | 0.2432 | Yes |
| 333 | 560944 | pfkpa |  |  | 4480 | 0.748 | 0.2435 | Yes |
| 334 | 494056 | zgc:103559 |  |  | 4489 | 0.746 | 0.2437 | Yes |
| 335 | 564739 | LOC564739 |  |  | 4494 | 0.746 | 0.2441 | Yes |
| 336 | 796720 | si:ch211-263k4.2 |  |  | 4504 | 0.744 | 0.2443 | Yes |
| 337 | 447879 | nmrk2 |  |  | 4508 | 0.743 | 0.2447 | Yes |
| 338 | 100000859 | ehhadh |  |  | 4565 | 0.737 | 0.2433 | Yes |
| 339 | 497280 | ext1b |  |  | 4567 | 0.737 | 0.2438 | Yes |
| 340 | 565316 | dgat2 |  |  | 4579 | 0.736 | 0.2440 | Yes |
| 341 | 565385 | zgc:172341 |  |  | 4587 | 0.735 | 0.2442 | Yes |
| 342 | 405822\_1 | pgm2 |  |  | 4597 | 0.735 | 0.2444 | Yes |
| 343 | 553795\_1 | nanp |  |  | 4600 | 0.734 | 0.2449 | Yes |
| 344 | 553384 | cox10 |  |  | 4639 | 0.729 | 0.2441 | Yes |
| 345 | 492359\_1 | zgc:91976 |  |  | 4674 | 0.723 | 0.2435 | Yes |
| 346 | 792268 | galk2 |  |  | 4685 | 0.722 | 0.2436 | Yes |
| 347 | 723997 | mgst2 |  |  | 4718 | 0.716 | 0.2430 | Yes |
| 348 | 335732 | elovl4b |  |  | 4729 | 0.714 | 0.2432 | Yes |
| 349 | 322720 | itpkcb |  |  | 4736 | 0.713 | 0.2435 | Yes |
| 350 | 567547 | mthfr |  |  | 4740 | 0.713 | 0.2439 | Yes |
| 351 | 564592 | nsd1b |  |  | 4750 | 0.712 | 0.2441 | Yes |
| 352 | 103911184 | chpfb |  |  | 4760 | 0.711 | 0.2443 | Yes |
| 353 | 797914 | smpd2b |  |  | 4771 | 0.709 | 0.2444 | Yes |
| 354 | 100004076 | rpe65c |  |  | 4773 | 0.709 | 0.2449 | Yes |
| 355 | 558499\_1 | chka |  |  | 4788 | 0.707 | 0.2449 | Yes |
| 356 | 798349\_1 | zgc:194330 |  |  | 4809 | 0.704 | 0.2447 | Yes |
| 357 | 560242 | pigu |  |  | 4816 | 0.702 | 0.2450 | Yes |
| 358 | 568001\_3 | pfkmb |  |  | 4822 | 0.702 | 0.2453 | Yes |
| 359 | 559087 | pla2g4ab |  |  | 4877 | 0.697 | 0.2440 | Yes |
| 360 | 562292\_2 | acsf3 |  |  | 4884 | 0.696 | 0.2443 | Yes |
| 361 | 100537538 | LOC100537538 |  |  | 4897 | 0.694 | 0.2443 | Yes |
| 362 | 541517 | mtmr1b |  |  | 4918 | 0.691 | 0.2442 | Yes |
| 363 | 393559 | gmps |  |  | 4958 | 0.686 | 0.2433 | Yes |
| 364 | 100535692 | galnt8b.2 |  |  | 4969 | 0.685 | 0.2435 | Yes |
| 365 | 564719 | adcy3b |  |  | 4985 | 0.683 | 0.2434 | Yes |
| 366 | 557187\_1 | tmem5 |  |  | 4999 | 0.681 | 0.2435 | Yes |
| 367 | 100006023 | acsbg1 |  |  | 5003 | 0.680 | 0.2438 | Yes |
| 368 | 322614 | arg2 |  |  | 5009 | 0.680 | 0.2441 | Yes |
| 369 | 791448 | qdprb1 |  |  | 5012 | 0.680 | 0.2445 | Yes |
| 370 | 492518 | haao |  |  | 5021 | 0.679 | 0.2447 | Yes |
| 371 | 550388 | srd5a2b |  |  | 5026 | 0.679 | 0.2451 | Yes |
| 372 | 445201 | cant1b |  |  | 5037 | 0.677 | 0.2452 | Yes |
| 373 | 553722 | gba3 |  |  | 5038 | 0.676 | 0.2457 | Yes |
| 374 | 554124 | bckdha |  |  | 5080 | 0.670 | 0.2448 | Yes |
| 375 | 554100 | ndufs8b |  |  | 5088 | 0.669 | 0.2450 | Yes |
| 376 | 564694 | ckba |  |  | 5094 | 0.668 | 0.2453 | Yes |
| 377 | 447895 | gpx1b |  |  | 5104 | 0.666 | 0.2454 | Yes |
| 378 | 567728\_1 | LOC567728 |  |  | 5105 | 0.666 | 0.2459 | Yes |
| 379 | 559403 | acaca |  |  | 5108 | 0.666 | 0.2463 | Yes |
| 380 | 100037312 | sgpl1 |  |  | 5110 | 0.666 | 0.2467 | Yes |
| 381 | 100148828 | b4galt2 |  |  | 5115 | 0.666 | 0.2471 | Yes |
| 382 | 100005587 | aldh9a1a.1 |  |  | 5134 | 0.663 | 0.2469 | Yes |
| 383 | 101882461 | LOC101882461 |  |  | 5142 | 0.662 | 0.2471 | Yes |
| 384 | 559124\_1 | ppap2d |  |  | 5172 | 0.659 | 0.2466 | Yes |
| 385 | 553169 | gstp2 |  |  | 5188 | 0.656 | 0.2466 | Yes |
| 386 | 568219 | si:ch73-139e5.4 |  |  | 5190 | 0.656 | 0.2470 | Yes |
| 387 | 100332784 | si:ch73-12o23.1 |  |  | 5195 | 0.655 | 0.2473 | Yes |
| 388 | 791189 | hyal6 |  |  | 5269 | 0.649 | 0.2453 | No |
| 389 | 393906 | nt5e |  |  | 5288 | 0.648 | 0.2451 | No |
| 390 | 100332609 | LOC100332609 |  |  | 5295 | 0.647 | 0.2454 | No |
| 391 | 567414\_2 | agpat9l |  |  | 5326 | 0.645 | 0.2448 | No |
| 392 | 100536819 | LOC100536819 |  |  | 5333 | 0.645 | 0.2451 | No |
| 393 | 559212 | gbe1b |  |  | 5390 | 0.640 | 0.2436 | No |
| 394 | 565386\_1 | pla2g4f.2 |  |  | 5440 | 0.638 | 0.2424 | No |
| 395 | 564675 | paox1 |  |  | 5446 | 0.638 | 0.2427 | No |
| 396 | 553653 | amacr |  |  | 5466 | 0.636 | 0.2425 | No |
| 397 | 100148581 | LOC100148581 |  |  | 5502 | 0.634 | 0.2418 | No |
| 398 | 101669768 | b4gat1 |  |  | 5505 | 0.634 | 0.2421 | No |
| 399 | 562149 | sdhb |  |  | 5566 | 0.630 | 0.2406 | No |
| 400 | 799964\_1 | ptgesl |  |  | 5581 | 0.629 | 0.2405 | No |
| 401 | 393264\_1 | uap1l1 |  |  | 5582 | 0.629 | 0.2410 | No |
| 402 | 497407 | mecom |  |  | 5585 | 0.628 | 0.2413 | No |
| 403 | novel.328 | - |  |  | 5616 | 0.624 | 0.2407 | No |
| 404 | 560919 | plch2b |  |  | 5624 | 0.623 | 0.2409 | No |
| 405 | 556237\_1 | man1b1b |  |  | 5631 | 0.623 | 0.2412 | No |
| 406 | 405829 | dpydb |  |  | 5638 | 0.623 | 0.2414 | No |
| 407 | 794084 | si:ch73-111m19.2 |  |  | 5639 | 0.623 | 0.2418 | No |
| 408 | 394148 | cyp7a1 |  |  | 5651 | 0.622 | 0.2419 | No |
| 409 | 798974\_1 | nadsyn1 |  |  | 5668 | 0.620 | 0.2418 | No |
| 410 | 553532\_1 | hsd3b1 |  |  | 5680 | 0.620 | 0.2418 | No |
| 411 | 393108 | cyp2p6 |  |  | 5701 | 0.618 | 0.2416 | No |
| 412 | 103910015 | LOC103910015 |  |  | 5729 | 0.614 | 0.2411 | No |
| 413 | 561747 | plcg2 |  |  | 5761 | 0.610 | 0.2405 | No |
| 414 | 404730 | mao |  |  | 5766 | 0.610 | 0.2408 | No |
| 415 | 794311 | fmo5 |  |  | 5793 | 0.608 | 0.2403 | No |
| 416 | 569568 | LOC569568 |  |  | 5824 | 0.605 | 0.2397 | No |
| 417 | 791210 | ugt5b4 |  |  | 5827 | 0.605 | 0.2401 | No |
| 418 | 447860 | acsl5 |  |  | 5835 | 0.604 | 0.2403 | No |
| 419 | 317746\_1 | suclg2 |  |  | 5836 | 0.604 | 0.2407 | No |
| 420 | 334082 | tdo2b |  |  | 5850 | 0.603 | 0.2407 | No |
| 421 | 100008541 | tdo2a |  |  | 5876 | 0.600 | 0.2402 | No |
| 422 | 494102 | dhcr24 |  |  | 5884 | 0.598 | 0.2404 | No |
| 423 | 337696 | cbr1l |  |  | 5935 | 0.594 | 0.2392 | No |
| 424 | 100038790 | zgc:163121 |  |  | 5957 | 0.591 | 0.2389 | No |
| 425 | 560638 | si:ch73-21k16.1 |  |  | 5961 | 0.591 | 0.2392 | No |
| 426 | 100384893 | ugt1b2 |  |  | 5989 | 0.588 | 0.2387 | No |
| 427 | 393160 | tigarb |  |  | 6007 | 0.585 | 0.2385 | No |
| 428 | 557166 | acmsd |  |  | 6016 | 0.585 | 0.2386 | No |
| 429 | 563897 | abo |  |  | 6025 | 0.584 | 0.2388 | No |
| 430 | 100001620 | LOC100001620 |  |  | 6052 | 0.582 | 0.2383 | No |
| 431 | 799963 | gpt2 |  |  | 6058 | 0.581 | 0.2385 | No |
| 432 | 110438373 | LOC110438373 |  |  | 6078 | 0.579 | 0.2383 | No |
| 433 | 494077 | tsta3 |  |  | 6091 | 0.577 | 0.2383 | No |
| 434 | 572518 | oat |  |  | 6095 | 0.577 | 0.2386 | No |
| 435 | 100330415 | si:dkey-96f10.1 |  |  | 6101 | 0.577 | 0.2388 | No |
| 436 | 445281 | cyp8b1 |  |  | 6109 | 0.576 | 0.2390 | No |
| 437 | 563855 | chac1 |  |  | 6138 | 0.573 | 0.2384 | No |
| 438 | 796487 | cyp17a2 |  |  | 6151 | 0.572 | 0.2384 | No |
| 439 | 566998 | pde4d |  |  | 6203 | 0.568 | 0.2371 | No |
| 440 | 402994 | dao.3 |  |  | 6229 | 0.565 | 0.2367 | No |
| 441 | 100333757 | ggt5b |  |  | 6235 | 0.564 | 0.2369 | No |
| 442 | 436981\_1 | pde6d |  |  | 6269 | 0.560 | 0.2362 | No |
| 443 | 563806 | plpp2a |  |  | 6278 | 0.559 | 0.2363 | No |
| 444 | 449662 | acox1 |  |  | 6280 | 0.559 | 0.2366 | No |
| 445 | 406819 | chia.3 |  |  | 6288 | 0.558 | 0.2368 | No |
| 446 | 553508\_1 | ugt8 |  |  | 6332 | 0.554 | 0.2357 | No |
| 447 | 553189 | dnmt3ab |  |  | 6369 | 0.550 | 0.2349 | No |
| 448 | 108181664 | LOC108181664 |  |  | 6378 | 0.549 | 0.2350 | No |
| 449 | 436604 | uox |  |  | 6380 | 0.549 | 0.2354 | No |
| 450 | 100002302 | mri1 |  |  | 6386 | 0.548 | 0.2356 | No |
| 451 | 571263 | acyp1 |  |  | 6391 | 0.548 | 0.2358 | No |
| 452 | 100002812 | phospho1 |  |  | 6409 | 0.545 | 0.2356 | No |
| 453 | 556538\_1 | ndufa12 |  |  | 6421 | 0.544 | 0.2356 | No |
| 454 | 393528 | lias |  |  | 6431 | 0.543 | 0.2357 | No |
| 455 | 337583 | atp6v1ab |  |  | 6443 | 0.542 | 0.2357 | No |
| 456 | 437016 | tk2 |  |  | 6454 | 0.541 | 0.2357 | No |
| 457 | 393810 | zgc:73359 |  |  | 6510 | 0.536 | 0.2343 | No |
| 458 | 100005158\_1 | cox11 |  |  | 6560 | 0.532 | 0.2330 | No |
| 459 | 553270 | pde5ab |  |  | 6563 | 0.531 | 0.2333 | No |
| 460 | 368909 | rrm2b |  |  | 6644 | 0.524 | 0.2310 | No |
| 461 | 30384 | th |  |  | 6648 | 0.524 | 0.2312 | No |
| 462 | 794311\_1 | fmo5 |  |  | 6655 | 0.523 | 0.2314 | No |
| 463 | 336606 | hadhb |  |  | 6664 | 0.523 | 0.2315 | No |
| 464 | 792527 | inpp4ab |  |  | 6677 | 0.522 | 0.2314 | No |
| 465 | 100073330 | pts |  |  | 6682 | 0.521 | 0.2317 | No |
| 466 | 399481 | aldh9a1b |  |  | 6718 | 0.518 | 0.2308 | No |
| 467 | 445134 | zgc:100864 |  |  | 6732 | 0.516 | 0.2308 | No |
| 468 | 100005551 | entpd3 |  |  | 6753 | 0.515 | 0.2304 | No |
| 469 | 570579\_2 | g6pd |  |  | 6769 | 0.513 | 0.2303 | No |
| 470 | 100329531 | hmox2b |  |  | 6780 | 0.512 | 0.2303 | No |
| 471 | 58215 | fech |  |  | 6801 | 0.510 | 0.2300 | No |
| 472 | 798291 | atp5e |  |  | 6822 | 0.508 | 0.2297 | No |
| 473 | 100415798 | ugt5a4 |  |  | 6826 | 0.508 | 0.2299 | No |
| 474 | 393700 | ndufa8 |  |  | 6830 | 0.507 | 0.2302 | No |
| 475 | 322732 | alox12 |  |  | 6866 | 0.503 | 0.2293 | No |
| 476 | 100330514 | pik3c2g |  |  | 6869 | 0.503 | 0.2296 | No |
| 477 | 568099 | dsela |  |  | 6882 | 0.502 | 0.2296 | No |
| 478 | 556578 | ampd3a |  |  | 6885 | 0.501 | 0.2298 | No |
| 479 | 565385\_1 | zgc:172341 |  |  | 6886 | 0.501 | 0.2302 | No |
| 480 | 570478 | cmpk2 |  |  | 6893 | 0.501 | 0.2303 | No |
| 481 | 795050 | ugt1b4 |  |  | 6900 | 0.500 | 0.2305 | No |
| 482 | 541423 | smyd2a |  |  | 6906 | 0.500 | 0.2307 | No |
| 483 | 317737\_1 | glud1a |  |  | 6910 | 0.499 | 0.2309 | No |
| 484 | 100536162 | pde1cb |  |  | 6926 | 0.498 | 0.2307 | No |
| 485 | 322811 | atp6v0d1 |  |  | 6941 | 0.497 | 0.2306 | No |
| 486 | 436747 | ldhbb |  |  | 6973 | 0.494 | 0.2299 | No |
| 487 | 553433\_1 | pisd |  |  | 6981 | 0.493 | 0.2300 | No |
| 488 | 558373 | extl2 |  |  | 6983 | 0.493 | 0.2303 | No |
| 489 | 497644 | pomgnt2 |  |  | 6985 | 0.493 | 0.2306 | No |
| 490 | 799492\_1 | ckmt2b |  |  | 6992 | 0.492 | 0.2308 | No |
| 491 | 100004076\_1 | rpe65c |  |  | 7007 | 0.491 | 0.2307 | No |
| 492 | 100334730 | ganab |  |  | 7025 | 0.490 | 0.2304 | No |
| 493 | 564389 | pde9al |  |  | 7032 | 0.489 | 0.2306 | No |
| 494 | 282672 | fbp1b |  |  | 7040 | 0.488 | 0.2307 | No |
| 495 | 406290 | itpk1a |  |  | 7047 | 0.488 | 0.2308 | No |
| 496 | 562316\_1 | kl |  |  | 7050 | 0.488 | 0.2311 | No |
| 497 | 777635 | plod1a |  |  | 7064 | 0.486 | 0.2310 | No |
| 498 | 564465 | pde1a |  |  | 7073 | 0.485 | 0.2310 | No |
| 499 | 450072 | alg14 |  |  | 7077 | 0.485 | 0.2313 | No |
| 500 | 751651 | tmem86b |  |  | 7080 | 0.485 | 0.2315 | No |
| 501 | 30086 | nme7 |  |  | 7162 | 0.476 | 0.2291 | No |
| 502 | 553671\_1 | ak8 |  |  | 7165 | 0.476 | 0.2294 | No |
| 503 | 30554 | pla2g4aa |  |  | 7169 | 0.476 | 0.2296 | No |
| 504 | 558422 | lipin2 |  |  | 7174 | 0.475 | 0.2298 | No |
| 505 | 100005054 | lacc1 |  |  | 7184 | 0.475 | 0.2299 | No |
| 506 | 561737 | pik3ca |  |  | 7195 | 0.474 | 0.2298 | No |
| 507 | 565372 | hexdc |  |  | 7210 | 0.473 | 0.2297 | No |
| 508 | 336977 | hagh |  |  | 7212 | 0.473 | 0.2300 | No |
| 509 | 436603 | agxta |  |  | 7216 | 0.472 | 0.2302 | No |
| 510 | 570579 | g6pd |  |  | 7224 | 0.472 | 0.2303 | No |
| 511 | 553246 | ca4b |  |  | 7290 | 0.467 | 0.2285 | No |
| 512 | 100333524 | LOC100333524 |  |  | 7295 | 0.467 | 0.2286 | No |
| 513 | 558437 | gpat2 |  |  | 7304 | 0.466 | 0.2287 | No |
| 514 | 559103\_3 | inpp5d |  |  | 7318 | 0.465 | 0.2286 | No |
| 515 | 558684 | b4galnt1a |  |  | 7354 | 0.463 | 0.2277 | No |
| 516 | 563347\_1 | si:ch211-264e16.2 |  |  | 7362 | 0.462 | 0.2278 | No |
| 517 | 449829 | galk1 |  |  | 7365 | 0.462 | 0.2281 | No |
| 518 | 553734 | scdb |  |  | 7381 | 0.461 | 0.2279 | No |
| 519 | 450054\_1 | tpk1 |  |  | 7382 | 0.461 | 0.2282 | No |
| 520 | 791167\_1 | zgc:158222 |  |  | 7399 | 0.460 | 0.2280 | No |
| 521 | 431723 | tha1 |  |  | 7437 | 0.458 | 0.2270 | No |
| 522 | 326857 | gclc |  |  | 7445 | 0.457 | 0.2271 | No |
| 523 | 402939 | zgc:77938 |  |  | 7452 | 0.457 | 0.2272 | No |
| 524 | 559148\_1 | ptgis |  |  | 7474 | 0.455 | 0.2269 | No |
| 525 | 767705 | ugt2a5 |  |  | 7477 | 0.455 | 0.2271 | No |
| 526 | 140426 | gc3 |  |  | 7516 | 0.453 | 0.2261 | No |
| 527 | 450077 | dera |  |  | 7535 | 0.453 | 0.2258 | No |
| 528 | 100329505 | hgsnat |  |  | 7569 | 0.450 | 0.2250 | No |
| 529 | 474321 | pgm3 |  |  | 7570 | 0.450 | 0.2254 | No |
| 530 | 553701 | gda |  |  | 7572 | 0.450 | 0.2256 | No |
| 531 | 414844 | th2 |  |  | 7591 | 0.448 | 0.2253 | No |
| 532 | 64674 | mibp |  |  | 7595 | 0.448 | 0.2256 | No |
| 533 | 404633 | b3galt2 |  |  | 7596 | 0.448 | 0.2259 | No |
| 534 | 566019 | acss3 |  |  | 7611 | 0.446 | 0.2257 | No |
| 535 | 562047 | acot21 |  |  | 7621 | 0.445 | 0.2257 | No |
| 536 | 566078 | man1a1 |  |  | 7623 | 0.445 | 0.2260 | No |
| 537 | 548343 | glb1 |  |  | 7702 | 0.440 | 0.2237 | No |
| 538 | 402842 | hsd17b1 |  |  | 7757 | 0.439 | 0.2221 | No |
| 539 | 415103 | tph1b |  |  | 7790 | 0.438 | 0.2214 | No |
| 540 | 798864 | pla2g4f.1 |  |  | 7791 | 0.438 | 0.2217 | No |
| 541 | 324900 | txndc12 |  |  | 7806 | 0.437 | 0.2215 | No |
| 542 | 567734 | dtymk |  |  | 7842 | 0.436 | 0.2206 | No |
| 543 | 387526 | ca2 |  |  | 7850 | 0.435 | 0.2207 | No |
| 544 | 394151 | atp5g3a |  |  | 7852 | 0.435 | 0.2210 | No |
| 545 | 550420\_1 | gucy1a3 |  |  | 7860 | 0.434 | 0.2210 | No |
| 546 | 565706\_1 | pde4ba |  |  | 7871 | 0.433 | 0.2210 | No |
| 547 | 795901 | treh |  |  | 7891 | 0.432 | 0.2207 | No |
| 548 | 436959 | blvrb |  |  | 7897 | 0.431 | 0.2208 | No |
| 549 | 431773\_2 | enoph1 |  |  | 7905 | 0.431 | 0.2208 | No |
| 550 | 393753\_1 | ppcdc |  |  | 7969 | 0.426 | 0.2190 | No |
| 551 | 402799 | degs2 |  |  | 7971 | 0.425 | 0.2193 | No |
| 552 | 795305 | irg1 |  |  | 7978 | 0.425 | 0.2194 | No |
| 553 | 796135 | sirt7 |  |  | 7994 | 0.424 | 0.2192 | No |
| 554 | 436838 | pigh |  |  | 8041 | 0.420 | 0.2179 | No |
| 555 | 335485 | plpp2b |  |  | 8056 | 0.419 | 0.2177 | No |
| 556 | 550332 | dctd |  |  | 8065 | 0.418 | 0.2177 | No |
| 557 | 791613 | ugt2a4 |  |  | 8092 | 0.417 | 0.2172 | No |
| 558 | 553716\_1 | b3galnt2 |  |  | 8120 | 0.417 | 0.2165 | No |
| 559 | 436672\_1 | pdha1b |  |  | 8125 | 0.417 | 0.2167 | No |
| 560 | 557059\_2 | zgc:152670 |  |  | 8127 | 0.417 | 0.2170 | No |
| 561 | 777716 | ocrl |  |  | 8170 | 0.415 | 0.2158 | No |
| 562 | 550486 | zgc:110348 |  |  | 8180 | 0.415 | 0.2158 | No |
| 563 | 557817\_2 | enpp7.2 |  |  | 8197 | 0.415 | 0.2156 | No |
| 564 | 415152 | gyg1b |  |  | 8212 | 0.414 | 0.2154 | No |
| 565 | 557756 | enpp7.1 |  |  | 8256 | 0.414 | 0.2142 | No |
| 566 | 560807\_1 | adcy6b |  |  | 8280 | 0.414 | 0.2137 | No |
| 567 | 795445 | sprb |  |  | 8292 | 0.413 | 0.2136 | No |
| 568 | 140620 | prps1a |  |  | 8296 | 0.413 | 0.2138 | No |
| 569 | 492549 | pip5k1ab |  |  | 8298 | 0.413 | 0.2141 | No |
| 570 | 570552 | si:dkey-103j14.5 |  |  | 8327 | 0.411 | 0.2134 | No |
| 571 | 108179064 | LOC108179064 |  |  | 8408 | 0.410 | 0.2110 | No |
| 572 | 606579\_1 | inpp5e |  |  | 8420 | 0.409 | 0.2109 | No |
| 573 | 445245 | ctps1b |  |  | 8427 | 0.408 | 0.2110 | No |
| 574 | 406598 | pik3cg |  |  | 8441 | 0.407 | 0.2109 | No |
| 575 | 406330 | got1 |  |  | 8454 | 0.406 | 0.2107 | No |
| 576 | 751093 | mif |  |  | 8471 | 0.405 | 0.2105 | No |
| 577 | 406433 | dhdhl |  |  | 8472 | 0.405 | 0.2108 | No |
| 578 | 323107 | pgam1a |  |  | 8482 | 0.404 | 0.2107 | No |
| 579 | 100006589 | idh1 |  |  | 8531 | 0.401 | 0.2094 | No |
| 580 | 100333135 | si:ch1073-325m22.2 |  |  | 8533 | 0.400 | 0.2096 | No |
| 581 | 100037326 | pnpla4 |  |  | 8535 | 0.400 | 0.2099 | No |
| 582 | 100333350 | gucy2g |  |  | 8581 | 0.397 | 0.2086 | No |
| 583 | 445327 | alg6 |  |  | 8600 | 0.395 | 0.2083 | No |
| 584 | 324052 | c1galt1c1 |  |  | 8612 | 0.394 | 0.2082 | No |
| 585 | 393462 | aldh2.1 |  |  | 8615 | 0.394 | 0.2084 | No |
| 586 | 791470 | b3gnt5b |  |  | 8616 | 0.394 | 0.2087 | No |
| 587 | 407716 | st6galnac5b |  |  | 8634 | 0.393 | 0.2084 | No |
| 588 | 407706\_1 | st6galnac |  |  | 8646 | 0.392 | 0.2083 | No |
| 589 | 327496 | mthfd2l |  |  | 8655 | 0.391 | 0.2083 | No |
| 590 | 561338 | pde11a |  |  | 8747 | 0.385 | 0.2055 | No |
| 591 | 799960 | dolk |  |  | 8753 | 0.384 | 0.2056 | No |
| 592 | 368367 | hsd20b2 |  |  | 8757 | 0.384 | 0.2058 | No |
| 593 | 572649\_1 | bbox1 |  |  | 8772 | 0.383 | 0.2056 | No |
| 594 | 568288 | plce1 |  |  | 8774 | 0.383 | 0.2058 | No |
| 595 | 100136853 | pigw |  |  | 8782 | 0.382 | 0.2058 | No |
| 596 | 566612 | ca9 |  |  | 8809 | 0.380 | 0.2052 | No |
| 597 | 100000026 | aldh3a1 |  |  | 8825 | 0.379 | 0.2050 | No |
| 598 | 558601 | pipox |  |  | 8826 | 0.379 | 0.2052 | No |
| 599 | 445151 | entpd1 |  |  | 8827 | 0.379 | 0.2055 | No |
| 600 | 100005033 | si:ch211-243a15.1 |  |  | 8828 | 0.379 | 0.2058 | No |
| 601 | 415239 | pigk |  |  | 8846 | 0.378 | 0.2054 | No |
| 602 | 550580 | sqrdl |  |  | 8852 | 0.377 | 0.2055 | No |
| 603 | 767675 | pigs |  |  | 8855 | 0.377 | 0.2057 | No |
| 604 | 100004770 | setd8b |  |  | 8862 | 0.377 | 0.2058 | No |
| 605 | 560653 | npr1a |  |  | 8865 | 0.377 | 0.2060 | No |
| 606 | 415247\_1 | idh3b |  |  | 8869 | 0.377 | 0.2061 | No |
| 607 | 558487 | eno4 |  |  | 8873 | 0.376 | 0.2063 | No |
| 608 | 450063 | mogat2 |  |  | 8878 | 0.376 | 0.2064 | No |
| 609 | 100538266 | LOC100538266 |  |  | 8883 | 0.375 | 0.2066 | No |
| 610 | 568169 | cers1 |  |  | 8910 | 0.374 | 0.2059 | No |
| 611 | 406470 | pla2g7 |  |  | 8917 | 0.373 | 0.2060 | No |
| 612 | 322482 | adh8a |  |  | 8920 | 0.373 | 0.2062 | No |
| 613 | 560126 | naglu |  |  | 8936 | 0.372 | 0.2059 | No |
| 614 | 550420 | gucy1a3 |  |  | 8949 | 0.371 | 0.2058 | No |
| 615 | 402940 | pnp4b |  |  | 8950 | 0.371 | 0.2061 | No |
| 616 | 474331 | dolpp1 |  |  | 8961 | 0.370 | 0.2060 | No |
| 617 | 387303 | gbgt1l4 |  |  | 9009 | 0.367 | 0.2046 | No |
| 618 | 555864\_1 | rdh20 |  |  | 9018 | 0.366 | 0.2046 | No |
| 619 | 572849 | suv420h1 |  |  | 9019 | 0.366 | 0.2049 | No |
| 620 | 402954 | cbr4 |  |  | 9038 | 0.365 | 0.2045 | No |
| 621 | 100332635 | si:ch73-334d15.1 |  |  | 9056 | 0.364 | 0.2042 | No |
| 622 | 140744 | ckbb |  |  | 9069 | 0.363 | 0.2041 | No |
| 623 | 100330915 | si:dkey-85n7.8 |  |  | 9079 | 0.361 | 0.2040 | No |
| 624 | 562292\_1 | acsf3 |  |  | 9095 | 0.360 | 0.2038 | No |
| 625 | 558940 | sacm1la |  |  | 9115 | 0.359 | 0.2034 | No |
| 626 | 568381\_1 | smpd3 |  |  | 9129 | 0.358 | 0.2032 | No |
| 627 | 553433 | pisd |  |  | 9132 | 0.358 | 0.2034 | No |
| 628 | 751733 | dcakd |  |  | 9138 | 0.357 | 0.2034 | No |
| 629 | 564852 | pfkfb2b |  |  | 9163 | 0.355 | 0.2029 | No |
| 630 | 406474 | adob |  |  | 9191 | 0.353 | 0.2022 | No |
| 631 | 110437932 | LOC110437932 |  |  | 9193 | 0.353 | 0.2024 | No |
| 632 | 553513 | idi1 |  |  | 9202 | 0.353 | 0.2024 | No |
| 633 | 100332843 | mthfs |  |  | 9209 | 0.353 | 0.2024 | No |
| 634 | 570248 | galnt2 |  |  | 9235 | 0.351 | 0.2018 | No |
| 635 | 368229 | pmm2 |  |  | 9241 | 0.350 | 0.2019 | No |
| 636 | 678634\_1 | mat2al |  |  | 9250 | 0.350 | 0.2019 | No |
| 637 | 402827 | hao1 |  |  | 9271 | 0.348 | 0.2015 | No |
| 638 | 557680 | plpp3 |  |  | 9279 | 0.348 | 0.2015 | No |
| 639 | 503707 | upp1 |  |  | 9292 | 0.347 | 0.2013 | No |
| 640 | 557735 | urad |  |  | 9307 | 0.346 | 0.2011 | No |
| 641 | 571388 | hmgcll1 |  |  | 9312 | 0.346 | 0.2012 | No |
| 642 | 570459 | ndst1b |  |  | 9314 | 0.346 | 0.2014 | No |
| 643 | 378847 | mtr |  |  | 9320 | 0.346 | 0.2015 | No |
| 644 | 101886447 | LOC101886447 |  |  | 9321 | 0.346 | 0.2017 | No |
| 645 | 393469 | gmppab |  |  | 9329 | 0.345 | 0.2017 | No |
| 646 | 557756\_2 | enpp7.1 |  |  | 9331 | 0.345 | 0.2019 | No |
| 647 | 373866 | cbr1 |  |  | 9333 | 0.345 | 0.2021 | No |
| 648 | 445069 | ntpcr |  |  | 9362 | 0.343 | 0.2014 | No |
| 649 | 402985\_1 | grhprb |  |  | 9363 | 0.343 | 0.2016 | No |
| 650 | 101886516 | prdm6 |  |  | 9385 | 0.342 | 0.2012 | No |
| 651 | 436647 | aldh6a1 |  |  | 9402 | 0.340 | 0.2009 | No |
| 652 | 550550 | mecr |  |  | 9422 | 0.339 | 0.2005 | No |
| 653 | 100037326\_1 | pnpla4 |  |  | 9458 | 0.337 | 0.1995 | No |
| 654 | 407663\_1 | si:ch211-107o10.3 |  |  | 9470 | 0.336 | 0.1994 | No |
| 655 | 559521 | b3galt1a |  |  | 9474 | 0.336 | 0.1995 | No |
| 656 | 101884388 | inpp5a |  |  | 9486 | 0.335 | 0.1994 | No |
| 657 | 406408 | ddost |  |  | 9487 | 0.335 | 0.1996 | No |
| 658 | 552997 | fdps |  |  | 9497 | 0.335 | 0.1995 | No |
| 659 | 555376 | btd |  |  | 9519 | 0.332 | 0.1991 | No |
| 660 | 571825 | adcy3a |  |  | 9521 | 0.332 | 0.1993 | No |
| 661 | 336473\_1 | glulb |  |  | 9542 | 0.330 | 0.1988 | No |
| 662 | 793609 | hdc |  |  | 9608 | 0.327 | 0.1969 | No |
| 663 | 431754 | acaa1 |  |  | 9613 | 0.326 | 0.1969 | No |
| 664 | 393649 | mgat1a |  |  | 9629 | 0.325 | 0.1967 | No |
| 665 | 406558 | cant1a |  |  | 9674 | 0.323 | 0.1954 | No |
| 666 | 492760 | b4galt4 |  |  | 9718 | 0.320 | 0.1942 | No |
| 667 | 393400 | zgc:66313 |  |  | 9758 | 0.318 | 0.1931 | No |
| 668 | 352926 | gpx1a |  |  | 9766 | 0.318 | 0.1931 | No |
| 669 | 561542\_1 | si:dkey-23o4.6 |  |  | 9769 | 0.317 | 0.1932 | No |
| 670 | 393549 | asah1b |  |  | 9770 | 0.317 | 0.1934 | No |
| 671 | 393539 | dhrs4 |  |  | 9778 | 0.317 | 0.1934 | No |
| 672 | 140533 | ND3 |  |  | 9782 | 0.317 | 0.1936 | No |
| 673 | 571011 | pde8b |  |  | 9805 | 0.316 | 0.1930 | No |
| 674 | 568381 | smpd3 |  |  | 9809 | 0.315 | 0.1931 | No |
| 675 | 322513 | uap1 |  |  | 9831 | 0.314 | 0.1927 | No |
| 676 | 796749 | si:ch73-54n14.2 |  |  | 9859 | 0.312 | 0.1920 | No |
| 677 | 559107 | LOC559107 |  |  | 9862 | 0.312 | 0.1921 | No |
| 678 | 108179934 | nnt2 |  |  | 9874 | 0.311 | 0.1920 | No |
| 679 | 449784\_1 | mgst1.1 |  |  | 9903 | 0.310 | 0.1912 | No |
| 680 | 431724 | impdh1a |  |  | 9905 | 0.310 | 0.1914 | No |
| 681 | 352928 | gpx4a |  |  | 9915 | 0.309 | 0.1913 | No |
| 682 | 335817 | pkma |  |  | 9917 | 0.309 | 0.1915 | No |
| 683 | 405766 | b3gnt2a |  |  | 9926 | 0.308 | 0.1914 | No |
| 684 | 562771 | nadkb |  |  | 9927 | 0.308 | 0.1917 | No |
| 685 | 100002145 | si:ch73-337l15.2 |  |  | 9937 | 0.307 | 0.1916 | No |
| 686 | 793735 | gal3st1a |  |  | 9966 | 0.305 | 0.1908 | No |
| 687 | 322660\_1 | upb1 |  |  | 9992 | 0.304 | 0.1902 | No |
| 688 | 322468 | chs1 |  |  | 10005 | 0.303 | 0.1900 | No |
| 689 | 555969 | lpcat1 |  |  | 10011 | 0.302 | 0.1901 | No |
| 690 | 100148292 | npl |  |  | 10026 | 0.302 | 0.1898 | No |
| 691 | 378713 | tktb |  |  | 10030 | 0.301 | 0.1899 | No |
| 692 | 100002956 | si:ch211-195e19.1 |  |  | 10032 | 0.301 | 0.1901 | No |
| 693 | 503760 | poc1bl |  |  | 10050 | 0.300 | 0.1897 | No |
| 694 | 100148642 | pigv |  |  | 10057 | 0.300 | 0.1897 | No |
| 695 | 555911 | npr1b |  |  | 10076 | 0.298 | 0.1893 | No |
| 696 | 793834 | hadhab |  |  | 10077 | 0.298 | 0.1895 | No |
| 697 | 100329827 | gad1a |  |  | 10096 | 0.296 | 0.1891 | No |
| 698 | 100415791 | ugt5b1 |  |  | 10103 | 0.296 | 0.1891 | No |
| 699 | novel.327 | - |  |  | 10107 | 0.295 | 0.1892 | No |
| 700 | 100148744 | itpk1b |  |  | 10111 | 0.295 | 0.1893 | No |
| 701 | 692278 | mboat2b |  |  | 10139 | 0.294 | 0.1886 | No |
| 702 | 503885 | fut9d |  |  | 10168 | 0.292 | 0.1879 | No |
| 703 | 405845 | zgc:77118 |  |  | 10210 | 0.290 | 0.1867 | No |
| 704 | 100034366 | cyp2p8 |  |  | 10215 | 0.290 | 0.1868 | No |
| 705 | 569769\_1 | pomt1 |  |  | 10230 | 0.289 | 0.1865 | No |
| 706 | 100006345 | fktn |  |  | 10231 | 0.289 | 0.1867 | No |
| 707 | 565971 | etnk1 |  |  | 10254 | 0.288 | 0.1862 | No |
| 708 | 406298\_1 | ndufa4 |  |  | 10280 | 0.286 | 0.1855 | No |
| 709 | 100008541\_1 | tdo2a |  |  | 10281 | 0.286 | 0.1857 | No |
| 710 | 553501 | lratb |  |  | 10289 | 0.285 | 0.1857 | No |
| 711 | 393387 | ggt1a |  |  | 10290 | 0.285 | 0.1859 | No |
| 712 | 567720\_2 | idua |  |  | 10293 | 0.285 | 0.1860 | No |
| 713 | 322341 | cyp27a1.4 |  |  | 10296 | 0.285 | 0.1861 | No |
| 714 | 368360 | lbr |  |  | 10303 | 0.285 | 0.1861 | No |
| 715 | 767715 | srd5a1 |  |  | 10315 | 0.284 | 0.1860 | No |
| 716 | 393105 | hsd17b4 |  |  | 10332 | 0.283 | 0.1856 | No |
| 717 | 394247 | pafah1b1b |  |  | 10333 | 0.283 | 0.1858 | No |
| 718 | 394033 | pafah1b3 |  |  | 10341 | 0.282 | 0.1858 | No |
| 719 | 393838 | mtmr3 |  |  | 10410 | 0.278 | 0.1837 | No |
| 720 | 406323 | chia.1 |  |  | 10449 | 0.276 | 0.1826 | No |
| 721 | 569793 | ndufa9b |  |  | 10481 | 0.274 | 0.1817 | No |
| 722 | 446214 | gyltl1b |  |  | 10516 | 0.271 | 0.1808 | No |
| 723 | 322609 | st6galnac3 |  |  | 10527 | 0.271 | 0.1806 | No |
| 724 | 406789\_1 | pank1a |  |  | 10534 | 0.271 | 0.1806 | No |
| 725 | 562573 | hpse2 |  |  | 10567 | 0.268 | 0.1797 | No |
| 726 | 322533 | anpepb |  |  | 10588 | 0.267 | 0.1792 | No |
| 727 | 406739 | pla2g12b |  |  | 10652 | 0.263 | 0.1773 | No |
| 728 | 100334784 | galnt5 |  |  | 10675 | 0.262 | 0.1767 | No |
| 729 | 541489 | acp1 |  |  | 10684 | 0.261 | 0.1767 | No |
| 730 | 569930\_1 | zgc:153896 |  |  | 10686 | 0.261 | 0.1768 | No |
| 731 | 795261 | ugt1b7 |  |  | 10698 | 0.261 | 0.1766 | No |
| 732 | 436919 | ada |  |  | 10721 | 0.259 | 0.1761 | No |
| 733 | 100003595 | ak5 |  |  | 10724 | 0.259 | 0.1762 | No |
| 734 | 793584 | gal3st1b |  |  | 10746 | 0.258 | 0.1756 | No |
| 735 | 101887155 | pnpla2 |  |  | 10760 | 0.257 | 0.1754 | No |
| 736 | 323020 | cers5 |  |  | 10762 | 0.257 | 0.1755 | No |
| 737 | 768196 | me1 |  |  | 10781 | 0.256 | 0.1751 | No |
| 738 | 101884921 | LOC101884921 |  |  | 10800 | 0.255 | 0.1747 | No |
| 739 | 405865 | ethe1 |  |  | 10812 | 0.254 | 0.1745 | No |
| 740 | 641488 | ugt1a1 |  |  | 10816 | 0.254 | 0.1746 | No |
| 741 | 557191 | gucy1b2 |  |  | 10854 | 0.252 | 0.1735 | No |
| 742 | 110438480 | LOC110438480 |  |  | 10875 | 0.250 | 0.1730 | No |
| 743 | 100000992 | si:ch211-235e9.6 |  |  | 10890 | 0.250 | 0.1727 | No |
| 744 | 393421 | etnppl |  |  | 10904 | 0.249 | 0.1724 | No |
| 745 | 393938 | fh |  |  | 10925 | 0.248 | 0.1719 | No |
| 746 | 563476 | cox7b |  |  | 10950 | 0.247 | 0.1713 | No |
| 747 | 492773\_1 | pomk |  |  | 10979 | 0.245 | 0.1705 | No |
| 748 | 497281 | ext1c |  |  | 10987 | 0.244 | 0.1704 | No |
| 749 | 100137124 | dgkh |  |  | 11010 | 0.243 | 0.1699 | No |
| 750 | 394060 | hmgcs1 |  |  | 11015 | 0.243 | 0.1699 | No |
| 751 | 100333538 | LOC100333538 |  |  | 11027 | 0.242 | 0.1697 | No |
| 752 | 474325 | dck |  |  | 11056 | 0.241 | 0.1689 | No |
| 753 | 560410 | si:ch211-132f19.7 |  |  | 11057 | 0.241 | 0.1691 | No |
| 754 | 30095 | ckma |  |  | 11102 | 0.239 | 0.1678 | No |
| 755 | 796449 | si:dkeyp-98a7.1 |  |  | 11113 | 0.238 | 0.1676 | No |
| 756 | 565669 | plcd4a |  |  | 11125 | 0.238 | 0.1674 | No |
| 757 | 407700 | plcb4 |  |  | 11151 | 0.236 | 0.1667 | No |
| 758 | 791728 | renbp |  |  | 11167 | 0.235 | 0.1664 | No |
| 759 | 406791\_2 | hk1 |  |  | 11184 | 0.234 | 0.1660 | No |
| 760 | 100534896 | pgs1 |  |  | 11187 | 0.234 | 0.1661 | No |
| 761 | 30430 | dnmt1 |  |  | 11188 | 0.234 | 0.1663 | No |
| 762 | 100149080 | LOC100149080 |  |  | 11197 | 0.233 | 0.1662 | No |
| 763 | 445322 | chst10 |  |  | 11199 | 0.233 | 0.1663 | No |
| 764 | 336798 | ggps1 |  |  | 11200 | 0.233 | 0.1664 | No |
| 765 | 563950 | si:dkey-221h15.4 |  |  | 11202 | 0.233 | 0.1666 | No |
| 766 | 550460 | hexa |  |  | 11206 | 0.233 | 0.1666 | No |
| 767 | 403086 | gpt2l |  |  | 11238 | 0.231 | 0.1657 | No |
| 768 | 406367 | gapdhs |  |  | 11242 | 0.231 | 0.1658 | No |
| 769 | 436600 | ebp |  |  | 11261 | 0.230 | 0.1654 | No |
| 770 | 321084 | dnmt3ba |  |  | 11265 | 0.230 | 0.1654 | No |
| 771 | 100003139 | tcirg1a |  |  | 11269 | 0.229 | 0.1655 | No |
| 772 | 442923\_1 | oxct1a |  |  | 11274 | 0.229 | 0.1655 | No |
| 773 | 327601 | alg8 |  |  | 11281 | 0.229 | 0.1655 | No |
| 774 | 559097 | cyp7a1b |  |  | 11302 | 0.227 | 0.1649 | No |
| 775 | 80939 | papss2b |  |  | 11321 | 0.226 | 0.1645 | No |
| 776 | 58120 | nme6 |  |  | 11325 | 0.226 | 0.1645 | No |
| 777 | 436958\_1 | gpat3 |  |  | 11354 | 0.224 | 0.1638 | No |
| 778 | 406703\_1 | gsta.1 |  |  | 11380 | 0.223 | 0.1631 | No |
| 779 | 768123 | b4galt1l |  |  | 11388 | 0.223 | 0.1630 | No |
| 780 | 325402 | atp6v0cb |  |  | 11397 | 0.222 | 0.1629 | No |
| 781 | 798788 | gpx3 |  |  | 11398 | 0.222 | 0.1630 | No |
| 782 | 259250 | cers4a |  |  | 11402 | 0.222 | 0.1631 | No |
| 783 | 100037356 | bdh1 |  |  | 11416 | 0.221 | 0.1628 | No |
| 784 | 447838 | coasy |  |  | 11435 | 0.220 | 0.1623 | No |
| 785 | 777793 | sult2st2 |  |  | 11440 | 0.220 | 0.1624 | No |
| 786 | 492779 | nansb |  |  | 11453 | 0.219 | 0.1621 | No |
| 787 | 555443 | plcd1a |  |  | 11457 | 0.219 | 0.1622 | No |
| 788 | 559656 | acsl3b |  |  | 11481 | 0.218 | 0.1615 | No |
| 789 | 792063 | ft2 |  |  | 11497 | 0.217 | 0.1612 | No |
| 790 | 403051\_1 | pygb |  |  | 11510 | 0.216 | 0.1609 | No |
| 791 | 563019 | itpkb |  |  | 11517 | 0.216 | 0.1609 | No |
| 792 | 266992 | cad |  |  | 11587 | 0.212 | 0.1587 | No |
| 793 | 100170813 | galnt18a |  |  | 11602 | 0.211 | 0.1584 | No |
| 794 | 794667 | otc |  |  | 11618 | 0.210 | 0.1580 | No |
| 795 | 140541 | COX3 |  |  | 11660 | 0.208 | 0.1568 | No |
| 796 | 562569 | plcd3b |  |  | 11668 | 0.208 | 0.1567 | No |
| 797 | 406259 | hprt1 |  |  | 11719 | 0.205 | 0.1552 | No |
| 798 | 556641 | pld1a |  |  | 11723 | 0.205 | 0.1552 | No |
| 799 | 436754 | gstz1 |  |  | 11729 | 0.204 | 0.1552 | No |
| 800 | 641489 | zgc:123305 |  |  | 11735 | 0.204 | 0.1551 | No |
| 801 | 100149838 | pycr1b |  |  | 11739 | 0.204 | 0.1552 | No |
| 802 | 565706\_2 | pde4ba |  |  | 11746 | 0.204 | 0.1551 | No |
| 803 | 793309 | cyp27b1 |  |  | 11749 | 0.203 | 0.1552 | No |
| 804 | 559295 | cecr1b |  |  | 11760 | 0.203 | 0.1550 | No |
| 805 | 327635 | gnsb |  |  | 11764 | 0.202 | 0.1550 | No |
| 806 | 100000654 | chpf2 |  |  | 11767 | 0.202 | 0.1551 | No |
| 807 | 565422 | LOC565422 |  |  | 11770 | 0.202 | 0.1552 | No |
| 808 | 30086\_1 | nme7 |  |  | 11771 | 0.202 | 0.1553 | No |
| 809 | 323329 | mat2aa |  |  | 11774 | 0.202 | 0.1554 | No |
| 810 | 406325 | acaa2 |  |  | 11783 | 0.202 | 0.1553 | No |
| 811 | 335538 | man1a2 |  |  | 11788 | 0.202 | 0.1553 | No |
| 812 | 554036 | cyp26c1 |  |  | 11800 | 0.201 | 0.1550 | No |
| 813 | 100000750 | g6pcb |  |  | 11811 | 0.200 | 0.1548 | No |
| 814 | 797512 | fut8a |  |  | 11822 | 0.200 | 0.1546 | No |
| 815 | novel.854 | - |  |  | 11827 | 0.200 | 0.1546 | No |
| 816 | 30617 | urod |  |  | 11835 | 0.199 | 0.1546 | No |
| 817 | 406734 | agpat3 |  |  | 11853 | 0.198 | 0.1541 | No |
| 818 | 796997 | ndufs7 |  |  | 11858 | 0.198 | 0.1541 | No |
| 819 | 560951 | xylt1 |  |  | 11871 | 0.197 | 0.1539 | No |
| 820 | 556575 | lratb.2 |  |  | 11876 | 0.197 | 0.1539 | No |
| 821 | 368415 | ptenb |  |  | 11887 | 0.196 | 0.1537 | No |
| 822 | 445122 | ndufa3 |  |  | 11919 | 0.195 | 0.1527 | No |
| 823 | 565876 | si:dkey-91i10.3 |  |  | 11920 | 0.195 | 0.1529 | No |
| 824 | 569112 | pde11al |  |  | 11925 | 0.195 | 0.1529 | No |
| 825 | 563038 | mgat4a |  |  | 11936 | 0.194 | 0.1527 | No |
| 826 | 560719 | si:dkey-206f10.1 |  |  | 11948 | 0.193 | 0.1524 | No |
| 827 | 406590 | ak4 |  |  | 11966 | 0.193 | 0.1520 | No |
| 828 | 402841\_1 | adh8b |  |  | 11968 | 0.193 | 0.1521 | No |
| 829 | 415207 | rbks |  |  | 11975 | 0.193 | 0.1520 | No |
| 830 | 777792 | sult2st3 |  |  | 12024 | 0.189 | 0.1505 | No |
| 831 | 100002993 | ugt5e1 |  |  | 12048 | 0.188 | 0.1499 | No |
| 832 | 393943 | ndufa6 |  |  | 12056 | 0.188 | 0.1498 | No |
| 833 | 437019 | pcca |  |  | 12058 | 0.187 | 0.1499 | No |
| 834 | 100001225 | st6galnac2 |  |  | 12066 | 0.187 | 0.1498 | No |
| 835 | 414331 | cyp51 |  |  | 12077 | 0.186 | 0.1496 | No |
| 836 | 641479 | ugt5a1 |  |  | 12114 | 0.184 | 0.1485 | No |
| 837 | 100126019 | zgc:171967 |  |  | 12121 | 0.184 | 0.1484 | No |
| 838 | 337599\_1 | zgc:172121 |  |  | 12142 | 0.182 | 0.1479 | No |
| 839 | 436878 | sirt5 |  |  | 12147 | 0.182 | 0.1479 | No |
| 840 | 324407 | chsy1 |  |  | 12166 | 0.181 | 0.1474 | No |
| 841 | 558898 | si:rp71-1c23.3 |  |  | 12189 | 0.180 | 0.1468 | No |
| 842 | 100332039 | enpp2 |  |  | 12204 | 0.179 | 0.1464 | No |
| 843 | 140424 | gucy2f |  |  | 12215 | 0.179 | 0.1462 | No |
| 844 | 393912 | b4galt6 |  |  | 12226 | 0.178 | 0.1460 | No |
| 845 | 100150288 | elovl6l |  |  | 12263 | 0.176 | 0.1449 | No |
| 846 | 266797 | stt3a |  |  | 12268 | 0.176 | 0.1449 | No |
| 847 | 567913 | mogs |  |  | 12273 | 0.175 | 0.1449 | No |
| 848 | 550233 | ca8 |  |  | 12295 | 0.174 | 0.1443 | No |
| 849 | 568162 | kmt2cb |  |  | 12302 | 0.174 | 0.1442 | No |
| 850 | 100148835 | dgkzb |  |  | 12306 | 0.174 | 0.1442 | No |
| 851 | 568616 | smyd2b |  |  | 12307 | 0.173 | 0.1444 | No |
| 852 | 562592\_1 | man1b1a |  |  | 12355 | 0.171 | 0.1429 | No |
| 853 | 323613 | hexb |  |  | 12362 | 0.171 | 0.1428 | No |
| 854 | 406421 | acox3 |  |  | 12372 | 0.170 | 0.1426 | No |
| 855 | 322626 | hsd17b12b |  |  | 12386 | 0.170 | 0.1423 | No |
| 856 | 541435\_1 | acss1 |  |  | 12397 | 0.169 | 0.1421 | No |
| 857 | 321724 | atp6v0b |  |  | 12410 | 0.169 | 0.1418 | No |
| 858 | 567592 | smpd5 |  |  | 12443 | 0.167 | 0.1408 | No |
| 859 | 564147 | glsa |  |  | 12468 | 0.166 | 0.1401 | No |
| 860 | 406339 | hk2 |  |  | 12475 | 0.165 | 0.1401 | No |
| 861 | 436610 | pnpla3 |  |  | 12510 | 0.164 | 0.1390 | No |
| 862 | 393714 | cdo1 |  |  | 12520 | 0.163 | 0.1388 | No |
| 863 | 541538 | zgc:110540 |  |  | 12538 | 0.162 | 0.1384 | No |
| 864 | 571355 | ampd2b |  |  | 12561 | 0.162 | 0.1377 | No |
| 865 | 494154 | lancl1 |  |  | 12582 | 0.160 | 0.1372 | No |
| 866 | 562774 | p4ha1a |  |  | 12599 | 0.160 | 0.1368 | No |
| 867 | 100002035 | acsm3 |  |  | 12623 | 0.158 | 0.1361 | No |
| 868 | 393584 | mtmr4 |  |  | 12629 | 0.158 | 0.1360 | No |
| 869 | 560219 | man2a1 |  |  | 12647 | 0.157 | 0.1356 | No |
| 870 | 557834 | itpa |  |  | 12663 | 0.156 | 0.1352 | No |
| 871 | 553717 | vkorc1l1 |  |  | 12664 | 0.156 | 0.1353 | No |
| 872 | 566085 | tecra |  |  | 12679 | 0.155 | 0.1349 | No |
| 873 | 566863 | acsl6 |  |  | 12701 | 0.154 | 0.1343 | No |
| 874 | 322228 | bhmt |  |  | 12711 | 0.154 | 0.1341 | No |
| 875 | 570437 | pfas |  |  | 12713 | 0.154 | 0.1342 | No |
| 876 | 613246 | mpi |  |  | 12717 | 0.153 | 0.1342 | No |
| 877 | 449831 | pip5k1ba |  |  | 12759 | 0.151 | 0.1329 | No |
| 878 | 30390 | cyp19a1a |  |  | 12768 | 0.151 | 0.1328 | No |
| 879 | 335985 | rpn2 |  |  | 12774 | 0.151 | 0.1327 | No |
| 880 | 415216 | guk1a |  |  | 12813 | 0.149 | 0.1315 | No |
| 881 | 403116 | st6gal2a |  |  | 12869 | 0.147 | 0.1298 | No |
| 882 | 100034522\_1 | mthfd1l |  |  | 12875 | 0.147 | 0.1297 | No |
| 883 | 450078 | hsd17b10 |  |  | 12879 | 0.146 | 0.1297 | No |
| 884 | 431762 | mgst1.2 |  |  | 12896 | 0.146 | 0.1293 | No |
| 885 | 449649 | galca |  |  | 12897 | 0.146 | 0.1294 | No |
| 886 | 566398 | adpgk2 |  |  | 12909 | 0.145 | 0.1291 | No |
| 887 | 192309 | tpi1a |  |  | 12926 | 0.144 | 0.1287 | No |
| 888 | 100004652 | si:ch211-81n22.1 |  |  | 13002 | 0.140 | 0.1262 | No |
| 889 | 556229 | aass |  |  | 13019 | 0.139 | 0.1258 | No |
| 890 | 791142 | sacm1lb |  |  | 13025 | 0.139 | 0.1257 | No |
| 891 | 798876 | pip5k1ca |  |  | 13026 | 0.139 | 0.1258 | No |
| 892 | 562919 | itpkca |  |  | 13039 | 0.138 | 0.1255 | No |
| 893 | 324556 | taldo1 |  |  | 13048 | 0.137 | 0.1253 | No |
| 894 | 393787 | pcbd1 |  |  | 13059 | 0.137 | 0.1251 | No |
| 895 | 553646 | sptlc2a |  |  | 13078 | 0.136 | 0.1246 | No |
| 896 | 559073 | galnt1 |  |  | 13107 | 0.135 | 0.1237 | No |
| 897 | 791730 | got1l1 |  |  | 13108 | 0.135 | 0.1238 | No |
| 898 | 497279 | ext1a |  |  | 13116 | 0.135 | 0.1237 | No |
| 899 | 553668 | galnt7 |  |  | 13122 | 0.134 | 0.1236 | No |
| 900 | 436989 | allc |  |  | 13137 | 0.134 | 0.1232 | No |
| 901 | 406725 | elovl1b |  |  | 13166 | 0.132 | 0.1224 | No |
| 902 | 436893\_1 | coq3 |  |  | 13197 | 0.130 | 0.1215 | No |
| 903 | 140536 | ND6 |  |  | 13213 | 0.130 | 0.1210 | No |
| 904 | 100332911 | gpam |  |  | 13236 | 0.129 | 0.1204 | No |
| 905 | 563849 | sgsh |  |  | 13237 | 0.129 | 0.1205 | No |
| 906 | 403338 | gnmt |  |  | 13238 | 0.129 | 0.1206 | No |
| 907 | 541539 | alpi.1 |  |  | 13250 | 0.128 | 0.1203 | No |
| 908 | 100192219 | gch1 |  |  | 13304 | 0.125 | 0.1186 | No |
| 909 | 101882140 | LOC101882140 |  |  | 13313 | 0.125 | 0.1184 | No |
| 910 | 327417 | hsd17b12a |  |  | 13341 | 0.124 | 0.1176 | No |
| 911 | 100126133 | ppt2 |  |  | 13345 | 0.123 | 0.1176 | No |
| 912 | 560006 | inpp4b |  |  | 13364 | 0.122 | 0.1170 | No |
| 913 | 767682 | zgc:152830 |  |  | 13374 | 0.122 | 0.1168 | No |
| 914 | 568096 | olah |  |  | 13379 | 0.122 | 0.1168 | No |
| 915 | 791159 | galns |  |  | 13383 | 0.122 | 0.1168 | No |
| 916 | 447889 | pnp5b |  |  | 13386 | 0.121 | 0.1168 | No |
| 917 | 101883911 | LOC101883911 |  |  | 13424 | 0.119 | 0.1156 | No |
| 918 | 100002366 | gaa |  |  | 13446 | 0.118 | 0.1150 | No |
| 919 | 406265 | agpat4 |  |  | 13450 | 0.118 | 0.1150 | No |
| 920 | 140531 | ND1 |  |  | 13453 | 0.118 | 0.1150 | No |
| 921 | 563972\_1 | gstt1a |  |  | 13477 | 0.117 | 0.1143 | No |
| 922 | 562037 | eprs |  |  | 13491 | 0.116 | 0.1139 | No |
| 923 | 393096 | lipg |  |  | 13509 | 0.115 | 0.1134 | No |
| 924 | 337412 | bcat1 |  |  | 13510 | 0.115 | 0.1135 | No |
| 925 | 30497 | ldhba |  |  | 13522 | 0.114 | 0.1132 | No |
| 926 | 100004770\_2 | setd8b |  |  | 13531 | 0.114 | 0.1130 | No |
| 927 | 406567 | fggy |  |  | 13547 | 0.113 | 0.1126 | No |
| 928 | 445042 | ndufa7 |  |  | 13581 | 0.112 | 0.1116 | No |
| 929 | 799387 | galnt16 |  |  | 13641 | 0.109 | 0.1097 | No |
| 930 | 100004770\_1 | setd8b |  |  | 13643 | 0.109 | 0.1097 | No |
| 931 | 100000709\_1 | whsc1 |  |  | 13661 | 0.108 | 0.1092 | No |
| 932 | 140532 | ND2 |  |  | 13672 | 0.108 | 0.1090 | No |
| 933 | 317743 | gapdh |  |  | 13695 | 0.107 | 0.1083 | No |
| 934 | 566527 | dad1 |  |  | 13709 | 0.106 | 0.1079 | No |
| 935 | 553792 | fpgt |  |  | 13722 | 0.106 | 0.1076 | No |
| 936 | 336526 | b3gnt5a |  |  | 13729 | 0.106 | 0.1075 | No |
| 937 | 557902 | adcy2b |  |  | 13739 | 0.105 | 0.1072 | No |
| 938 | 246094 | gpia |  |  | 13750 | 0.104 | 0.1070 | No |
| 939 | 436949 | bcat2 |  |  | 13764 | 0.103 | 0.1066 | No |
| 940 | 108179658 | LOC108179658 |  |  | 13784 | 0.102 | 0.1060 | No |
| 941 | 796549 | chst8 |  |  | 13786 | 0.102 | 0.1061 | No |
| 942 | 796295 | dpm2 |  |  | 13795 | 0.102 | 0.1059 | No |
| 943 | 492759 | cyp3c3 |  |  | 13798 | 0.102 | 0.1059 | No |
| 944 | 100000478 | mgat2 |  |  | 13808 | 0.101 | 0.1056 | No |
| 945 | 793635 | b4galnt1b |  |  | 13834 | 0.100 | 0.1049 | No |
| 946 | 565743 | pld2 |  |  | 13902 | 0.096 | 0.1027 | No |
| 947 | 393821 | ndufb7 |  |  | 13910 | 0.096 | 0.1025 | No |
| 948 | 394161 | cds2 |  |  | 13917 | 0.096 | 0.1024 | No |
| 949 | 30740 | rrm1 |  |  | 13918 | 0.096 | 0.1024 | No |
| 950 | 322309 | sirt2 |  |  | 13923 | 0.096 | 0.1024 | No |
| 951 | 570474 | urahb |  |  | 13930 | 0.095 | 0.1022 | No |
| 952 | 559373 | galnt8b.1 |  |  | 13937 | 0.095 | 0.1021 | No |
| 953 | 393326 | st3gal3a |  |  | 13966 | 0.094 | 0.1012 | No |
| 954 | 767664\_1 | cox7a1 |  |  | 13979 | 0.094 | 0.1009 | No |
| 955 | 567970 | setd1ba |  |  | 14027 | 0.091 | 0.0994 | No |
| 956 | 406330\_1 | got1 |  |  | 14060 | 0.089 | 0.0984 | No |
| 957 | 100330160 | nampta |  |  | 14078 | 0.089 | 0.0978 | No |
| 958 | 100007670 | glceb |  |  | 14087 | 0.088 | 0.0976 | No |
| 959 | 192305 | adssl |  |  | 14103 | 0.087 | 0.0972 | No |
| 960 | 556237\_2 | man1b1b |  |  | 14114 | 0.087 | 0.0969 | No |
| 961 | 494083 | mogat3b |  |  | 14136 | 0.085 | 0.0963 | No |
| 962 | 492710 | aldh16a1 |  |  | 14167 | 0.084 | 0.0953 | No |
| 963 | 557186 | aldh18a1 |  |  | 14170 | 0.084 | 0.0953 | No |
| 964 | 572052 | gucy2c |  |  | 14191 | 0.083 | 0.0947 | No |
| 965 | 100333987 | man2a2 |  |  | 14195 | 0.083 | 0.0946 | No |
| 966 | 407659 | st3gal3b |  |  | 14198 | 0.082 | 0.0946 | No |
| 967 | 100331745 | npr2 |  |  | 14217 | 0.081 | 0.0941 | No |
| 968 | 751596\_1 | cryl1 |  |  | 14220 | 0.081 | 0.0941 | No |
| 969 | 259251 | cers2a |  |  | 14222 | 0.081 | 0.0941 | No |
| 970 | 322420 | chia.6 |  |  | 14224 | 0.081 | 0.0941 | No |
| 971 | 394033\_1 | pafah1b3 |  |  | 14245 | 0.081 | 0.0935 | No |
| 972 | 493780 | ext2 |  |  | 14246 | 0.080 | 0.0936 | No |
| 973 | 559207 | ogdhl |  |  | 14255 | 0.080 | 0.0933 | No |
| 974 | 327506 | ckmt2a |  |  | 14267 | 0.080 | 0.0930 | No |
| 975 | 777624 | agpat2 |  |  | 14299 | 0.078 | 0.0920 | No |
| 976 | 570613 | sord |  |  | 14326 | 0.077 | 0.0912 | No |
| 977 | 553725 | alg3 |  |  | 14327 | 0.077 | 0.0913 | No |
| 978 | 140535 | ND5 |  |  | 14336 | 0.077 | 0.0911 | No |
| 979 | 557026 | adcy1a |  |  | 14359 | 0.076 | 0.0904 | No |
| 980 | 792097\_1 | amd1 |  |  | 14362 | 0.076 | 0.0904 | No |
| 981 | 378960 | mgll |  |  | 14379 | 0.075 | 0.0899 | No |
| 982 | 406791 | hk1 |  |  | 14413 | 0.073 | 0.0888 | No |
| 983 | 566825 | kmt2bb |  |  | 14417 | 0.073 | 0.0888 | No |
| 984 | 567881\_1 | sat1b |  |  | 14419 | 0.073 | 0.0888 | No |
| 985 | 100151215 | pde7a |  |  | 14433 | 0.073 | 0.0884 | No |
| 986 | 571876\_1 | pomgnt1 |  |  | 14443 | 0.072 | 0.0881 | No |
| 987 | 405822 | pgm2 |  |  | 14455 | 0.072 | 0.0878 | No |
| 988 | 445168 | sptlc3 |  |  | 14458 | 0.071 | 0.0878 | No |
| 989 | 553452 | si:dkey-180p18.9 |  |  | 14479 | 0.070 | 0.0872 | No |
| 990 | 393461 | gmds |  |  | 14496 | 0.069 | 0.0867 | No |
| 991 | 100126013 | nagk |  |  | 14498 | 0.069 | 0.0867 | No |
| 992 | 557286 | sptlc2b |  |  | 14508 | 0.069 | 0.0864 | No |
| 993 | 100001532 | plcb3 |  |  | 14510 | 0.069 | 0.0865 | No |
| 994 | 323929 | mtmr14 |  |  | 14552 | 0.067 | 0.0851 | No |
| 995 | 100004977\_1 | mthfd2 |  |  | 14563 | 0.067 | 0.0848 | No |
| 996 | 373082 | gys2 |  |  | 14567 | 0.066 | 0.0848 | No |
| 997 | 751629 | setd8a |  |  | 14607 | 0.065 | 0.0835 | No |
| 998 | 558716 | galnt11 |  |  | 14624 | 0.064 | 0.0830 | No |
| 999 | 352933 | sps2 |  |  | 14627 | 0.064 | 0.0830 | No |
| 1000 | 100048920 | thtpa |  |  | 14628 | 0.064 | 0.0830 | No |
| 1001 | 100332380 | cers3a |  |  | 14629 | 0.064 | 0.0831 | No |
| 1002 | 100000709 | whsc1 |  |  | 14686 | 0.061 | 0.0812 | No |
| 1003 | 402981 | amdhd2 |  |  | 14698 | 0.060 | 0.0809 | No |
| 1004 | 560856 | kmt2ba |  |  | 14703 | 0.060 | 0.0808 | No |
| 1005 | 556781 | tdh2 |  |  | 14737 | 0.059 | 0.0797 | No |
| 1006 | 100003675 | p4ha1b |  |  | 14764 | 0.057 | 0.0789 | No |
| 1007 | 335153 | setdb2 |  |  | 14765 | 0.057 | 0.0790 | No |
| 1008 | 81540 | tyms |  |  | 14768 | 0.057 | 0.0789 | No |
| 1009 | 101669768\_1 | b4gat1 |  |  | 14770 | 0.057 | 0.0789 | No |
| 1010 | 795008 | si:ch211-269c21.2 |  |  | 14784 | 0.056 | 0.0785 | No |
| 1011 | 559659 | atp5ia |  |  | 14787 | 0.056 | 0.0785 | No |
| 1012 | 406667 | pi4k2a |  |  | 14789 | 0.056 | 0.0785 | No |
| 1013 | 100384891 | ugt1a7 |  |  | 14811 | 0.055 | 0.0778 | No |
| 1014 | 30331 | cahz |  |  | 14822 | 0.055 | 0.0775 | No |
| 1015 | 553187 | dnmt3aa |  |  | 14825 | 0.054 | 0.0775 | No |
| 1016 | 100147850 | pgap1 |  |  | 14842 | 0.054 | 0.0770 | No |
| 1017 | 100536353 | ganc |  |  | 14851 | 0.053 | 0.0768 | No |
| 1018 | 405863 | mccc2 |  |  | 14859 | 0.053 | 0.0766 | No |
| 1019 | 406413 | ndufs8a |  |  | 14884 | 0.052 | 0.0758 | No |
| 1020 | 405806 | hoga1 |  |  | 14925 | 0.050 | 0.0745 | No |
| 1021 | 553380 | dgkza |  |  | 14928 | 0.050 | 0.0745 | No |
| 1022 | 541449\_1 | zgc:113516 |  |  | 14941 | 0.049 | 0.0741 | No |
| 1023 | 560360 | pip4k2aa |  |  | 14945 | 0.049 | 0.0740 | No |
| 1024 | 393857 | gne |  |  | 14951 | 0.049 | 0.0739 | No |
| 1025 | 100000684 | neu3.5 |  |  | 14952 | 0.049 | 0.0739 | No |
| 1026 | 407715 | st6galnac6 |  |  | 14966 | 0.048 | 0.0735 | No |
| 1027 | 450084 | gss |  |  | 14972 | 0.048 | 0.0734 | No |
| 1028 | 450031 | tmem189 |  |  | 14992 | 0.047 | 0.0728 | No |
| 1029 | 560793 | kmt2ca |  |  | 15000 | 0.046 | 0.0726 | No |
| 1030 | 799805\_1 | akr1a1b |  |  | 15001 | 0.046 | 0.0726 | No |
| 1031 | 541388\_1 | dbt |  |  | 15004 | 0.046 | 0.0726 | No |
| 1032 | 557972 | nt5c2b |  |  | 15006 | 0.046 | 0.0726 | No |
| 1033 | 794088\_1 | ptena |  |  | 15035 | 0.044 | 0.0717 | No |
| 1034 | 561469\_1 | atp6v0a2a |  |  | 15040 | 0.044 | 0.0716 | No |
| 1035 | 325179 | inppl1a |  |  | 15054 | 0.043 | 0.0711 | No |
| 1036 | 393598 | alg9 |  |  | 15056 | 0.043 | 0.0711 | No |
| 1037 | 324845 | nt5c2a |  |  | 15095 | 0.041 | 0.0699 | No |
| 1038 | 100331840 | sphk1 |  |  | 15103 | 0.041 | 0.0697 | No |
| 1039 | 140539 | COX1 |  |  | 15120 | 0.041 | 0.0692 | No |
| 1040 | 393393 | adprm |  |  | 15151 | 0.039 | 0.0682 | No |
| 1041 | 794598 | LOC794598 |  |  | 15191 | 0.038 | 0.0669 | No |
| 1042 | 378962 | pah |  |  | 15203 | 0.037 | 0.0666 | No |
| 1043 | 565862 | colgalt1 |  |  | 15218 | 0.036 | 0.0661 | No |
| 1044 | 557125 | si:dkey-103i16.6 |  |  | 15219 | 0.036 | 0.0661 | No |
| 1045 | 100150463\_1 | dgke |  |  | 15224 | 0.036 | 0.0660 | No |
| 1046 | 570443 | pigx |  |  | 15231 | 0.036 | 0.0658 | No |
| 1047 | 322339 | cs |  |  | 15243 | 0.035 | 0.0655 | No |
| 1048 | 557919\_1 | adpgk |  |  | 15264 | 0.034 | 0.0649 | No |
| 1049 | 564754 | nat8l |  |  | 15288 | 0.033 | 0.0641 | No |
| 1050 | 567728 | LOC567728 |  |  | 15292 | 0.033 | 0.0640 | No |
| 1051 | 557525 | si:dkey-239i20.2 |  |  | 15293 | 0.033 | 0.0640 | No |
| 1052 | 323994 | pigc |  |  | 15299 | 0.032 | 0.0639 | No |
| 1053 | 798206\_1 | zgc:194551 |  |  | 15303 | 0.032 | 0.0638 | No |
| 1054 | 556291 | gnpda2 |  |  | 15306 | 0.032 | 0.0638 | No |
| 1055 | 393845 | pde6c |  |  | 15307 | 0.032 | 0.0638 | No |
| 1056 | 566563 | sgms1 |  |  | 15313 | 0.032 | 0.0637 | No |
| 1057 | 334208 | ldhd |  |  | 15325 | 0.031 | 0.0633 | No |
| 1058 | 799638 | phospho2 |  |  | 15331 | 0.031 | 0.0632 | No |
| 1059 | 393697 | guk1b |  |  | 15337 | 0.031 | 0.0630 | No |
| 1060 | 559001 | fasn |  |  | 15355 | 0.030 | 0.0625 | No |
| 1061 | 100330698 | sgpp2 |  |  | 15386 | 0.028 | 0.0615 | No |
| 1062 | 492500 | gsto2 |  |  | 15402 | 0.027 | 0.0610 | No |
| 1063 | 394133 | aldh4a1 |  |  | 15420 | 0.026 | 0.0604 | No |
| 1064 | 406299 | sucla2 |  |  | 15431 | 0.025 | 0.0601 | No |
| 1065 | 664696 | tigara |  |  | 15440 | 0.025 | 0.0599 | No |
| 1066 | 394156 | acadl |  |  | 15452 | 0.024 | 0.0595 | No |
| 1067 | 563654 | nos2b |  |  | 15462 | 0.024 | 0.0592 | No |
| 1068 | 570457 | aox6 |  |  | 15519 | 0.021 | 0.0573 | No |
| 1069 | 447928 | uck1 |  |  | 15524 | 0.021 | 0.0572 | No |
| 1070 | 563343 | prune |  |  | 15533 | 0.020 | 0.0570 | No |
| 1071 | 567204 | alox5a |  |  | 15555 | 0.019 | 0.0563 | No |
| 1072 | 445202 | dpm1 |  |  | 15562 | 0.019 | 0.0561 | No |
| 1073 | 559442 | ptdss1b |  |  | 15571 | 0.019 | 0.0558 | No |
| 1074 | 415161 | sirt6 |  |  | 15578 | 0.018 | 0.0556 | No |
| 1075 | 560826 | csgalnact2 |  |  | 15583 | 0.018 | 0.0555 | No |
| 1076 | 436652 | entpd8 |  |  | 15584 | 0.018 | 0.0555 | No |
| 1077 | 393761 | rdh8b |  |  | 15593 | 0.018 | 0.0553 | No |
| 1078 | 799942 | pde9a |  |  | 15616 | 0.017 | 0.0545 | No |
| 1079 | 260441 | mthfd1b |  |  | 15643 | 0.015 | 0.0537 | No |
| 1080 | 394170 | nme4 |  |  | 15678 | 0.013 | 0.0525 | No |
| 1081 | 100536523 | kmt2d |  |  | 15707 | 0.011 | 0.0516 | No |
| 1082 | 541317\_2 | gatb |  |  | 15725 | 0.010 | 0.0510 | No |
| 1083 | 562714 | nfs1 |  |  | 15741 | 0.009 | 0.0505 | No |
| 1084 | 140540 | COX2 |  |  | 15750 | 0.009 | 0.0503 | No |
| 1085 | 561469 | atp6v0a2a |  |  | 15771 | 0.008 | 0.0496 | No |
| 1086 | 569980 | bckdhb |  |  | 15788 | 0.007 | 0.0491 | No |
| 1087 | 797748 | glsl |  |  | 15795 | 0.007 | 0.0489 | No |
| 1088 | 386710 | mibp2 |  |  | 15802 | 0.006 | 0.0487 | No |
| 1089 | 406306 | ndufb4 |  |  | 15811 | 0.006 | 0.0484 | No |
| 1090 | 557240 | ggcx |  |  | 15839 | 0.005 | 0.0475 | No |
| 1091 | 565059 | pank1b |  |  | 15865 | 0.004 | 0.0467 | No |
| 1092 | 282672\_1 | fbp1b |  |  | 15912 | 0.002 | 0.0451 | No |
| 1093 | 791592 | ccbl1 |  |  | 15939 | 0.001 | 0.0443 | No |
| 1094 | 797439 | st6gal2b |  |  | 15960 | -0.000 | 0.0436 | No |
| 1095 | 402942 | pigf |  |  | 15979 | -0.001 | 0.0430 | No |
| 1096 | 563201 | pi4kb |  |  | 15995 | -0.001 | 0.0425 | No |
| 1097 | 562854 | lap3 |  |  | 16014 | -0.002 | 0.0419 | No |
| 1098 | 140512 | CYTB |  |  | 16039 | -0.003 | 0.0411 | No |
| 1099 | 393143 | umps |  |  | 16045 | -0.003 | 0.0409 | No |
| 1100 | 373867 | plcg1 |  |  | 16057 | -0.004 | 0.0405 | No |
| 1101 | 100003996\_1 | si:ch73-195i19.3 |  |  | 16063 | -0.004 | 0.0404 | No |
| 1102 | 560915 | rfk |  |  | 16071 | -0.004 | 0.0401 | No |
| 1103 | 563543 | inpp5b |  |  | 16108 | -0.005 | 0.0389 | No |
| 1104 | 563446 | xylt2 |  |  | 16113 | -0.006 | 0.0388 | No |
| 1105 | 791141 | zgc:158659 |  |  | 16140 | -0.006 | 0.0379 | No |
| 1106 | 64608 | alas1 |  |  | 16142 | -0.006 | 0.0379 | No |
| 1107 | 571426 | fkrp |  |  | 16155 | -0.007 | 0.0375 | No |
| 1108 | 100034522 | mthfd1l |  |  | 16165 | -0.007 | 0.0372 | No |
| 1109 | 541402 | asrgl1 |  |  | 16180 | -0.008 | 0.0367 | No |
| 1110 | 555470 | gphna |  |  | 16217 | -0.008 | 0.0355 | No |
| 1111 | 565386 | pla2g4f.2 |  |  | 16232 | -0.009 | 0.0351 | No |
| 1112 | 556193 | sgms2 |  |  | 16241 | -0.009 | 0.0348 | No |
| 1113 | 559560 | hykk.2 |  |  | 16258 | -0.009 | 0.0343 | No |
| 1114 | 569494\_1 | alg12 |  |  | 16265 | -0.010 | 0.0341 | No |
| 1115 | 100006345\_1 | fktn |  |  | 16294 | -0.011 | 0.0331 | No |
| 1116 | 548607 | b3gat1a |  |  | 16321 | -0.012 | 0.0323 | No |
| 1117 | 553326 | hacd1 |  |  | 16399 | -0.014 | 0.0297 | No |
| 1118 | 100331649 | dgki |  |  | 16424 | -0.015 | 0.0289 | No |
| 1119 | 556797 | pign |  |  | 16425 | -0.015 | 0.0289 | No |
| 1120 | 100331902 | mocos |  |  | 16432 | -0.016 | 0.0287 | No |
| 1121 | 407712\_1 | tph2 |  |  | 16433 | -0.016 | 0.0287 | No |
| 1122 | 555920 | pip4k2ab |  |  | 16439 | -0.016 | 0.0286 | No |
| 1123 | 100004977 | mthfd2 |  |  | 16483 | -0.017 | 0.0271 | No |
| 1124 | 450068 | asah1a |  |  | 16546 | -0.019 | 0.0250 | No |
| 1125 | 334116\_1 | eno1a |  |  | 16552 | -0.019 | 0.0249 | No |
| 1126 | 100332691 | si:ch73-173p19.2 |  |  | 16577 | -0.019 | 0.0241 | No |
| 1127 | 324188 | cyp26b1 |  |  | 16580 | -0.019 | 0.0240 | No |
| 1128 | 559240 | gba2 |  |  | 16594 | -0.020 | 0.0236 | No |
| 1129 | 436664 | zgc:92040 |  |  | 16627 | -0.021 | 0.0226 | No |
| 1130 | 100036767 | plod2 |  |  | 16643 | -0.021 | 0.0221 | No |
| 1131 | 334561 | acsl4b |  |  | 16648 | -0.022 | 0.0219 | No |
| 1132 | 554103 | cox7a2a |  |  | 16682 | -0.022 | 0.0209 | No |
| 1133 | 100003546 | colgalt2 |  |  | 16683 | -0.022 | 0.0209 | No |
| 1134 | 562774\_1 | p4ha1a |  |  | 16689 | -0.023 | 0.0207 | No |
| 1135 | 445175 | acsl1b |  |  | 16699 | -0.023 | 0.0204 | No |
| 1136 | 563972 | gstt1a |  |  | 16711 | -0.023 | 0.0201 | No |
| 1137 | 563180 | g6pca.2 |  |  | 16729 | -0.023 | 0.0195 | No |
| 1138 | 334116 | eno1a |  |  | 16760 | -0.025 | 0.0185 | No |
| 1139 | 798918 | ahcyl1 |  |  | 16766 | -0.025 | 0.0184 | No |
| 1140 | 100006320 | ugt2a1 |  |  | 16772 | -0.025 | 0.0182 | No |
| 1141 | 563369 | cyp2r1 |  |  | 16792 | -0.026 | 0.0176 | No |
| 1142 | 402841 | adh8b |  |  | 16797 | -0.026 | 0.0175 | No |
| 1143 | 550327 | pcyt1aa |  |  | 16808 | -0.027 | 0.0172 | No |
| 1144 | 327075 | degs1 |  |  | 16838 | -0.028 | 0.0162 | No |
| 1145 | 568143\_1 | car15 |  |  | 16908 | -0.031 | 0.0139 | No |
| 1146 | 393335\_2 | hsd17b3 |  |  | 16922 | -0.032 | 0.0135 | No |
| 1147 | 393970 | acy1 |  |  | 16923 | -0.032 | 0.0135 | No |
| 1148 | 797296 | prdm2b |  |  | 16933 | -0.032 | 0.0132 | No |
| 1149 | 140622 | atic |  |  | 16975 | -0.033 | 0.0119 | No |
| 1150 | 559305 | b4galnt4b |  |  | 17006 | -0.034 | 0.0109 | No |
| 1151 | 393123 | sepsecs |  |  | 17049 | -0.034 | 0.0095 | No |
| 1152 | 30505\_1 | dbh |  |  | 17052 | -0.035 | 0.0095 | No |
| 1153 | 101886626 | acyp2 |  |  | 17055 | -0.035 | 0.0094 | No |
| 1154 | 560219\_2 | man2a1 |  |  | 17066 | -0.035 | 0.0091 | No |
| 1155 | 101882295 | ggt7 |  |  | 17094 | -0.036 | 0.0082 | No |
| 1156 | 767653 | elovl8a |  |  | 17124 | -0.037 | 0.0073 | No |
| 1157 | 563799 | ash1l |  |  | 17129 | -0.037 | 0.0072 | No |
| 1158 | 394246 | pafah1b1a |  |  | 17138 | -0.038 | 0.0069 | No |
| 1159 | 569348\_1 | h6pd |  |  | 17148 | -0.038 | 0.0066 | No |
| 1160 | 556544 | si:dkey-199f5.8 |  |  | 17157 | -0.039 | 0.0064 | No |
| 1161 | 393556\_1 | gstt1b |  |  | 17163 | -0.039 | 0.0063 | No |
| 1162 | 562399 | enpp1 |  |  | 17165 | -0.039 | 0.0062 | No |
| 1163 | 558508 | ugt1b1 |  |  | 17170 | -0.040 | 0.0061 | No |
| 1164 | 327346 | mtmr6 |  |  | 17173 | -0.040 | 0.0061 | No |
| 1165 | 573021 | ndufb9 |  |  | 17203 | -0.041 | 0.0052 | No |
| 1166 | 415243 | ndufa1 |  |  | 17214 | -0.041 | 0.0048 | No |
| 1167 | 100537318 | ndufv3 |  |  | 17248 | -0.043 | 0.0038 | No |
| 1168 | 569027 | smyd1b |  |  | 17254 | -0.043 | 0.0036 | No |
| 1169 | 568256\_1 | asmt2 |  |  | 17277 | -0.044 | 0.0029 | No |
| 1170 | 798226 | ndst2b |  |  | 17286 | -0.044 | 0.0027 | No |
| 1171 | 100007286 | si:dkey-175m17.6 |  |  | 17289 | -0.045 | 0.0026 | No |
| 1172 | 564649 | osxm |  |  | 17307 | -0.045 | 0.0021 | No |
| 1173 | 405861 | pccb |  |  | 17313 | -0.046 | 0.0020 | No |
| 1174 | 393526 | mtap |  |  | 17330 | -0.047 | 0.0015 | No |
| 1175 | novel.329 | - |  |  | 17345 | -0.047 | 0.0010 | No |
| 1176 | 570368\_1 | arg1 |  |  | 17347 | -0.048 | 0.0010 | No |
| 1177 | novel.839 | - |  |  | 17355 | -0.048 | 0.0008 | No |
| 1178 | 569499 | adcy1b |  |  | 17365 | -0.048 | 0.0005 | No |
| 1179 | 100538291 | si:ch73-49o8.1 |  |  | 17366 | -0.048 | 0.0006 | No |
| 1180 | 333974 | gclm |  |  | 17390 | -0.049 | -0.0002 | No |
| 1181 | 368410 | pde6a |  |  | 17400 | -0.050 | -0.0004 | No |
| 1182 | 751742 | uraha |  |  | 17428 | -0.051 | -0.0013 | No |
| 1183 | 550370 | nudt12 |  |  | 17434 | -0.051 | -0.0014 | No |
| 1184 | 140519 | ATP6 |  |  | 17469 | -0.052 | -0.0025 | No |
| 1185 | 192334 | b3gat3 |  |  | 17485 | -0.053 | -0.0030 | No |
| 1186 | 558496 | cerk |  |  | 17489 | -0.053 | -0.0031 | No |
| 1187 | 445022 | b4galt7 |  |  | 17532 | -0.055 | -0.0045 | No |
| 1188 | 555748 | ugp2a |  |  | 17542 | -0.055 | -0.0047 | No |
| 1189 | 796025 | zgc:162544 |  |  | 17543 | -0.055 | -0.0047 | No |
| 1190 | 572849\_1 | suv420h1 |  |  | 17564 | -0.057 | -0.0053 | No |
| 1191 | 100000275 | LOC100000275 |  |  | 17574 | -0.057 | -0.0056 | No |
| 1192 | 555496 | gpaa1 |  |  | 17595 | -0.058 | -0.0062 | No |
| 1193 | 553952 | ugt2a2 |  |  | 17597 | -0.058 | -0.0062 | No |
| 1194 | 797715 | ogdhb |  |  | 17610 | -0.059 | -0.0066 | No |
| 1195 | 321245 | smyd1a |  |  | 17615 | -0.059 | -0.0067 | No |
| 1196 | 559736 | galnt12 |  |  | 17618 | -0.059 | -0.0067 | No |
| 1197 | 768131 | setdb1b |  |  | 17628 | -0.059 | -0.0069 | No |
| 1198 | 553629 | pip5k1aa |  |  | 17647 | -0.060 | -0.0075 | No |
| 1199 | 101886411 | ovgp1 |  |  | 17656 | -0.061 | -0.0077 | No |
| 1200 | 402985 | grhprb |  |  | 17657 | -0.061 | -0.0077 | No |
| 1201 | 431724\_2 | impdh1a |  |  | 17661 | -0.061 | -0.0078 | No |
| 1202 | 569507 | smyd3 |  |  | 17678 | -0.061 | -0.0083 | No |
| 1203 | 557048 | kmt2a |  |  | 17682 | -0.062 | -0.0083 | No |
| 1204 | 570678 | glyctk |  |  | 17707 | -0.063 | -0.0091 | No |
| 1205 | 560717 | srd5a3 |  |  | 17721 | -0.064 | -0.0095 | No |
| 1206 | 559228 | hyal2b |  |  | 17740 | -0.064 | -0.0100 | No |
| 1207 | 387290 | smox |  |  | 17750 | -0.065 | -0.0103 | No |
| 1208 | 336492 | ptgdsb.1 |  |  | 17752 | -0.065 | -0.0103 | No |
| 1209 | 100037331 | coq2 |  |  | 17757 | -0.065 | -0.0104 | No |
| 1210 | 569041\_1 | suv420h2 |  |  | 17759 | -0.065 | -0.0104 | No |
| 1211 | 335191 | atp5o |  |  | 17761 | -0.066 | -0.0103 | No |
| 1212 | 564619 | gstr |  |  | 17772 | -0.066 | -0.0106 | No |
| 1213 | 405776 | glcea |  |  | 17790 | -0.067 | -0.0112 | No |
| 1214 | 324211 | namptb |  |  | 17801 | -0.068 | -0.0114 | No |
| 1215 | 100003900 | chia.5 |  |  | 17813 | -0.068 | -0.0118 | No |
| 1216 | 567548 | smpd2a |  |  | 17857 | -0.070 | -0.0132 | No |
| 1217 | 553292 | setdb1a |  |  | 17866 | -0.070 | -0.0134 | No |
| 1218 | 100000559 | vkorc1 |  |  | 17868 | -0.070 | -0.0134 | No |
| 1219 | 678522 | gpat4 |  |  | 17897 | -0.071 | -0.0143 | No |
| 1220 | 100144628 | shmt2 |  |  | 17906 | -0.072 | -0.0145 | No |
| 1221 | 562357 | si:dkey-219c10.4 |  |  | 17913 | -0.072 | -0.0146 | No |
| 1222 | 554124\_1 | bckdha |  |  | 17916 | -0.072 | -0.0147 | No |
| 1223 | 553210 | st8sia1 |  |  | 17924 | -0.072 | -0.0148 | No |
| 1224 | 550338 | alad |  |  | 17925 | -0.072 | -0.0148 | No |
| 1225 | 403068 | alg2 |  |  | 17946 | -0.073 | -0.0154 | No |
| 1226 | 394174 | pik3cd |  |  | 17974 | -0.074 | -0.0163 | No |
| 1227 | 447864 | ass1 |  |  | 17996 | -0.075 | -0.0169 | No |
| 1228 | 555308 | acsl1a |  |  | 18027 | -0.077 | -0.0179 | No |
| 1229 | 556236 | acacb |  |  | 18028 | -0.077 | -0.0178 | No |
| 1230 | 449835 | dut |  |  | 18046 | -0.078 | -0.0183 | No |
| 1231 | 100006350 | whsc1l1 |  |  | 18064 | -0.079 | -0.0189 | No |
| 1232 | 100148732 | ggt1l2.1 |  |  | 18065 | -0.079 | -0.0188 | No |
| 1233 | 563589 | chkb |  |  | 18080 | -0.080 | -0.0192 | No |
| 1234 | 445121 | hadh |  |  | 18086 | -0.080 | -0.0193 | No |
| 1235 | 494099 | ephx2 |  |  | 18092 | -0.081 | -0.0195 | No |
| 1236 | 100005565 | si:ch211-287c22.1 |  |  | 18106 | -0.081 | -0.0198 | No |
| 1237 | 373132 | p4ha2 |  |  | 18121 | -0.082 | -0.0202 | No |
| 1238 | 557817\_1 | enpp7.2 |  |  | 18126 | -0.082 | -0.0203 | No |
| 1239 | 557979 | aadat |  |  | 18128 | -0.082 | -0.0203 | No |
| 1240 | 100317089 | inpp5jb |  |  | 18154 | -0.083 | -0.0211 | No |
| 1241 | 559504 | coq7 |  |  | 18187 | -0.085 | -0.0221 | No |
| 1242 | 100538007 | arsb |  |  | 18209 | -0.085 | -0.0228 | No |
| 1243 | 100537787 | ca12 |  |  | 18219 | -0.086 | -0.0230 | No |
| 1244 | 567414\_1 | agpat9l |  |  | 18220 | -0.086 | -0.0229 | No |
| 1245 | 560881 | mtm1 |  |  | 18231 | -0.087 | -0.0232 | No |
| 1246 | 101885014 | LOC101885014 |  |  | 18243 | -0.087 | -0.0235 | No |
| 1247 | 406298 | ndufa4 |  |  | 18253 | -0.088 | -0.0238 | No |
| 1248 | 571003 | b3galt1b |  |  | 18267 | -0.088 | -0.0241 | No |
| 1249 | 100535432 | ltc4s |  |  | 18269 | -0.088 | -0.0241 | No |
| 1250 | 562219 | ndufb11 |  |  | 18273 | -0.088 | -0.0242 | No |
| 1251 | 557628 | si:dkey-239i20.4 |  |  | 18276 | -0.088 | -0.0242 | No |
| 1252 | 449784 | mgst1.1 |  |  | 18288 | -0.089 | -0.0245 | No |
| 1253 | 541479 | hmgcrb |  |  | 18296 | -0.089 | -0.0246 | No |
| 1254 | 100003745 | sgpp1 |  |  | 18308 | -0.089 | -0.0250 | No |
| 1255 | 368912 | echs1 |  |  | 18310 | -0.089 | -0.0249 | No |
| 1256 | 335974 | got2b |  |  | 18318 | -0.090 | -0.0251 | No |
| 1257 | 563597 | si:ch211-243j20.2 |  |  | 18339 | -0.091 | -0.0257 | No |
| 1258 | 492796 | zgc:101663 |  |  | 18372 | -0.092 | -0.0267 | No |
| 1259 | 794199 | setd2 |  |  | 18374 | -0.092 | -0.0267 | No |
| 1260 | 560627 | LOC560627 |  |  | 18375 | -0.093 | -0.0266 | No |
| 1261 | 324307 | atp6v0a1a |  |  | 18391 | -0.093 | -0.0271 | No |
| 1262 | 415208 | zgc:86811 |  |  | 18397 | -0.093 | -0.0272 | No |
| 1263 | 567275 | mgst3b |  |  | 18399 | -0.094 | -0.0271 | No |
| 1264 | 100005761 | ehmt1a |  |  | 18425 | -0.095 | -0.0279 | No |
| 1265 | 322605 | chpt1 |  |  | 18433 | -0.095 | -0.0281 | No |
| 1266 | 334897\_1 | rpe |  |  | 18449 | -0.096 | -0.0285 | No |
| 1267 | 553283 | pcxa |  |  | 18484 | -0.098 | -0.0296 | No |
| 1268 | 406383 | cmpk |  |  | 18494 | -0.099 | -0.0298 | No |
| 1269 | 724006 | mgat5 |  |  | 18499 | -0.099 | -0.0299 | No |
| 1270 | 100333702 | adcy9 |  |  | 18501 | -0.099 | -0.0299 | No |
| 1271 | 541347 | mat2b |  |  | 18514 | -0.099 | -0.0302 | No |
| 1272 | 556700 | smpd1 |  |  | 18519 | -0.100 | -0.0303 | No |
| 1273 | 393753 | ppcdc |  |  | 18583 | -0.102 | -0.0323 | No |
| 1274 | 101884228 | LOC101884228 |  |  | 18589 | -0.103 | -0.0324 | No |
| 1275 | 100141344 | asmt |  |  | 18595 | -0.103 | -0.0325 | No |
| 1276 | 100331984 | galnt3 |  |  | 18619 | -0.104 | -0.0332 | No |
| 1277 | 324340 | cyp3c1 |  |  | 18620 | -0.104 | -0.0331 | No |
| 1278 | 550282 | dcxr |  |  | 18627 | -0.105 | -0.0333 | No |
| 1279 | 325395 | kmt2e |  |  | 18629 | -0.105 | -0.0332 | No |
| 1280 | 562619 | adcy5 |  |  | 18631 | -0.105 | -0.0332 | No |
| 1281 | 571344 | crtc1b |  |  | 18652 | -0.106 | -0.0338 | No |
| 1282 | 368220 | adka |  |  | 18659 | -0.106 | -0.0339 | No |
| 1283 | 334161 | alg1 |  |  | 18686 | -0.108 | -0.0347 | No |
| 1284 | 567938 | pik3c2b |  |  | 18707 | -0.109 | -0.0353 | No |
| 1285 | 406528 | tdh |  |  | 18717 | -0.109 | -0.0355 | No |
| 1286 | 560273 | oxct1b |  |  | 18736 | -0.110 | -0.0361 | No |
| 1287 | 58016 | hccsb |  |  | 18743 | -0.111 | -0.0362 | No |
| 1288 | 553671\_2 | ak8 |  |  | 18745 | -0.111 | -0.0362 | No |
| 1289 | 569040 | plcd3a |  |  | 18746 | -0.111 | -0.0361 | No |
| 1290 | 553750 | mtmr1a |  |  | 18773 | -0.112 | -0.0369 | No |
| 1291 | 751653 | st6galnac4 |  |  | 18793 | -0.113 | -0.0374 | No |
| 1292 | 100005067\_2 | dguok |  |  | 18799 | -0.113 | -0.0375 | No |
| 1293 | 569930 | zgc:153896 |  |  | 18818 | -0.114 | -0.0381 | No |
| 1294 | 393829 | ndufb8 |  |  | 18820 | -0.114 | -0.0380 | No |
| 1295 | 317738 | elovl6 |  |  | 18840 | -0.116 | -0.0386 | No |
| 1296 | 100333269 | aldh1l2 |  |  | 18841 | -0.116 | -0.0385 | No |
| 1297 | 791145 | pde8a |  |  | 18860 | -0.116 | -0.0390 | No |
| 1298 | 641570 | impad1 |  |  | 18887 | -0.117 | -0.0398 | No |
| 1299 | 81541 | atp5g3b |  |  | 18895 | -0.118 | -0.0400 | No |
| 1300 | 325875 | dgat1a |  |  | 18907 | -0.118 | -0.0402 | No |
| 1301 | 407989 | adssl1 |  |  | 18908 | -0.118 | -0.0402 | No |
| 1302 | 564111 | mcat |  |  | 18915 | -0.118 | -0.0403 | No |
| 1303 | 561030 | pdxp |  |  | 18923 | -0.119 | -0.0404 | No |
| 1304 | 678545 | gmpr2 |  |  | 18925 | -0.119 | -0.0404 | No |
| 1305 | 436894 | gsto1 |  |  | 18959 | -0.120 | -0.0414 | No |
| 1306 | 404620 | cdipt |  |  | 18977 | -0.120 | -0.0419 | No |
| 1307 | 192336 | atp6v0ca |  |  | 18982 | -0.121 | -0.0420 | No |
| 1308 | 394000 | pgm1 |  |  | 18989 | -0.121 | -0.0421 | No |
| 1309 | 406342 | tcirg1b |  |  | 18994 | -0.121 | -0.0421 | No |
| 1310 | 567587\_1 | mthfd1a |  |  | 19035 | -0.123 | -0.0434 | No |
| 1311 | 100333333\_1 | mgat4c |  |  | 19057 | -0.124 | -0.0440 | No |
| 1312 | 100004566 | atp6ap1a |  |  | 19068 | -0.125 | -0.0443 | No |
| 1313 | 378727 | pck1 |  |  | 19075 | -0.125 | -0.0444 | No |
| 1314 | 567592\_1 | smpd5 |  |  | 19076 | -0.125 | -0.0443 | No |
| 1315 | 751691 | coq6 |  |  | 19078 | -0.125 | -0.0442 | No |
| 1316 | 337690 | pikfyve |  |  | 19096 | -0.126 | -0.0447 | No |
| 1317 | 569770 | cox8b |  |  | 19098 | -0.126 | -0.0447 | No |
| 1318 | 327462\_1 | hsd3b7 |  |  | 19139 | -0.128 | -0.0459 | No |
| 1319 | 100124609 | zgc:165582 |  |  | 19158 | -0.129 | -0.0464 | No |
| 1320 | 553508 | ugt8 |  |  | 19174 | -0.130 | -0.0469 | No |
| 1321 | 402979 | cda |  |  | 19179 | -0.130 | -0.0469 | No |
| 1322 | 561683 | nt5c1bb |  |  | 19180 | -0.130 | -0.0468 | No |
| 1323 | 100136866 | zgc:172295 |  |  | 19211 | -0.131 | -0.0477 | No |
| 1324 | 548607\_1 | b3gat1a |  |  | 19218 | -0.132 | -0.0478 | No |
| 1325 | 563993 | pigo |  |  | 19220 | -0.132 | -0.0478 | No |
| 1326 | 561272 | ppcs |  |  | 19223 | -0.132 | -0.0478 | No |
| 1327 | 436771 | hccsa.1 |  |  | 19227 | -0.132 | -0.0478 | No |
| 1328 | 553660 | ndufs5 |  |  | 19236 | -0.132 | -0.0479 | No |
| 1329 | 100000775 | glula |  |  | 19256 | -0.133 | -0.0485 | No |
| 1330 | 768133 | ezh2 |  |  | 19275 | -0.134 | -0.0490 | No |
| 1331 | 504040 | ak7b |  |  | 19290 | -0.135 | -0.0494 | No |
| 1332 | 378441 | gad1b |  |  | 19299 | -0.135 | -0.0496 | No |
| 1333 | 399662 | mdh1aa |  |  | 19301 | -0.135 | -0.0495 | No |
| 1334 | 557181 | alg13 |  |  | 19311 | -0.136 | -0.0497 | No |
| 1335 | 564613 | abhd16a |  |  | 19349 | -0.137 | -0.0509 | No |
| 1336 | 100006589\_2 | idh1 |  |  | 19353 | -0.138 | -0.0509 | No |
| 1337 | 114426 | odc1 |  |  | 19382 | -0.139 | -0.0517 | No |
| 1338 | 570695 | mboat2a |  |  | 19401 | -0.139 | -0.0522 | No |
| 1339 | 393867 | ampd1 |  |  | 19427 | -0.140 | -0.0530 | No |
| 1340 | 450050 | dhrs3b |  |  | 19435 | -0.141 | -0.0531 | No |
| 1341 | 567157 | acsl3a |  |  | 19451 | -0.142 | -0.0535 | No |
| 1342 | 553524 | b4galnt3b |  |  | 19452 | -0.142 | -0.0534 | No |
| 1343 | 550259 | pfklb |  |  | 19460 | -0.142 | -0.0535 | No |
| 1344 | 541390 | ndufc2 |  |  | 19471 | -0.143 | -0.0538 | No |
| 1345 | 492781 | mvda |  |  | 19472 | -0.143 | -0.0537 | No |
| 1346 | 541328 | ndufa5 |  |  | 19478 | -0.143 | -0.0538 | No |
| 1347 | 406385 | galcb |  |  | 19487 | -0.143 | -0.0539 | No |
| 1348 | 571737 | LOC571737 |  |  | 19490 | -0.143 | -0.0539 | No |
| 1349 | 791844 | ca15b |  |  | 19536 | -0.145 | -0.0553 | No |
| 1350 | 335159 | acsbg2 |  |  | 19541 | -0.145 | -0.0553 | No |
| 1351 | 368477 | chsy3 |  |  | 19552 | -0.146 | -0.0556 | No |
| 1352 | 564697 | pdxkb |  |  | 19596 | -0.148 | -0.0569 | No |
| 1353 | 100384901 | ugt1a4 |  |  | 19602 | -0.148 | -0.0570 | No |
| 1354 | 406726 | ndufa4l |  |  | 19614 | -0.149 | -0.0573 | No |
| 1355 | 406496 | aldoab |  |  | 19616 | -0.149 | -0.0572 | No |
| 1356 | 192315 | uxs1 |  |  | 19639 | -0.150 | -0.0578 | No |
| 1357 | 405867 | crls1 |  |  | 19647 | -0.150 | -0.0580 | No |
| 1358 | 324201 | dlat |  |  | 19665 | -0.151 | -0.0584 | No |
| 1359 | 100334568 | pde5aa |  |  | 19678 | -0.152 | -0.0587 | No |
| 1360 | 336257 | nmnat2 |  |  | 19719 | -0.154 | -0.0600 | No |
| 1361 | 407683 | ca4c |  |  | 19736 | -0.154 | -0.0604 | No |
| 1362 | 100142647 | mogat3a |  |  | 19741 | -0.155 | -0.0604 | No |
| 1363 | 619269 | agxt2 |  |  | 19742 | -0.155 | -0.0603 | No |
| 1364 | 100150304 | gucy1b3 |  |  | 19772 | -0.156 | -0.0612 | No |
| 1365 | 321928 | phgdh |  |  | 19781 | -0.156 | -0.0613 | No |
| 1366 | 100535634 | pip4k2cb |  |  | 19806 | -0.158 | -0.0620 | No |
| 1367 | 541416 | arsa |  |  | 19811 | -0.158 | -0.0621 | No |
| 1368 | 678543 | zgc:136858 |  |  | 19814 | -0.158 | -0.0620 | No |
| 1369 | 795959 | si:dkey-222h21.12 |  |  | 19859 | -0.160 | -0.0634 | No |
| 1370 | 792692 | aldoca |  |  | 19868 | -0.160 | -0.0635 | No |
| 1371 | 321294 | cpox |  |  | 19879 | -0.161 | -0.0638 | No |
| 1372 | 64263 | gch2 |  |  | 19886 | -0.161 | -0.0639 | No |
| 1373 | 796410 | fut9a |  |  | 19909 | -0.162 | -0.0645 | No |
| 1374 | 378968 | abat |  |  | 19919 | -0.162 | -0.0647 | No |
| 1375 | 393361 | pigm |  |  | 19935 | -0.163 | -0.0651 | No |
| 1376 | 799492 | ckmt2b |  |  | 19936 | -0.163 | -0.0650 | No |
| 1377 | 556528 | rdh5 |  |  | 19969 | -0.165 | -0.0659 | No |
| 1378 | 449801 | zgc:103601 |  |  | 20012 | -0.167 | -0.0672 | No |
| 1379 | 436622 | zgc:92162 |  |  | 20033 | -0.168 | -0.0678 | No |
| 1380 | 552981 | gpx7 |  |  | 20037 | -0.168 | -0.0678 | No |
| 1381 | 100006448\_1 | ca6 |  |  | 20066 | -0.169 | -0.0686 | No |
| 1382 | 504044 | st3gal4 |  |  | 20068 | -0.169 | -0.0685 | No |
| 1383 | 79381 | gstp1 |  |  | 20086 | -0.170 | -0.0690 | No |
| 1384 | 393556 | gstt1b |  |  | 20106 | -0.171 | -0.0695 | No |
| 1385 | 325561 | rpn1 |  |  | 20109 | -0.171 | -0.0694 | No |
| 1386 | 436597 | rdh12 |  |  | 20148 | -0.173 | -0.0706 | No |
| 1387 | 100536469 | LOC100536469 |  |  | 20155 | -0.173 | -0.0707 | No |
| 1388 | 436852 | gcshb |  |  | 20182 | -0.174 | -0.0714 | No |
| 1389 | 494076\_1 | dhtkd1 |  |  | 20185 | -0.174 | -0.0714 | No |
| 1390 | 30381 | cyp26a1 |  |  | 20201 | -0.175 | -0.0718 | No |
| 1391 | 327288 | cndp2 |  |  | 20209 | -0.175 | -0.0719 | No |
| 1392 | 140534 | ND4 |  |  | 20213 | -0.175 | -0.0719 | No |
| 1393 | 100536119 | LOC100536119 |  |  | 20233 | -0.176 | -0.0724 | No |
| 1394 | 321621 | gldc |  |  | 20262 | -0.177 | -0.0732 | No |
| 1395 | 563594 | st6galnac5a |  |  | 20321 | -0.180 | -0.0750 | No |
| 1396 | 415243\_1 | ndufa1 |  |  | 20329 | -0.180 | -0.0751 | No |
| 1397 | 394052 | ftcd |  |  | 20350 | -0.181 | -0.0757 | No |
| 1398 | 445097 | gmppb |  |  | 20390 | -0.182 | -0.0769 | No |
| 1399 | 394129 | hmbsa |  |  | 20401 | -0.183 | -0.0771 | No |
| 1400 | 557756\_1 | enpp7.1 |  |  | 20424 | -0.184 | -0.0777 | No |
| 1401 | 606663 | psph |  |  | 20450 | -0.185 | -0.0784 | No |
| 1402 | 406405 | mdh2 |  |  | 20457 | -0.185 | -0.0785 | No |
| 1403 | 337784 | hlcs |  |  | 20473 | -0.186 | -0.0788 | No |
| 1404 | 569376 | plcb2 |  |  | 20503 | -0.188 | -0.0797 | No |
| 1405 | 561410 | tat |  |  | 20583 | -0.193 | -0.0822 | No |
| 1406 | 569184\_1 | si:dkey-19b23.15 |  |  | 20590 | -0.193 | -0.0823 | No |
| 1407 | 321793 | ak2 |  |  | 20598 | -0.193 | -0.0824 | No |
| 1408 | 555196 | ca4a |  |  | 20599 | -0.193 | -0.0823 | No |
| 1409 | 100141367 | cers3b |  |  | 20602 | -0.194 | -0.0822 | No |
| 1410 | 100332034 | ndufb3 |  |  | 20605 | -0.194 | -0.0821 | No |
| 1411 | 415192 | pnp4a |  |  | 20628 | -0.195 | -0.0827 | No |
| 1412 | 751635 | alg5 |  |  | 20636 | -0.195 | -0.0828 | No |
| 1413 | 768185 | hsd17b7 |  |  | 20645 | -0.196 | -0.0830 | No |
| 1414 | 402795 | sts |  |  | 20651 | -0.196 | -0.0830 | No |
| 1415 | 406428 | pdhb |  |  | 20690 | -0.198 | -0.0841 | No |
| 1416 | 554101 | cox5aa |  |  | 20691 | -0.198 | -0.0840 | No |
| 1417 | 445486 | ak1 |  |  | 20697 | -0.198 | -0.0840 | No |
| 1418 | 30084 | nme2b.2 |  |  | 20707 | -0.199 | -0.0842 | No |
| 1419 | 100004744 | LOC100004744 |  |  | 20726 | -0.200 | -0.0847 | No |
| 1420 | 557708 | si:ch211-199m3.4 |  |  | 20731 | -0.200 | -0.0847 | No |
| 1421 | 266987 | cbsb |  |  | 20738 | -0.200 | -0.0847 | No |
| 1422 | 563201\_1 | pi4kb |  |  | 20741 | -0.200 | -0.0846 | No |
| 1423 | 282559 | aldh3b1 |  |  | 20742 | -0.200 | -0.0845 | No |
| 1424 | 406651 | ddc |  |  | 20753 | -0.201 | -0.0847 | No |
| 1425 | 406359 | uqcrfs1 |  |  | 20779 | -0.203 | -0.0854 | No |
| 1426 | 335709 | atp5d |  |  | 20785 | -0.203 | -0.0854 | No |
| 1427 | 415227 | ptges3b |  |  | 20804 | -0.204 | -0.0859 | No |
| 1428 | 402883 | atp5j2 |  |  | 20808 | -0.204 | -0.0859 | No |
| 1429 | 566872 | l2hgdh |  |  | 20836 | -0.206 | -0.0866 | No |
| 1430 | 334494 | lipt1 |  |  | 20859 | -0.207 | -0.0872 | No |
| 1431 | 373092 | glud1b |  |  | 20882 | -0.209 | -0.0878 | No |
| 1432 | 571274\_1 | setd1bb |  |  | 20890 | -0.209 | -0.0879 | No |
| 1433 | 402822 | gcat |  |  | 20893 | -0.209 | -0.0878 | No |
| 1434 | 557614 | chac2 |  |  | 20898 | -0.209 | -0.0878 | No |
| 1435 | 447802 | coq5 |  |  | 20922 | -0.211 | -0.0884 | No |
| 1436 | 793471 | mocs1 |  |  | 20932 | -0.212 | -0.0886 | No |
| 1437 | 494065 | dhodh |  |  | 20934 | -0.212 | -0.0885 | No |
| 1438 | 492789 | zgc:101699 |  |  | 20960 | -0.213 | -0.0892 | No |
| 1439 | 494091 | adoa |  |  | 20975 | -0.214 | -0.0895 | No |
| 1440 | 553564\_1 | zgc:109982 |  |  | 20976 | -0.214 | -0.0894 | No |
| 1441 | 556077 | plod3 |  |  | 20985 | -0.215 | -0.0895 | No |
| 1442 | 369193 | aldocb |  |  | 21049 | -0.218 | -0.0914 | No |
| 1443 | 368242 | kmo |  |  | 21067 | -0.219 | -0.0919 | No |
| 1444 | 678540 | gale |  |  | 21068 | -0.219 | -0.0917 | No |
| 1445 | 406619 | nnt |  |  | 21069 | -0.219 | -0.0916 | No |
| 1446 | 335774 | cox6a1 |  |  | 21080 | -0.220 | -0.0917 | No |
| 1447 | 573013 | alox5b.1 |  |  | 21083 | -0.220 | -0.0917 | No |
| 1448 | 334197 | aldh7a1 |  |  | 21094 | -0.221 | -0.0918 | No |
| 1449 | 450016 | pcyt2 |  |  | 21122 | -0.222 | -0.0926 | No |
| 1450 | 566539 | dpagt1 |  |  | 21125 | -0.222 | -0.0925 | No |
| 1451 | 550403 | gad2 |  |  | 21132 | -0.223 | -0.0926 | No |
| 1452 | 447840 | pip5k1bb |  |  | 21133 | -0.223 | -0.0924 | No |
| 1453 | 791203 | spam1 |  |  | 21138 | -0.223 | -0.0924 | No |
| 1454 | 564379 | cyc1 |  |  | 21143 | -0.223 | -0.0924 | No |
| 1455 | 447913 | ndufs6 |  |  | 21171 | -0.225 | -0.0931 | No |
| 1456 | 555599 | pde4bb |  |  | 21179 | -0.225 | -0.0932 | No |
| 1457 | 567859 | si:ch211-114l13.7 |  |  | 21180 | -0.225 | -0.0930 | No |
| 1458 | 556445 | gls2b |  |  | 21211 | -0.226 | -0.0939 | No |
| 1459 | 569053 | plpp1a |  |  | 21219 | -0.226 | -0.0940 | No |
| 1460 | 406482 | gdpd3a |  |  | 21222 | -0.226 | -0.0939 | No |
| 1461 | 569250 | ehmt2 |  |  | 21254 | -0.228 | -0.0948 | No |
| 1462 | 393641 | aprt |  |  | 21266 | -0.228 | -0.0950 | No |
| 1463 | 394240 | ahcyl2 |  |  | 21271 | -0.229 | -0.0950 | No |
| 1464 | 566723 | uqcrh |  |  | 21287 | -0.230 | -0.0953 | No |
| 1465 | 393478 | cox6b2 |  |  | 21307 | -0.231 | -0.0958 | No |
| 1466 | 553655 | pygma |  |  | 21309 | -0.231 | -0.0957 | No |
| 1467 | 394021 | shmt1 |  |  | 21319 | -0.232 | -0.0958 | No |
| 1468 | 100004274 | cyp8b2 |  |  | 21320 | -0.232 | -0.0956 | No |
| 1469 | 338290 | hmbsb |  |  | 21341 | -0.233 | -0.0961 | No |
| 1470 | 553639 | gmpr |  |  | 21373 | -0.235 | -0.0970 | No |
| 1471 | 101886595 | b3galt4 |  |  | 21399 | -0.236 | -0.0977 | No |
| 1472 | 100332644 | b3gat1b |  |  | 21401 | -0.236 | -0.0976 | No |
| 1473 | 797211 | ugt2a6 |  |  | 21441 | -0.238 | -0.0987 | No |
| 1474 | 555899 | si:ch211-160o17.2 |  |  | 21462 | -0.239 | -0.0992 | No |
| 1475 | 406599 | atp5j |  |  | 21464 | -0.239 | -0.0991 | No |
| 1476 | 445290 | acat1 |  |  | 21467 | -0.239 | -0.0990 | No |
| 1477 | 393700\_1 | ndufa8 |  |  | 21478 | -0.240 | -0.0992 | No |
| 1478 | 100333727 | zmp:0000001132 |  |  | 21483 | -0.240 | -0.0991 | No |
| 1479 | 567051 | pde3a |  |  | 21489 | -0.240 | -0.0991 | No |
| 1480 | 678541 | gstk4 |  |  | 21503 | -0.241 | -0.0994 | No |
| 1481 | 751732 | zgc:153247 |  |  | 21541 | -0.243 | -0.1005 | No |
| 1482 | 100535363 | cox7a2b |  |  | 21548 | -0.243 | -0.1005 | No |
| 1483 | 80872 | sms |  |  | 21550 | -0.244 | -0.1004 | No |
| 1484 | 559945 | dot1l |  |  | 21571 | -0.244 | -0.1009 | No |
| 1485 | 266799 | gatm |  |  | 21574 | -0.245 | -0.1008 | No |
| 1486 | 101886904 | csgalnact1b |  |  | 21581 | -0.245 | -0.1008 | No |
| 1487 | 794259 | aclyb |  |  | 21582 | -0.245 | -0.1007 | No |
| 1488 | 373124 | pip4k2ca |  |  | 21609 | -0.246 | -0.1014 | No |
| 1489 | 393781 | ndufb6 |  |  | 21625 | -0.247 | -0.1017 | No |
| 1490 | 100537860 | LOC100537860 |  |  | 21649 | -0.248 | -0.1023 | No |
| 1491 | 559332 | pde6b |  |  | 21663 | -0.249 | -0.1026 | No |
| 1492 | 393335\_1 | hsd17b3 |  |  | 21700 | -0.251 | -0.1036 | No |
| 1493 | 116713 | aldh1a2 |  |  | 21717 | -0.252 | -0.1040 | No |
| 1494 | 797549 | galnt9 |  |  | 21726 | -0.253 | -0.1041 | No |
| 1495 | 559298 | entpd2a.2 |  |  | 21728 | -0.253 | -0.1039 | No |
| 1496 | 450000\_1 | amt |  |  | 21766 | -0.255 | -0.1050 | No |
| 1497 | 553384\_1 | cox10 |  |  | 21784 | -0.256 | -0.1054 | No |
| 1498 | 325712 | ndufab1b |  |  | 21801 | -0.257 | -0.1057 | No |
| 1499 | 100001552 | pcyt1ba |  |  | 21804 | -0.257 | -0.1056 | No |
| 1500 | 567798 | agla |  |  | 21813 | -0.257 | -0.1057 | No |
| 1501 | 566165 | glulc |  |  | 21829 | -0.258 | -0.1060 | No |
| 1502 | 101883995 | pde1ca |  |  | 21831 | -0.258 | -0.1059 | No |
| 1503 | 393703 | ndufb10 |  |  | 21856 | -0.259 | -0.1065 | No |
| 1504 | 664754 | ezh1 |  |  | 21872 | -0.260 | -0.1068 | No |
| 1505 | 100332846 | acsl2 |  |  | 21878 | -0.261 | -0.1068 | No |
| 1506 | 553795 | nanp |  |  | 21880 | -0.261 | -0.1067 | No |
| 1507 | 798716 | ispd |  |  | 21918 | -0.263 | -0.1077 | No |
| 1508 | 100536156 | LOC100536156 |  |  | 21946 | -0.264 | -0.1085 | No |
| 1509 | 564575 | setmar |  |  | 21969 | -0.266 | -0.1090 | No |
| 1510 | 100536819\_1 | LOC100536819 |  |  | 21974 | -0.266 | -0.1090 | No |
| 1511 | 558541 | cyp3c4 |  |  | 21982 | -0.266 | -0.1090 | No |
| 1512 | 798606 | tecrl2a |  |  | 22001 | -0.267 | -0.1095 | No |
| 1513 | 564336 | st6galnac1.1 |  |  | 22007 | -0.268 | -0.1094 | No |
| 1514 | 393984 | aacs |  |  | 22011 | -0.268 | -0.1094 | No |
| 1515 | 100333801 | LOC100333801 |  |  | 22027 | -0.269 | -0.1097 | No |
| 1516 | 796865 | gamt |  |  | 22028 | -0.269 | -0.1095 | No |
| 1517 | 436733 | zgc:92907 |  |  | 22074 | -0.271 | -0.1108 | No |
| 1518 | 553754 | st8sia5 |  |  | 22083 | -0.272 | -0.1109 | No |
| 1519 | 393675 | atp5h |  |  | 22091 | -0.272 | -0.1109 | No |
| 1520 | 563138\_1 | si:ch211-219a15.4 |  |  | 22095 | -0.272 | -0.1109 | No |
| 1521 | 322549 | uqcrc2b |  |  | 22169 | -0.276 | -0.1131 | No |
| 1522 | 445291 | ndufv1 |  |  | 22170 | -0.277 | -0.1129 | No |
| 1523 | 393876 | prdm9 |  |  | 22175 | -0.277 | -0.1129 | No |
| 1524 | 436850 | suclg1 |  |  | 22189 | -0.278 | -0.1131 | No |
| 1525 | 140538 | ND4L |  |  | 22200 | -0.278 | -0.1133 | No |
| 1526 | 450000 | amt |  |  | 22218 | -0.279 | -0.1136 | No |
| 1527 | 100141496 | dmgdh |  |  | 22235 | -0.280 | -0.1140 | No |
| 1528 | 100003066 | ndufa4l2a |  |  | 22239 | -0.281 | -0.1139 | No |
| 1529 | 393687 | ndufa13 |  |  | 22241 | -0.281 | -0.1137 | No |
| 1530 | 573723 | acadvl |  |  | 22257 | -0.282 | -0.1140 | No |
| 1531 | 140520 | ATP8 |  |  | 22265 | -0.282 | -0.1141 | No |
| 1532 | 724056 | alox5b.2 |  |  | 22270 | -0.282 | -0.1140 | No |
| 1533 | 100001676 | shpk |  |  | 22281 | -0.283 | -0.1142 | No |
| 1534 | 378722 | rdh10b |  |  | 22283 | -0.283 | -0.1140 | No |
| 1535 | 317745 | impdh2 |  |  | 22289 | -0.283 | -0.1140 | No |
| 1536 | 436930 | uqcrc2a |  |  | 22306 | -0.284 | -0.1143 | No |
| 1537 | 100150090 | sphk2 |  |  | 22316 | -0.285 | -0.1144 | No |
| 1538 | 569581 | mut |  |  | 22328 | -0.286 | -0.1146 | No |
| 1539 | 445198 | suv39h1a |  |  | 22342 | -0.286 | -0.1148 | No |
| 1540 | 450052 | acot8 |  |  | 22347 | -0.287 | -0.1148 | No |
| 1541 | 445202\_1 | dpm1 |  |  | 22373 | -0.288 | -0.1154 | No |
| 1542 | 101884438 | LOC101884438 |  |  | 22382 | -0.289 | -0.1155 | No |
| 1543 | 553952\_1 | ugt2a2 |  |  | 22395 | -0.289 | -0.1157 | No |
| 1544 | 404633\_1 | b3galt2 |  |  | 22405 | -0.290 | -0.1158 | No |
| 1545 | 100004136 | ptdss2 |  |  | 22418 | -0.291 | -0.1160 | No |
| 1546 | 436968 | cox6b1 |  |  | 22427 | -0.291 | -0.1160 | No |
| 1547 | 30095\_1 | ckma |  |  | 22428 | -0.291 | -0.1158 | No |
| 1548 | 327165 | pgam1b |  |  | 22471 | -0.294 | -0.1170 | No |
| 1549 | 100148976\_1 | adcy8 |  |  | 22476 | -0.295 | -0.1170 | No |
| 1550 | 100334707 | LOC100334707 |  |  | 22479 | -0.295 | -0.1168 | No |
| 1551 | 437022 | tyrp1b |  |  | 22480 | -0.295 | -0.1166 | No |
| 1552 | 30496 | ldha |  |  | 22490 | -0.295 | -0.1167 | No |
| 1553 | 562627 | nt5c1aa |  |  | 22497 | -0.296 | -0.1167 | No |
| 1554 | 541435 | acss1 |  |  | 22524 | -0.297 | -0.1174 | No |
| 1555 | 393455\_1 | hao2 |  |  | 22551 | -0.298 | -0.1181 | No |
| 1556 | 492359 | zgc:91976 |  |  | 22557 | -0.299 | -0.1180 | No |
| 1557 | 259189 | hgd |  |  | 22562 | -0.299 | -0.1180 | No |
| 1558 | 554135 | atp5b |  |  | 22579 | -0.300 | -0.1183 | No |
| 1559 | 564552 | ogdha |  |  | 22588 | -0.301 | -0.1183 | No |
| 1560 | 449987 | zgc:103759 |  |  | 22590 | -0.301 | -0.1182 | No |
| 1561 | 568219\_1 | si:ch73-139e5.4 |  |  | 22604 | -0.302 | -0.1184 | No |
| 1562 | 449537 | hsd17b2 |  |  | 22608 | -0.302 | -0.1183 | No |
| 1563 | 796865\_1 | gamt |  |  | 22611 | -0.302 | -0.1182 | No |
| 1564 | 100005614 | si:ch211-287c22.2p |  |  | 22632 | -0.304 | -0.1186 | No |
| 1565 | 406554 | lclat1 |  |  | 22664 | -0.305 | -0.1194 | No |
| 1566 | 449945 | pgm2l1 |  |  | 22697 | -0.307 | -0.1203 | No |
| 1567 | 571911 | fdft1 |  |  | 22728 | -0.309 | -0.1211 | No |
| 1568 | 561303 | si:ch73-41h24.1 |  |  | 22757 | -0.311 | -0.1218 | No |
| 1569 | 567970\_1 | setd1ba |  |  | 22766 | -0.311 | -0.1219 | No |
| 1570 | 378446 | dhcr7 |  |  | 22772 | -0.312 | -0.1218 | No |
| 1571 | 393884 | sdha |  |  | 22785 | -0.312 | -0.1220 | No |
| 1572 | 793671 | csgalnact1a |  |  | 22806 | -0.314 | -0.1225 | No |
| 1573 | 336118 | cox7c |  |  | 22818 | -0.314 | -0.1226 | No |
| 1574 | 101884755 | LOC101884755 |  |  | 22823 | -0.315 | -0.1225 | No |
| 1575 | 449787 | atp5f1 |  |  | 22827 | -0.315 | -0.1224 | No |
| 1576 | 100000709\_2 | whsc1 |  |  | 22849 | -0.316 | -0.1229 | No |
| 1577 | 553755 | atp5a1 |  |  | 22852 | -0.316 | -0.1228 | No |
| 1578 | 556680 | mtfmt |  |  | 22853 | -0.316 | -0.1225 | No |
| 1579 | 406762 | pgd |  |  | 22903 | -0.320 | -0.1240 | No |
| 1580 | 393127 | pemt |  |  | 22908 | -0.320 | -0.1239 | No |
| 1581 | 368213 | glo1 |  |  | 22941 | -0.323 | -0.1247 | No |
| 1582 | 558524 | mgat1b |  |  | 22948 | -0.323 | -0.1247 | No |
| 1583 | 566565\_2 | mars2 |  |  | 22951 | -0.324 | -0.1246 | No |
| 1584 | 447810 | nt5c2l1 |  |  | 22960 | -0.324 | -0.1246 | No |
| 1585 | 338183 | mars |  |  | 22995 | -0.327 | -0.1255 | No |
| 1586 | 394077 | pde10a |  |  | 23006 | -0.328 | -0.1256 | No |
| 1587 | 394135 | hibadhb |  |  | 23012 | -0.328 | -0.1256 | No |
| 1588 | 445060 | b3gat2 |  |  | 23015 | -0.328 | -0.1254 | No |
| 1589 | 445494 | pgls |  |  | 23031 | -0.330 | -0.1257 | No |
| 1590 | 570007 | inpp5f |  |  | 23049 | -0.331 | -0.1260 | No |
| 1591 | 322089 | ctps1a |  |  | 23055 | -0.331 | -0.1260 | No |
| 1592 | 492719 | me3 |  |  | 23069 | -0.332 | -0.1262 | No |
| 1593 | 321720 | nt5c3a |  |  | 23073 | -0.332 | -0.1260 | No |
| 1594 | 30083 | nme2b.1 |  |  | 23078 | -0.333 | -0.1260 | No |
| 1595 | 751701 | ptgdsb.2 |  |  | 23079 | -0.333 | -0.1257 | No |
| 1596 | 564746 | glsb |  |  | 23111 | -0.335 | -0.1265 | No |
| 1597 | 393782 | dpm3 |  |  | 23112 | -0.335 | -0.1263 | No |
| 1598 | 373131 | hsd3b2 |  |  | 23113 | -0.335 | -0.1261 | No |
| 1599 | 556280 | cyp2u1 |  |  | 23137 | -0.336 | -0.1266 | No |
| 1600 | 567897 | ca14 |  |  | 23156 | -0.338 | -0.1270 | No |
| 1601 | 378963 | eno3 |  |  | 23157 | -0.338 | -0.1267 | No |
| 1602 | 100006238 | aldh9a1a.2 |  |  | 23161 | -0.338 | -0.1266 | No |
| 1603 | 415176 | nudt5 |  |  | 23183 | -0.340 | -0.1271 | No |
| 1604 | 556667 | pigt |  |  | 23192 | -0.341 | -0.1271 | No |
| 1605 | 100334231 | plch2a |  |  | 23206 | -0.341 | -0.1273 | No |
| 1606 | 108179059 | LOC108179059 |  |  | 23224 | -0.343 | -0.1277 | No |
| 1607 | 436958 | gpat3 |  |  | 23225 | -0.343 | -0.1274 | No |
| 1608 | 100334384 | gpx2 |  |  | 23238 | -0.343 | -0.1276 | No |
| 1609 | 572061 | pde4a |  |  | 23250 | -0.344 | -0.1277 | No |
| 1610 | 569734 | ggt5a |  |  | 23261 | -0.345 | -0.1278 | No |
| 1611 | 406283 | acadm |  |  | 23289 | -0.347 | -0.1285 | No |
| 1612 | 767664 | cox7a1 |  |  | 23291 | -0.347 | -0.1283 | No |
| 1613 | 555330\_1 | etnk2 |  |  | 23319 | -0.349 | -0.1289 | No |
| 1614 | 450083\_1 | gla |  |  | 23329 | -0.350 | -0.1290 | No |
| 1615 | 393497 | ndufa11 |  |  | 23330 | -0.350 | -0.1288 | No |
| 1616 | 79378 | agxtb |  |  | 23331 | -0.350 | -0.1285 | No |
| 1617 | 100415792 | ugt5b3 |  |  | 23332 | -0.350 | -0.1283 | No |
| 1618 | 436903 | bpgm |  |  | 23363 | -0.352 | -0.1290 | No |
| 1619 | 30384\_1 | th |  |  | 23375 | -0.353 | -0.1292 | No |
| 1620 | 402874 | eno2 |  |  | 23379 | -0.353 | -0.1290 | No |
| 1621 | 558775 | sirt3 |  |  | 23401 | -0.354 | -0.1295 | No |
| 1622 | 798974 | nadsyn1 |  |  | 23419 | -0.356 | -0.1298 | No |
| 1623 | 445500 | sdhdb |  |  | 23455 | -0.358 | -0.1307 | No |
| 1624 | 100333333 | mgat4c |  |  | 23463 | -0.359 | -0.1307 | No |
| 1625 | 100006320\_1 | ugt2a1 |  |  | 23473 | -0.359 | -0.1308 | No |
| 1626 | 567794 | galnt17 |  |  | 23476 | -0.359 | -0.1306 | No |
| 1627 | 335025 | atp6v1g1 |  |  | 23477 | -0.359 | -0.1303 | No |
| 1628 | 791759 | piga |  |  | 23501 | -0.361 | -0.1309 | No |
| 1629 | 100002263 | pik3cb |  |  | 23508 | -0.361 | -0.1308 | No |
| 1630 | 326975 | cox4i1 |  |  | 23518 | -0.362 | -0.1309 | No |
| 1631 | 322229 | adkb |  |  | 23529 | -0.363 | -0.1310 | No |
| 1632 | 494055\_1 | sat2a |  |  | 23545 | -0.364 | -0.1312 | No |
| 1633 | 550502 | adss |  |  | 23578 | -0.365 | -0.1320 | No |
| 1634 | 555206\_1 | neu3.3 |  |  | 23654 | -0.371 | -0.1343 | No |
| 1635 | 100415794 | ugt5d1 |  |  | 23662 | -0.371 | -0.1343 | No |
| 1636 | 100333145 | tyrp1a |  |  | 23687 | -0.372 | -0.1348 | No |
| 1637 | 550565\_1 | gnpda1 |  |  | 23723 | -0.375 | -0.1358 | No |
| 1638 | 436827 | sat2b |  |  | 23725 | -0.375 | -0.1355 | No |
| 1639 | 559850 | neu1 |  |  | 23773 | -0.378 | -0.1368 | No |
| 1640 | 393365 | mtmr8 |  |  | 23794 | -0.380 | -0.1373 | No |
| 1641 | 326962 | cox5ab |  |  | 23797 | -0.380 | -0.1371 | No |
| 1642 | 556956 | pi4kaa |  |  | 23804 | -0.381 | -0.1370 | No |
| 1643 | 402880 | cox4i1l |  |  | 23812 | -0.381 | -0.1370 | No |
| 1644 | 664750 | ppox |  |  | 23822 | -0.381 | -0.1370 | No |
| 1645 | 606579 | inpp5e |  |  | 23836 | -0.382 | -0.1372 | No |
| 1646 | 557077 | mmab |  |  | 23838 | -0.382 | -0.1369 | No |
| 1647 | 386951 | idh2 |  |  | 23851 | -0.383 | -0.1371 | No |
| 1648 | 664772 | hyal1 |  |  | 23871 | -0.384 | -0.1375 | No |
| 1649 | 100037338 | inpp5ka |  |  | 23878 | -0.385 | -0.1374 | No |
| 1650 | 550494 | esd |  |  | 23886 | -0.385 | -0.1374 | No |
| 1651 | 550451 | ndufs3 |  |  | 23915 | -0.387 | -0.1380 | No |
| 1652 | 100332076 | gcnt3 |  |  | 23921 | -0.388 | -0.1379 | No |
| 1653 | 566188\_1 | inpp5kb |  |  | 23930 | -0.388 | -0.1379 | No |
| 1654 | 402953 | pnp6 |  |  | 23940 | -0.388 | -0.1380 | No |
| 1655 | 553532 | hsd3b1 |  |  | 23947 | -0.389 | -0.1379 | No |
| 1656 | 550586 | enpp4 |  |  | 23988 | -0.391 | -0.1390 | No |
| 1657 | 326954 | tecrb |  |  | 23996 | -0.391 | -0.1389 | No |
| 1658 | 322055 | cth |  |  | 23997 | -0.391 | -0.1387 | No |
| 1659 | 368775 | ivd |  |  | 23998 | -0.391 | -0.1384 | No |
| 1660 | 402826 | si:ch73-71d17.1 |  |  | 24027 | -0.394 | -0.1391 | No |
| 1661 | 557008 | aldh3b2 |  |  | 24033 | -0.394 | -0.1390 | No |
| 1662 | 570547\_1 | uckl1a |  |  | 24045 | -0.396 | -0.1391 | No |
| 1663 | 553582 | tusc3 |  |  | 24050 | -0.396 | -0.1389 | No |
| 1664 | 442923 | oxct1a |  |  | 24062 | -0.397 | -0.1390 | No |
| 1665 | 563432 | si:ch211-79e4.4 |  |  | 24070 | -0.397 | -0.1390 | No |
| 1666 | 565706 | pde4ba |  |  | 24095 | -0.399 | -0.1395 | No |
| 1667 | 567286 | gdpd3b |  |  | 24136 | -0.402 | -0.1406 | No |
| 1668 | 548346 | pik3c3 |  |  | 24150 | -0.403 | -0.1407 | No |
| 1669 | 445492 | flad1 |  |  | 24167 | -0.404 | -0.1410 | No |
| 1670 | 560778 | naga |  |  | 24171 | -0.404 | -0.1408 | No |
| 1671 | 322780 | nansa |  |  | 24184 | -0.405 | -0.1409 | No |
| 1672 | 450002 | tecrl2b |  |  | 24206 | -0.407 | -0.1414 | No |
| 1673 | 393935 | atp6v1d |  |  | 24235 | -0.409 | -0.1420 | No |
| 1674 | 565295 | cers2b |  |  | 24254 | -0.411 | -0.1423 | No |
| 1675 | 336425 | aldoaa |  |  | 24256 | -0.411 | -0.1421 | No |
| 1676 | 394110\_1 | atp6v1aa |  |  | 24258 | -0.411 | -0.1418 | No |
| 1677 | 30644 | mtmr2 |  |  | 24276 | -0.412 | -0.1421 | No |
| 1678 | 767778 | agpat5 |  |  | 24278 | -0.412 | -0.1419 | No |
| 1679 | 554125 | ndufa2 |  |  | 24293 | -0.414 | -0.1421 | No |
| 1680 | 406661 | nudt9 |  |  | 24294 | -0.414 | -0.1418 | No |
| 1681 | 322811\_1 | atp6v0d1 |  |  | 24364 | -0.415 | -0.1438 | No |
| 1682 | 558473 | cyp3c2 |  |  | 24384 | -0.416 | -0.1442 | No |
| 1683 | 555308\_1 | acsl1a |  |  | 24418 | -0.416 | -0.1450 | No |
| 1684 | 100002384 | cox5b1 |  |  | 24457 | -0.417 | -0.1460 | No |
| 1685 | 337583\_1 | atp6v1ab |  |  | 24458 | -0.417 | -0.1457 | No |
| 1686 | 445129\_2 | sdhc |  |  | 24579 | -0.420 | -0.1494 | No |
| 1687 | 399692 | cyp17a1 |  |  | 24603 | -0.422 | -0.1499 | No |
| 1688 | 492778 | acot19 |  |  | 24610 | -0.422 | -0.1498 | No |
| 1689 | 394075 | minpp1b |  |  | 24618 | -0.423 | -0.1498 | No |
| 1690 | 100149204 | ggcta |  |  | 24620 | -0.423 | -0.1495 | No |
| 1691 | 100333321 | prodha |  |  | 24638 | -0.424 | -0.1498 | No |
| 1692 | 446213 | large |  |  | 24659 | -0.426 | -0.1502 | No |
| 1693 | 30085 | nme3 |  |  | 24681 | -0.428 | -0.1506 | No |
| 1694 | 556744 | uroc1 |  |  | 24709 | -0.431 | -0.1512 | No |
| 1695 | 406259\_1 | hprt1 |  |  | 24744 | -0.434 | -0.1520 | No |
| 1696 | 553672 | ndufs2 |  |  | 24748 | -0.434 | -0.1518 | No |
| 1697 | 84039 | bco1 |  |  | 24749 | -0.434 | -0.1515 | No |
| 1698 | 386801 | agps |  |  | 24764 | -0.436 | -0.1517 | No |
| 1699 | 100331460 | LOC100331460 |  |  | 24852 | -0.440 | -0.1543 | No |
| 1700 | 100331020 | chdh |  |  | 24917 | -0.442 | -0.1562 | No |
| 1701 | 556086 | nsd1a |  |  | 24921 | -0.442 | -0.1559 | No |
| 1702 | 415253 | zgc:86599 |  |  | 24928 | -0.443 | -0.1558 | No |
| 1703 | 402868 | pnpo |  |  | 24967 | -0.444 | -0.1568 | No |
| 1704 | 100005704 | si:dkey-110k5.10 |  |  | 24994 | -0.445 | -0.1574 | No |
| 1705 | 317737 | glud1a |  |  | 25006 | -0.446 | -0.1574 | No |
| 1706 | 100333777 | hsd11b1lb |  |  | 25013 | -0.447 | -0.1573 | No |
| 1707 | 399479 | dldh |  |  | 25027 | -0.448 | -0.1575 | No |
| 1708 | 58074 | dct |  |  | 25044 | -0.450 | -0.1577 | No |
| 1709 | 541388 | dbt |  |  | 25050 | -0.451 | -0.1575 | No |
| 1710 | 406799 | sdr16c5b |  |  | 25052 | -0.451 | -0.1573 | No |
| 1711 | 393423 | asl |  |  | 25054 | -0.451 | -0.1570 | No |
| 1712 | 544666 | ptges |  |  | 25057 | -0.451 | -0.1567 | No |
| 1713 | 100538220 | atp6v1c2 |  |  | 25059 | -0.451 | -0.1565 | No |
| 1714 | 565329 | sqleb |  |  | 25077 | -0.452 | -0.1567 | No |
| 1715 | 563343\_1 | prune |  |  | 25082 | -0.453 | -0.1565 | No |
| 1716 | 246095 | gpib |  |  | 25103 | -0.454 | -0.1569 | No |
| 1717 | 334431\_2 | adsl |  |  | 25123 | -0.455 | -0.1572 | No |
| 1718 | 797132 | sirt1 |  |  | 25130 | -0.456 | -0.1571 | No |
| 1719 | 336957 | atp5c1 |  |  | 25134 | -0.456 | -0.1569 | No |
| 1720 | 324366 | gstm.1 |  |  | 25161 | -0.457 | -0.1575 | No |
| 1721 | 572070 | si:ch73-170l17.1 |  |  | 25194 | -0.459 | -0.1582 | No |
| 1722 | 415158 | cycsb |  |  | 25213 | -0.461 | -0.1585 | No |
| 1723 | 564867 | cers6 |  |  | 25216 | -0.461 | -0.1582 | No |
| 1724 | 101884360 | LOC101884360 |  |  | 25255 | -0.464 | -0.1592 | No |
| 1725 | 445562 | st3gal2 |  |  | 25275 | -0.465 | -0.1595 | No |
| 1726 | 565184\_1 | b4galnt4a |  |  | 25285 | -0.465 | -0.1595 | No |
| 1727 | 100151036 | ipmka |  |  | 25302 | -0.467 | -0.1597 | No |
| 1728 | 100151256 | cmbl |  |  | 25329 | -0.469 | -0.1603 | No |
| 1729 | 449655 | atp6v1c1b |  |  | 25334 | -0.469 | -0.1601 | No |
| 1730 | 101885478 | LOC101885478 |  |  | 25342 | -0.470 | -0.1600 | No |
| 1731 | 393493\_1 | gstt2 |  |  | 25358 | -0.472 | -0.1602 | No |
| 1732 | 799449 | nt5c1ab |  |  | 25372 | -0.473 | -0.1603 | No |
| 1733 | 100330695 | galnt13 |  |  | 25374 | -0.473 | -0.1600 | No |
| 1734 | 492477 | mvk |  |  | 25399 | -0.475 | -0.1605 | No |
| 1735 | 445129 | sdhc |  |  | 25446 | -0.479 | -0.1617 | No |
| 1736 | 570652\_2 | adcy6a |  |  | 25465 | -0.481 | -0.1619 | No |
| 1737 | 791518 | hmox1a |  |  | 25490 | -0.483 | -0.1624 | No |
| 1738 | 559958 | cdaa |  |  | 25499 | -0.485 | -0.1624 | No |
| 1739 | 64815 | hsd17b8 |  |  | 25500 | -0.485 | -0.1620 | No |
| 1740 | 561172 | b3gnt3 |  |  | 25511 | -0.486 | -0.1620 | No |
| 1741 | 58141 | gart |  |  | 25513 | -0.486 | -0.1617 | No |
| 1742 | 557716 | nags |  |  | 25514 | -0.486 | -0.1614 | No |
| 1743 | 326977 | atp5l |  |  | 25518 | -0.486 | -0.1611 | No |
| 1744 | 553569 | neu4 |  |  | 25521 | -0.486 | -0.1609 | No |
| 1745 | 571561 | LOC571561 |  |  | 25547 | -0.489 | -0.1614 | No |
| 1746 | 564750 | pi4k2b |  |  | 25560 | -0.490 | -0.1614 | No |
| 1747 | 393926 | idh3a |  |  | 25570 | -0.491 | -0.1614 | No |
| 1748 | 30207 | tyr |  |  | 25585 | -0.492 | -0.1615 | No |
| 1749 | 795400 | lss |  |  | 25608 | -0.494 | -0.1619 | No |
| 1750 | 100151110 | oplah |  |  | 25631 | -0.496 | -0.1623 | No |
| 1751 | 406301 | atp5g1 |  |  | 25665 | -0.500 | -0.1631 | No |
| 1752 | 445245\_1 | ctps1b |  |  | 25666 | -0.500 | -0.1627 | No |
| 1753 | 550322 | nmnat1 |  |  | 25673 | -0.500 | -0.1626 | No |
| 1754 | 573689\_1 | ndufs4 |  |  | 25674 | -0.500 | -0.1622 | No |
| 1755 | 799805 | akr1a1b |  |  | 25680 | -0.501 | -0.1621 | No |
| 1756 | 564261\_1 | si:rp71-17i16.5 |  |  | 25681 | -0.501 | -0.1617 | No |
| 1757 | 559635 | hykk.1 |  |  | 25689 | -0.501 | -0.1616 | No |
| 1758 | 359839\_1 | atp6v1ba |  |  | 25690 | -0.502 | -0.1613 | No |
| 1759 | 562592 | man1b1a |  |  | 25693 | -0.502 | -0.1610 | No |
| 1760 | 561891 | minpp1a |  |  | 25702 | -0.503 | -0.1609 | No |
| 1761 | 445242 | hnmt |  |  | 25723 | -0.505 | -0.1612 | No |
| 1762 | 327453 | pfkfb1 |  |  | 25729 | -0.505 | -0.1610 | No |
| 1763 | 323918 | stt3b |  |  | 25760 | -0.508 | -0.1617 | No |
| 1764 | 335985\_1 | rpn2 |  |  | 25766 | -0.509 | -0.1615 | No |
| 1765 | 103911376 | si:ch211-226l4.6 |  |  | 25780 | -0.510 | -0.1616 | No |
| 1766 | 568547 | galnt8a.1 |  |  | 25796 | -0.510 | -0.1617 | No |
| 1767 | 393776 | cox4i2 |  |  | 25810 | -0.512 | -0.1618 | No |
| 1768 | 566323 | qdpra |  |  | 25822 | -0.512 | -0.1618 | No |
| 1769 | 100148976 | adcy8 |  |  | 25826 | -0.513 | -0.1616 | No |
| 1770 | 568713 | dse |  |  | 25841 | -0.514 | -0.1617 | No |
| 1771 | 559103\_2 | inpp5d |  |  | 25865 | -0.517 | -0.1621 | No |
| 1772 | 494577 | cox6c |  |  | 25868 | -0.517 | -0.1618 | No |
| 1773 | 571441 | gusb |  |  | 25884 | -0.518 | -0.1620 | No |
| 1774 | 100332468 | pde2a |  |  | 25894 | -0.520 | -0.1619 | No |
| 1775 | 436799 | atp6v1f |  |  | 25896 | -0.520 | -0.1616 | No |
| 1776 | 436768\_1 | uqcrq |  |  | 25910 | -0.521 | -0.1617 | No |
| 1777 | 447919 | aspdh |  |  | 25911 | -0.521 | -0.1613 | No |
| 1778 | 100000352 | si:dkey-152b24.7 |  |  | 25918 | -0.522 | -0.1611 | No |
| 1779 | 793294 | smpd4 |  |  | 25959 | -0.526 | -0.1621 | No |
| 1780 | 562250 | fut7 |  |  | 25960 | -0.526 | -0.1618 | No |
| 1781 | 402868\_1 | pnpo |  |  | 25967 | -0.526 | -0.1616 | No |
| 1782 | 567720\_1 | idua |  |  | 25969 | -0.527 | -0.1613 | No |
| 1783 | 553691 | atp6v0a1b |  |  | 25976 | -0.527 | -0.1611 | No |
| 1784 | 195824 | atp6ap1b |  |  | 25978 | -0.527 | -0.1608 | No |
| 1785 | 393864 | naprt |  |  | 25979 | -0.528 | -0.1604 | No |
| 1786 | 794752 | ckmb |  |  | 25980 | -0.528 | -0.1600 | No |
| 1787 | 445071 | nadk2 |  |  | 25984 | -0.528 | -0.1598 | No |
| 1788 | 493605 | ndufs1 |  |  | 25990 | -0.528 | -0.1596 | No |
| 1789 | 554136 | spra |  |  | 26002 | -0.529 | -0.1596 | No |
| 1790 | 560020 | acss2l |  |  | 26041 | -0.533 | -0.1605 | No |
| 1791 | 246226 | ptgs1 |  |  | 26048 | -0.533 | -0.1603 | No |
| 1792 | 60658 | nos1 |  |  | 26051 | -0.533 | -0.1600 | No |
| 1793 | 559537 | scly |  |  | 26112 | -0.538 | -0.1617 | No |
| 1794 | 100379286 | ugt1a2 |  |  | 26116 | -0.539 | -0.1614 | No |
| 1795 | 100150965\_1 | si:dkey-73n8.3 |  |  | 26117 | -0.539 | -0.1610 | No |
| 1796 | 393425 | elovl5 |  |  | 26119 | -0.539 | -0.1607 | No |
| 1797 | 558050\_1 | si:ch211-127i16.2 |  |  | 26135 | -0.540 | -0.1608 | No |
| 1798 | 447848 | cyp8b3 |  |  | 26136 | -0.541 | -0.1604 | No |
| 1799 | 557348 | cox8a |  |  | 26159 | -0.544 | -0.1608 | No |
| 1800 | 567881 | sat1b |  |  | 26164 | -0.544 | -0.1606 | No |
| 1801 | 192335 | atp6v1e1b |  |  | 26172 | -0.545 | -0.1604 | No |
| 1802 | 553944 | ugcg |  |  | 26190 | -0.546 | -0.1606 | No |
| 1803 | 570106 | pfkla |  |  | 26195 | -0.547 | -0.1604 | No |
| 1804 | 100331575 | alg10 |  |  | 26216 | -0.549 | -0.1607 | No |
| 1805 | 565184 | b4galnt4a |  |  | 26236 | -0.551 | -0.1609 | No |
| 1806 | 393839 | scp2a |  |  | 26251 | -0.552 | -0.1610 | No |
| 1807 | 541449 | zgc:113516 |  |  | 26276 | -0.554 | -0.1614 | No |
| 1808 | 337166 | si:dkey-40g16.6 |  |  | 26277 | -0.554 | -0.1610 | No |
| 1809 | 563363 | cyp11a2 |  |  | 26292 | -0.556 | -0.1611 | No |
| 1810 | 436729 | setd7 |  |  | 26299 | -0.557 | -0.1609 | No |
| 1811 | 322372 | fah |  |  | 26329 | -0.559 | -0.1615 | No |
| 1812 | 553716 | b3galnt2 |  |  | 26336 | -0.560 | -0.1613 | No |
| 1813 | 286779 | atp6v1h |  |  | 26354 | -0.562 | -0.1615 | No |
| 1814 | 393769 | elovl4a |  |  | 26365 | -0.563 | -0.1615 | No |
| 1815 | 436646 | galm |  |  | 26367 | -0.564 | -0.1611 | No |
| 1816 | 793046\_1 | neu3.4 |  |  | 26386 | -0.566 | -0.1613 | No |
| 1817 | 101886093\_1 | si:ch211-264e16.1 |  |  | 26388 | -0.566 | -0.1610 | No |
| 1818 | 406831 | gyg1a |  |  | 26398 | -0.567 | -0.1609 | No |
| 1819 | 557232 | aspa |  |  | 26403 | -0.567 | -0.1606 | No |
| 1820 | 557205 | phykpl |  |  | 26437 | -0.571 | -0.1613 | No |
| 1821 | 100331155 | chpfa |  |  | 26471 | -0.575 | -0.1620 | No |
| 1822 | 767705\_1 | ugt2a5 |  |  | 26500 | -0.577 | -0.1626 | No |
| 1823 | 431716 | sat1a.2 |  |  | 26516 | -0.580 | -0.1627 | No |
| 1824 | 641423 | zgc:123275 |  |  | 26519 | -0.580 | -0.1624 | No |
| 1825 | 393305 | hyi |  |  | 26530 | -0.582 | -0.1623 | No |
| 1826 | 568448 | aco1 |  |  | 26535 | -0.582 | -0.1620 | No |
| 1827 | 335716 | nme2a |  |  | 26542 | -0.583 | -0.1618 | No |
| 1828 | 566487 | gbgt1l2 |  |  | 26562 | -0.584 | -0.1621 | No |
| 1829 | 796749\_1 | si:ch73-54n14.2 |  |  | 26567 | -0.585 | -0.1618 | No |
| 1830 | 550396 | mppe1 |  |  | 26573 | -0.585 | -0.1615 | No |
| 1831 | 100415800 | ugt5f1 |  |  | 26575 | -0.585 | -0.1612 | No |
| 1832 | 335025\_1 | atp6v1g1 |  |  | 26589 | -0.587 | -0.1612 | No |
| 1833 | 386661 | scd |  |  | 26590 | -0.587 | -0.1608 | No |
| 1834 | 101882239 | LOC101882239 |  |  | 26601 | -0.588 | -0.1607 | No |
| 1835 | 368433 | inppl1b |  |  | 26634 | -0.591 | -0.1614 | No |
| 1836 | 553767 | atpv0e2 |  |  | 26644 | -0.592 | -0.1613 | No |
| 1837 | 338306 | impdh1b |  |  | 26651 | -0.593 | -0.1611 | No |
| 1838 | 393542 | selenbp1 |  |  | 26667 | -0.595 | -0.1612 | No |
| 1839 | 445376 | st6gal1 |  |  | 26671 | -0.595 | -0.1609 | No |
| 1840 | 327566 | lpcat4 |  |  | 26674 | -0.596 | -0.1605 | No |
| 1841 | 797063\_1 | ndufb5 |  |  | 26691 | -0.598 | -0.1606 | No |
| 1842 | 100384903 | ugt1b3 |  |  | 26756 | -0.605 | -0.1624 | No |
| 1843 | 798022 | nt5c1ba |  |  | 26775 | -0.606 | -0.1626 | No |
| 1844 | 563878 | pomt2 |  |  | 26786 | -0.607 | -0.1625 | No |
| 1845 | 393720 | ndufv2 |  |  | 26805 | -0.610 | -0.1627 | No |
| 1846 | 450054 | tpk1 |  |  | 26809 | -0.610 | -0.1623 | No |
| 1847 | 100002145\_1 | si:ch73-337l15.2 |  |  | 26810 | -0.610 | -0.1619 | No |
| 1848 | 405814 | sdr16c5a |  |  | 26816 | -0.610 | -0.1617 | No |
| 1849 | 791197 | zgc:158263 |  |  | 26866 | -0.615 | -0.1629 | No |
| 1850 | 557716\_1 | nags |  |  | 26881 | -0.617 | -0.1629 | No |
| 1851 | 352943 | tph1a |  |  | 26886 | -0.618 | -0.1626 | No |
| 1852 | 445118 | g6pca.1 |  |  | 26919 | -0.622 | -0.1633 | No |
| 1853 | 321951 | ndufa10 |  |  | 26930 | -0.623 | -0.1632 | No |
| 1854 | 436768 | uqcrq |  |  | 26944 | -0.624 | -0.1632 | No |
| 1855 | 541331 | ndufa9a |  |  | 26945 | -0.624 | -0.1627 | No |
| 1856 | 569184 | si:dkey-19b23.15 |  |  | 26982 | -0.628 | -0.1635 | No |
| 1857 | 567204\_1 | alox5a |  |  | 26999 | -0.628 | -0.1636 | No |
| 1858 | 393532 | pdhx |  |  | 27005 | -0.629 | -0.1634 | No |
| 1859 | 791130 | galt |  |  | 27007 | -0.629 | -0.1630 | No |
| 1860 | 393744 | dctpp1 |  |  | 27009 | -0.629 | -0.1626 | No |
| 1861 | 567170 | pafah1b2 |  |  | 27061 | -0.636 | -0.1638 | No |
| 1862 | 791448\_2 | qdprb1 |  |  | 27072 | -0.637 | -0.1637 | No |
| 1863 | 100415797 | ugt5a5 |  |  | 27081 | -0.638 | -0.1636 | No |
| 1864 | 100149843 | pip5kl1 |  |  | 27094 | -0.639 | -0.1635 | No |
| 1865 | 110439839 | LOC110439839 |  |  | 27117 | -0.641 | -0.1638 | No |
| 1866 | 100329944 | ndst1a |  |  | 27126 | -0.641 | -0.1636 | No |
| 1867 | 327057 | uck2b |  |  | 27131 | -0.641 | -0.1633 | No |
| 1868 | 558499 | chka |  |  | 27132 | -0.641 | -0.1629 | No |
| 1869 | 798364 | hibch |  |  | 27149 | -0.642 | -0.1630 | No |
| 1870 | 567869 | hyal2a |  |  | 27151 | -0.643 | -0.1626 | No |
| 1871 | 492735\_1 | si:ch211-212o1.2 |  |  | 27154 | -0.643 | -0.1622 | No |
| 1872 | 100331665 | LOC100331665 |  |  | 27187 | -0.644 | -0.1628 | No |
| 1873 | 64607 | alas2 |  |  | 27195 | -0.645 | -0.1626 | No |
| 1874 | 402904 | nit2 |  |  | 27232 | -0.646 | -0.1634 | No |
| 1875 | 445252 | hibadha |  |  | 27292 | -0.648 | -0.1649 | No |
| 1876 | 550533 | hddc3 |  |  | 27301 | -0.649 | -0.1647 | No |
| 1877 | 317746\_2 | suclg2 |  |  | 27320 | -0.651 | -0.1649 | No |
| 1878 | 792041\_1 | tbxas1 |  |  | 27376 | -0.657 | -0.1663 | No |
| 1879 | 100310831 | galnt18b |  |  | 27412 | -0.659 | -0.1670 | No |
| 1880 | 793524 | ndst2a |  |  | 27437 | -0.662 | -0.1673 | No |
| 1881 | 565372\_1 | hexdc |  |  | 27474 | -0.670 | -0.1681 | No |
| 1882 | 555271 | tmlhe |  |  | 27496 | -0.672 | -0.1683 | No |
| 1883 | 101886429 | LOC101886429 |  |  | 27507 | -0.674 | -0.1682 | No |
| 1884 | 431743 | gmppaa |  |  | 27518 | -0.675 | -0.1681 | No |
| 1885 | 554700 | pde6g |  |  | 27523 | -0.675 | -0.1677 | No |
| 1886 | 436833 | gstk1 |  |  | 27536 | -0.677 | -0.1677 | No |
| 1887 | 541317 | gatb |  |  | 27561 | -0.680 | -0.1680 | No |
| 1888 | 449558 | zgc:101723 |  |  | 27586 | -0.684 | -0.1683 | No |
| 1889 | 568915 | si:ch211-15e22.3 |  |  | 27595 | -0.685 | -0.1681 | No |
| 1890 | 100333064 | uqcr10 |  |  | 27599 | -0.685 | -0.1678 | No |
| 1891 | 415247 | idh3b |  |  | 27612 | -0.688 | -0.1677 | No |
| 1892 | 30659 | dnmt3bb.2 |  |  | 27619 | -0.689 | -0.1674 | No |
| 1893 | 352929 | gpx4b |  |  | 27634 | -0.691 | -0.1674 | No |
| 1894 | 445504 | gdpd1 |  |  | 27662 | -0.695 | -0.1678 | No |
| 1895 | 572733 | pgam2 |  |  | 27679 | -0.698 | -0.1679 | No |
| 1896 | 553762 | fahd1 |  |  | 27687 | -0.699 | -0.1676 | No |
| 1897 | 327329 | qrsl1 |  |  | 27700 | -0.701 | -0.1676 | No |
| 1898 | 767756 | ugt5a2 |  |  | 27723 | -0.704 | -0.1678 | No |
| 1899 | 554129 | hddc2 |  |  | 27728 | -0.706 | -0.1674 | No |
| 1900 | 780844 | neu3.1 |  |  | 27740 | -0.708 | -0.1673 | No |
| 1901 | 321664 | aldob |  |  | 27744 | -0.709 | -0.1669 | No |
| 1902 | 558186 | enosf1 |  |  | 27745 | -0.709 | -0.1664 | No |
| 1903 | 100148522 | si:ch211-217a12.1 |  |  | 27762 | -0.711 | -0.1665 | No |
| 1904 | 64265 | aox5 |  |  | 27764 | -0.711 | -0.1660 | No |
| 1905 | 553770 | pcyt1ab |  |  | 27776 | -0.712 | -0.1659 | No |
| 1906 | 572027 | ppat |  |  | 27788 | -0.714 | -0.1658 | No |
| 1907 | 794088\_2 | ptena |  |  | 27793 | -0.715 | -0.1654 | No |
| 1908 | 100006303\_1 | gls2a |  |  | 27798 | -0.715 | -0.1651 | No |
| 1909 | 678634 | mat2al |  |  | 27805 | -0.716 | -0.1648 | No |
| 1910 | 407712 | tph2 |  |  | 27844 | -0.721 | -0.1655 | No |
| 1911 | 562292 | acsf3 |  |  | 27857 | -0.723 | -0.1654 | No |
| 1912 | 792041 | tbxas1 |  |  | 27894 | -0.728 | -0.1662 | No |
| 1913 | 100334605 | fig4a |  |  | 27909 | -0.729 | -0.1661 | No |
| 1914 | 494176 | rdh12l |  |  | 27913 | -0.729 | -0.1657 | No |
| 1915 | 436731 | dgat1b |  |  | 27949 | -0.736 | -0.1664 | No |
| 1916 | 557187 | tmem5 |  |  | 27964 | -0.737 | -0.1663 | No |
| 1917 | 556538 | ndufa12 |  |  | 27983 | -0.740 | -0.1664 | No |
| 1918 | 664765 | lipt2 |  |  | 28001 | -0.742 | -0.1665 | No |
| 1919 | 406746\_1 | fpgs |  |  | 28002 | -0.742 | -0.1660 | No |
| 1920 | 393724 | rpe65a |  |  | 28009 | -0.743 | -0.1657 | No |
| 1921 | 323653 | aldh3a2a |  |  | 28024 | -0.745 | -0.1656 | No |
| 1922 | 447941 | apip |  |  | 28033 | -0.747 | -0.1654 | No |
| 1923 | 100149324 | proca1 |  |  | 28036 | -0.747 | -0.1649 | No |
| 1924 | 334270\_1 | agk |  |  | 28037 | -0.747 | -0.1644 | No |
| 1925 | 493916 | pygl |  |  | 28092 | -0.756 | -0.1657 | No |
| 1926 | 554154 | uqcrb |  |  | 28106 | -0.757 | -0.1656 | No |
| 1927 | 100037349 | mlycd |  |  | 28119 | -0.759 | -0.1655 | No |
| 1928 | 559488 | papss1 |  |  | 28123 | -0.759 | -0.1651 | No |
| 1929 | 559103\_1 | inpp5d |  |  | 28125 | -0.760 | -0.1646 | No |
| 1930 | 799099 | lpin1 |  |  | 28128 | -0.760 | -0.1641 | No |
| 1931 | 436896 | zgc:92249 |  |  | 28129 | -0.760 | -0.1636 | No |
| 1932 | 445505 | fbp2 |  |  | 28134 | -0.761 | -0.1632 | No |
| 1933 | 562480 | si:dkey-72l14.3 |  |  | 28142 | -0.763 | -0.1629 | No |
| 1934 | 791775 | pdxka |  |  | 28149 | -0.764 | -0.1626 | No |
| 1935 | 393264 | uap1l1 |  |  | 28151 | -0.764 | -0.1621 | No |
| 1936 | 777616 | zgc:154054 |  |  | 28153 | -0.764 | -0.1616 | No |
| 1937 | 404036 | nos2a |  |  | 28157 | -0.765 | -0.1611 | No |
| 1938 | 317744 | dnmt3bb.1 |  |  | 28159 | -0.765 | -0.1606 | No |
| 1939 | 790916 | ipmkb |  |  | 28181 | -0.769 | -0.1608 | No |
| 1940 | 555478 | si:ch211-106j24.1 |  |  | 28214 | -0.774 | -0.1614 | No |
| 1941 | 116713\_1 | aldh1a2 |  |  | 28217 | -0.775 | -0.1609 | No |
| 1942 | 116517\_1 | adh5 |  |  | 28219 | -0.775 | -0.1604 | No |
| 1943 | 557518 | tkta |  |  | 28233 | -0.776 | -0.1603 | No |
| 1944 | 566506 | gnsa |  |  | 28238 | -0.777 | -0.1599 | No |
| 1945 | 571876 | pomgnt1 |  |  | 28247 | -0.777 | -0.1596 | No |
| 1946 | 327462 | hsd3b7 |  |  | 28270 | -0.781 | -0.1598 | No |
| 1947 | 550560 | entpd6 |  |  | 28271 | -0.781 | -0.1593 | No |
| 1948 | 798206 | zgc:194551 |  |  | 28282 | -0.783 | -0.1591 | No |
| 1949 | 562739 | galnt14 |  |  | 28288 | -0.784 | -0.1587 | No |
| 1950 | 100330875 | gucy1a2 |  |  | 28291 | -0.784 | -0.1582 | No |
| 1951 | 555411 | acer3 |  |  | 28300 | -0.786 | -0.1579 | No |
| 1952 | 394155 | gys1 |  |  | 28333 | -0.792 | -0.1585 | No |
| 1953 | 791189\_1 | hyal6 |  |  | 28363 | -0.797 | -0.1589 | No |
| 1954 | 100150463\_2 | dgke |  |  | 28377 | -0.800 | -0.1588 | No |
| 1955 | 373884 | cecr1a |  |  | 28385 | -0.801 | -0.1584 | No |
| 1956 | 327512 | psat1 |  |  | 28400 | -0.803 | -0.1584 | No |
| 1957 | 561018 | cmasa |  |  | 28409 | -0.805 | -0.1581 | No |
| 1958 | 553595 | impa2 |  |  | 28425 | -0.807 | -0.1580 | No |
| 1959 | 406801\_1 | acp5a |  |  | 28427 | -0.808 | -0.1575 | No |
| 1960 | 794132 | si:dkey-17e16.9 |  |  | 28455 | -0.812 | -0.1578 | No |
| 1961 | 767750\_1 | atp5ib |  |  | 28490 | -0.818 | -0.1584 | No |
| 1962 | novel.687 | - |  |  | 28540 | -0.829 | -0.1595 | No |
| 1963 | 751745 | zgc:153441 |  |  | 28599 | -0.839 | -0.1609 | No |
| 1964 | 100537991 | prodhb |  |  | 28640 | -0.845 | -0.1616 | No |
| 1965 | 494076 | dhtkd1 |  |  | 28644 | -0.846 | -0.1611 | No |
| 1966 | 555478\_1 | si:ch211-106j24.1 |  |  | 28649 | -0.847 | -0.1607 | No |
| 1967 | 503765 | carnmt1 |  |  | 28660 | -0.848 | -0.1604 | No |
| 1968 | 550369 | nsdhl |  |  | 28663 | -0.849 | -0.1599 | No |
| 1969 | 321218 | pigq |  |  | 28666 | -0.849 | -0.1594 | No |
| 1970 | 436922 | aclya |  |  | 28694 | -0.857 | -0.1597 | No |
| 1971 | 415146 | hprt1l |  |  | 28695 | -0.857 | -0.1591 | No |
| 1972 | 541317\_1 | gatb |  |  | 28709 | -0.860 | -0.1589 | No |
| 1973 | 368907 | atp6v1c1a |  |  | 28719 | -0.863 | -0.1587 | No |
| 1974 | 557125\_1 | si:dkey-103i16.6 |  |  | 28735 | -0.865 | -0.1586 | No |
| 1975 | 791124 | cyp11c1 |  |  | 28741 | -0.866 | -0.1581 | No |
| 1976 | 436595 | nudt2 |  |  | 28755 | -0.870 | -0.1580 | No |
| 1977 | 403051 | pygb |  |  | 28772 | -0.872 | -0.1579 | No |
| 1978 | 558337\_1 | cercam |  |  | 28814 | -0.881 | -0.1587 | No |
| 1979 | 768165 | dpyda.1 |  |  | 28815 | -0.881 | -0.1581 | No |
| 1980 | 619247 | aasdhppt |  |  | 28823 | -0.882 | -0.1577 | No |
| 1981 | 791915 | enpp6 |  |  | 28839 | -0.884 | -0.1576 | No |
| 1982 | 317738\_1 | elovl6 |  |  | 28857 | -0.889 | -0.1575 | No |
| 1983 | 796436 | lct |  |  | 28863 | -0.891 | -0.1571 | No |
| 1984 | 100330772 | ampd2a |  |  | 28873 | -0.894 | -0.1568 | No |
| 1985 | 553708 | zgc:112320 |  |  | 28890 | -0.896 | -0.1567 | No |
| 1986 | 437018 | impa1 |  |  | 28902 | -0.900 | -0.1564 | No |
| 1987 | 447836 | pfkma |  |  | 28916 | -0.902 | -0.1562 | No |
| 1988 | 100536673 | LOC100536673 |  |  | 28921 | -0.903 | -0.1557 | No |
| 1989 | 550372 | afmid |  |  | 28942 | -0.907 | -0.1558 | No |
| 1990 | 100141353 | si:dkey-94e7.2 |  |  | 28985 | -0.916 | -0.1566 | No |
| 1991 | 555623 | cps1 |  |  | 28996 | -0.919 | -0.1563 | No |
| 1992 | 572841\_1 | mboat1 |  |  | 29023 | -0.926 | -0.1565 | No |
| 1993 | 100317129\_2 | ndst3 |  |  | 29027 | -0.927 | -0.1559 | No |
| 1994 | 445194 | zgc:101040 |  |  | 29029 | -0.927 | -0.1553 | No |
| 1995 | 436646\_2 | galm |  |  | 29033 | -0.927 | -0.1548 | No |
| 1996 | 792097 | amd1 |  |  | 29042 | -0.929 | -0.1544 | No |
| 1997 | 497183 | grhpra |  |  | 29048 | -0.931 | -0.1539 | No |
| 1998 | 407706 | st6galnac |  |  | 29063 | -0.935 | -0.1538 | No |
| 1999 | 799278 | si:ch211-168k14.2 |  |  | 29073 | -0.936 | -0.1534 | No |
| 2000 | 393778\_1 | prdx6 |  |  | 29110 | -0.944 | -0.1540 | No |
| 2001 | 559001\_1 | fasn |  |  | 29124 | -0.950 | -0.1538 | No |
| 2002 | 337489 | plcd1b |  |  | 29137 | -0.954 | -0.1535 | No |
| 2003 | 678558 | mtmr7a |  |  | 29143 | -0.956 | -0.1530 | No |
| 2004 | 550242 | nadka |  |  | 29154 | -0.960 | -0.1527 | No |
| 2005 | 445083\_1 | dhrs3a |  |  | 29161 | -0.962 | -0.1522 | No |
| 2006 | 503759 | acp2 |  |  | 29183 | -0.969 | -0.1522 | No |
| 2007 | 799528 | sqlea |  |  | 29200 | -0.974 | -0.1521 | No |
| 2008 | 394130 | acad8 |  |  | 29204 | -0.975 | -0.1515 | No |
| 2009 | 335751 | cox7a3 |  |  | 29214 | -0.977 | -0.1512 | No |
| 2010 | 450003 | gcdhb |  |  | 29282 | -0.995 | -0.1527 | No |
| 2011 | 565235 | aldh5a1 |  |  | 29304 | -1.001 | -0.1527 | No |
| 2012 | 445287\_1 | hpdb |  |  | 29361 | -1.018 | -0.1539 | No |
| 2013 | 561756\_1 | b4galt3 |  |  | 29366 | -1.020 | -0.1533 | No |
| 2014 | 557059 | zgc:152670 |  |  | 29371 | -1.021 | -0.1528 | No |
| 2015 | 791628 | sirt4 |  |  | 29404 | -1.032 | -0.1531 | No |
| 2016 | 334098 | hsd11b2 |  |  | 29418 | -1.038 | -0.1528 | No |
| 2017 | 553683 | lpcat2 |  |  | 29424 | -1.040 | -0.1523 | No |
| 2018 | 100331179 | acer2 |  |  | 29449 | -1.046 | -0.1524 | No |
| 2019 | 406736 | mgst3a |  |  | 29458 | -1.048 | -0.1519 | No |
| 2020 | 335485\_1 | plpp2b |  |  | 29460 | -1.049 | -0.1512 | No |
| 2021 | 406575 | lta4h |  |  | 29495 | -1.062 | -0.1516 | No |
| 2022 | 110437777 | LOC110437777 |  |  | 29497 | -1.063 | -0.1509 | No |
| 2023 | 393713 | fhit |  |  | 29500 | -1.063 | -0.1503 | No |
| 2024 | 555317\_1 | si:ch211-276a23.5 |  |  | 29554 | -1.079 | -0.1513 | No |
| 2025 | 569142 | entpd2b |  |  | 29566 | -1.083 | -0.1509 | No |
| 2026 | 100004331 | hal |  |  | 29573 | -1.085 | -0.1504 | No |
| 2027 | 568744 | gstm.3 |  |  | 29605 | -1.097 | -0.1506 | No |
| 2028 | 556522 | agmat |  |  | 29620 | -1.102 | -0.1504 | No |
| 2029 | 494054\_1 | tm7sf2 |  |  | 29622 | -1.102 | -0.1496 | No |
| 2030 | 393982 | alpl |  |  | 29631 | -1.104 | -0.1491 | No |
| 2031 | 558930\_1 | si:dkey-160o24.3 |  |  | 29648 | -1.113 | -0.1489 | No |
| 2032 | 324079 | adi1 |  |  | 29660 | -1.115 | -0.1485 | No |
| 2033 | 554477 | pfkfb3 |  |  | 29670 | -1.119 | -0.1480 | No |
| 2034 | 100007749 | gphnb |  |  | 29678 | -1.121 | -0.1475 | No |
| 2035 | 327507 | tgds |  |  | 29679 | -1.122 | -0.1467 | No |
| 2036 | 100005067\_1 | dguok |  |  | 29683 | -1.122 | -0.1460 | No |
| 2037 | 393668 | eno1b |  |  | 29713 | -1.133 | -0.1462 | No |
| 2038 | 777632\_1 | mccc1 |  |  | 29720 | -1.135 | -0.1456 | No |
| 2039 | 795256 | si:dkey-78a14.5 |  |  | 29751 | -1.149 | -0.1458 | No |
| 2040 | 553585 | echdc1 |  |  | 29752 | -1.149 | -0.1450 | No |
| 2041 | 335231 | fbp1a |  |  | 29764 | -1.152 | -0.1446 | No |
| 2042 | 406473\_1 | pck2 |  |  | 29767 | -1.153 | -0.1439 | No |
| 2043 | 393441\_1 | hacd3 |  |  | 29812 | -1.168 | -0.1446 | No |
| 2044 | 571850 | miox |  |  | 29822 | -1.173 | -0.1440 | No |
| 2045 | 334121 | hacd2 |  |  | 29838 | -1.181 | -0.1437 | No |
| 2046 | 445094 | pkmb |  |  | 29848 | -1.183 | -0.1432 | No |
| 2047 | 324134 | asmtl |  |  | 29875 | -1.193 | -0.1433 | No |
| 2048 | 100002015 | cds1 |  |  | 29893 | -1.200 | -0.1430 | No |
| 2049 | 569254 | gkup |  |  | 29895 | -1.201 | -0.1422 | No |
| 2050 | 393983 | pfkfb2a |  |  | 29908 | -1.206 | -0.1418 | No |
| 2051 | 541364 | zgc:113333 |  |  | 29912 | -1.206 | -0.1410 | No |
| 2052 | 406648 | ppt1 |  |  | 29919 | -1.210 | -0.1404 | No |
| 2053 | 560827 | prps1b |  |  | 29931 | -1.217 | -0.1399 | No |
| 2054 | 30733 | rrm2 |  |  | 29935 | -1.219 | -0.1392 | No |
| 2055 | 561117 | atp6v0a2b |  |  | 29956 | -1.226 | -0.1390 | No |
| 2056 | 334195 | mat1a |  |  | 29966 | -1.230 | -0.1385 | No |
| 2057 | 100006238\_1 | aldh9a1a.2 |  |  | 29971 | -1.231 | -0.1377 | No |
| 2058 | 777719\_1 | papss2a |  |  | 30038 | -1.262 | -0.1391 | No |
| 2059 | 571938 | pld3 |  |  | 30041 | -1.263 | -0.1383 | No |
| 2060 | 450017 | atp6v0e1 |  |  | 30042 | -1.263 | -0.1374 | No |
| 2061 | 325316 | pigp |  |  | 30058 | -1.267 | -0.1370 | No |
| 2062 | 101886068 | si:dkey-177p2.18 |  |  | 30079 | -1.276 | -0.1368 | No |
| 2063 | 394138 | asns |  |  | 30083 | -1.278 | -0.1360 | No |
| 2064 | 402831 | cyp27a7 |  |  | 30086 | -1.279 | -0.1352 | No |
| 2065 | 445326 | akr1a1a |  |  | 30087 | -1.279 | -0.1343 | No |
| 2066 | 393744\_2 | dctpp1 |  |  | 30099 | -1.285 | -0.1338 | No |
| 2067 | 796997\_1 | ndufs7 |  |  | 30117 | -1.298 | -0.1335 | No |
| 2068 | 415103\_1 | tph1b |  |  | 30140 | -1.309 | -0.1333 | No |
| 2069 | 767688 | zgc:154040 |  |  | 30161 | -1.321 | -0.1331 | No |
| 2070 | 553653\_1 | amacr |  |  | 30179 | -1.326 | -0.1327 | No |
| 2071 | 553239\_1 | lrata |  |  | 30182 | -1.327 | -0.1319 | No |
| 2072 | 558466 | uckl1b |  |  | 30217 | -1.346 | -0.1321 | No |
| 2073 | 103910413 | tymp |  |  | 30218 | -1.348 | -0.1311 | No |
| 2074 | 792692\_1 | aldoca |  |  | 30223 | -1.351 | -0.1303 | No |
| 2075 | 393315 | ccbl2 |  |  | 30227 | -1.353 | -0.1295 | No |
| 2076 | 548336 | ak5l |  |  | 30260 | -1.372 | -0.1296 | No |
| 2077 | 562840\_1 | rdh10a |  |  | 30303 | -1.391 | -0.1301 | No |
| 2078 | 569989 | ca5a |  |  | 30322 | -1.399 | -0.1297 | No |
| 2079 | 100004977\_2 | mthfd2 |  |  | 30332 | -1.405 | -0.1290 | No |
| 2080 | 100002020 | si:ch211-126g16.10 |  |  | 30333 | -1.405 | -0.1281 | No |
| 2081 | 559198\_1 | mtmr7b |  |  | 30356 | -1.421 | -0.1278 | No |
| 2082 | 561803 | gyg2 |  |  | 30363 | -1.423 | -0.1270 | No |
| 2083 | 402830\_1 | ehmt1b |  |  | 30364 | -1.423 | -0.1260 | No |
| 2084 | 406789\_2 | pank1a |  |  | 30373 | -1.430 | -0.1253 | No |
| 2085 | 541511 | ippk |  |  | 30388 | -1.441 | -0.1248 | No |
| 2086 | 573619\_1 | ndufab1a |  |  | 30417 | -1.458 | -0.1247 | No |
| 2087 | 100003675\_1 | p4ha1b |  |  | 30423 | -1.461 | -0.1239 | No |
| 2088 | 561475 | plcd4b |  |  | 30447 | -1.475 | -0.1236 | No |
| 2089 | 557632 | si:ch211-197l9.4 |  |  | 30456 | -1.479 | -0.1229 | No |
| 2090 | 799868 | st6galnac1.2 |  |  | 30458 | -1.480 | -0.1219 | No |
| 2091 | 553981\_1 | sptlc1 |  |  | 30460 | -1.483 | -0.1209 | No |
| 2092 | 100333665\_1 | gbe1a |  |  | 30463 | -1.484 | -0.1199 | No |
| 2093 | 557675 | c1galt1a |  |  | 30464 | -1.485 | -0.1189 | No |
| 2094 | 795106 | cyp27a1.2 |  |  | 30481 | -1.495 | -0.1184 | No |
| 2095 | 394181 | cox15 |  |  | 30485 | -1.497 | -0.1175 | No |
| 2096 | 568001\_1 | pfkmb |  |  | 30537 | -1.531 | -0.1181 | No |
| 2097 | 394009 | srm |  |  | 30543 | -1.536 | -0.1172 | No |
| 2098 | 100003383\_1 | pmvk |  |  | 30562 | -1.557 | -0.1167 | No |
| 2099 | 407663 | si:ch211-107o10.3 |  |  | 30565 | -1.558 | -0.1157 | No |
| 2100 | 406731 | ugt1ab |  |  | 30566 | -1.558 | -0.1146 | No |
| 2101 | 64607\_1 | alas2 |  |  | 30586 | -1.573 | -0.1142 | No |
| 2102 | 101884072 | mgat5b |  |  | 30596 | -1.586 | -0.1134 | No |
| 2103 | 368775\_1 | ivd |  |  | 30609 | -1.595 | -0.1127 | No |
| 2104 | 571356\_1 | pik3c2a |  |  | 30618 | -1.605 | -0.1118 | No |
| 2105 | 100007686 | LOC100007686 |  |  | 30621 | -1.607 | -0.1108 | No |
| 2106 | 406688 | got2a |  |  | 30634 | -1.617 | -0.1101 | No |
| 2107 | 561891\_1 | minpp1a |  |  | 30635 | -1.618 | -0.1090 | No |
| 2108 | 447914 | cox17 |  |  | 30691 | -1.664 | -0.1097 | No |
| 2109 | 393330\_1 | zgc:64002 |  |  | 30717 | -1.687 | -0.1093 | No |
| 2110 | 394188 | qars |  |  | 30725 | -1.703 | -0.1084 | No |
| 2111 | 399485 | cyp2p10 |  |  | 30726 | -1.705 | -0.1072 | No |
| 2112 | 406703 | gsta.1 |  |  | 30735 | -1.713 | -0.1063 | No |
| 2113 | 436725 | acp5b |  |  | 30758 | -1.726 | -0.1058 | No |
| 2114 | 100148818 | si:ch73-55i23.1 |  |  | 30770 | -1.736 | -0.1050 | No |
| 2115 | 566479\_1 | plch1 |  |  | 30774 | -1.739 | -0.1039 | No |
| 2116 | 393860\_1 | gcdha |  |  | 30857 | -1.817 | -0.1054 | No |
| 2117 | 393293 | hsd11b1la |  |  | 30858 | -1.817 | -0.1041 | No |
| 2118 | 368262 | dlst |  |  | 30863 | -1.822 | -0.1030 | No |
| 2119 | 794381 | si:dkey-78a14.4 |  |  | 30868 | -1.827 | -0.1019 | No |
| 2120 | 100330155 | cers4b |  |  | 30886 | -1.848 | -0.1012 | No |
| 2121 | 437016\_1 | tk2 |  |  | 30898 | -1.862 | -0.1002 | No |
| 2122 | 100006448 | ca6 |  |  | 30900 | -1.863 | -0.0990 | No |
| 2123 | 565246 | si:dkey-192p21.6 |  |  | 30902 | -1.868 | -0.0977 | No |
| 2124 | 566675 | suox |  |  | 30906 | -1.873 | -0.0965 | No |
| 2125 | 334217 | elovl7a |  |  | 30920 | -1.885 | -0.0956 | No |
| 2126 | 563138 | si:ch211-219a15.4 |  |  | 30925 | -1.888 | -0.0945 | No |
| 2127 | 322660 | upb1 |  |  | 30928 | -1.889 | -0.0932 | No |
| 2128 | 556535\_1 | setd1a |  |  | 30934 | -1.895 | -0.0921 | No |
| 2129 | 559054 | hmgcra |  |  | 30946 | -1.905 | -0.0911 | No |
| 2130 | 100005763\_1 | uck2a |  |  | 30953 | -1.908 | -0.0900 | No |
| 2131 | 100000684\_1 | neu3.5 |  |  | 30984 | -1.951 | -0.0897 | No |
| 2132 | 100329531\_1 | hmox2b |  |  | 31023 | -1.989 | -0.0896 | No |
| 2133 | 797309 | cyp2j20 |  |  | 31035 | -2.003 | -0.0885 | No |
| 2134 | 406339\_1 | hk2 |  |  | 31039 | -2.013 | -0.0873 | No |
| 2135 | 431773 | enoph1 |  |  | 31048 | -2.026 | -0.0861 | No |
| 2136 | 393427 | zgc:63568 |  |  | 31073 | -2.050 | -0.0855 | No |
| 2137 | 403070 | rgn |  |  | 31121 | -2.114 | -0.0856 | No |
| 2138 | 569363 | tpo |  |  | 31142 | -2.138 | -0.0848 | No |
| 2139 | 407673 | rpia |  |  | 31144 | -2.139 | -0.0834 | No |
| 2140 | 100002875\_1 | hmox2a |  |  | 31190 | -2.213 | -0.0833 | No |
| 2141 | 553239 | lrata |  |  | 31194 | -2.222 | -0.0819 | No |
| 2142 | 359840\_1 | atp6v1b2 |  |  | 31211 | -2.252 | -0.0809 | No |
| 2143 | 553575\_1 | gsr |  |  | 31216 | -2.255 | -0.0794 | No |
| 2144 | 553523 | trit1 |  |  | 31217 | -2.257 | -0.0779 | No |
| 2145 | 560753 | tpi1b |  |  | 31226 | -2.286 | -0.0766 | No |
| 2146 | 100093713 | zgc:165519 |  |  | 31232 | -2.293 | -0.0751 | No |
| 2147 | 324510 | gcnt4a |  |  | 31244 | -2.315 | -0.0739 | No |
| 2148 | 541329\_1 | gcsha |  |  | 31249 | -2.328 | -0.0724 | No |
| 2149 | 321892 | ckmt1 |  |  | 31252 | -2.335 | -0.0709 | No |
| 2150 | 792824\_1 | pgp |  |  | 31276 | -2.388 | -0.0700 | No |
| 2151 | 767759 | zgc:153704 |  |  | 31290 | -2.416 | -0.0688 | No |
| 2152 | 100005587\_1 | aldh9a1a.1 |  |  | 31308 | -2.447 | -0.0676 | No |
| 2153 | 568500 | galnt8a.2 |  |  | 31311 | -2.451 | -0.0660 | No |
| 2154 | 570913\_1 | ugt5g1 |  |  | 31325 | -2.473 | -0.0647 | No |
| 2155 | 561416 | pfkpb |  |  | 31341 | -2.509 | -0.0635 | No |
| 2156 | 550524\_1 | cbsa |  |  | 31356 | -2.544 | -0.0622 | No |
| 2157 | 368717\_1 | itpka |  |  | 31364 | -2.556 | -0.0607 | No |
| 2158 | 100536795 | wu:fb14d09 |  |  | 31372 | -2.574 | -0.0591 | No |
| 2159 | 393793\_1 | uqcrc1 |  |  | 31447 | -2.765 | -0.0597 | No |
| 2160 | 100526683\_1 | aldh1l1 |  |  | 31451 | -2.776 | -0.0578 | No |
| 2161 | 406370\_1 | pla2g6 |  |  | 31478 | -2.856 | -0.0567 | No |
| 2162 | 322670\_1 | aco2 |  |  | 31502 | -2.927 | -0.0555 | No |
| 2163 | 541429\_1 | aoc2 |  |  | 31543 | -3.032 | -0.0547 | No |
| 2164 | 100415796 | ugt5b5 |  |  | 31553 | -3.072 | -0.0529 | No |
| 2165 | 337236\_1 | synj1 |  |  | 31555 | -3.076 | -0.0508 | No |
| 2166 | 777719 | papss2a |  |  | 31559 | -3.081 | -0.0488 | No |
| 2167 | 100003615\_1 | prdm2a |  |  | 31589 | -3.227 | -0.0475 | No |
| 2168 | 569769 | pomt1 |  |  | 31609 | -3.292 | -0.0459 | No |
| 2169 | 327601\_1 | alg8 |  |  | 31637 | -3.401 | -0.0444 | No |
| 2170 | 723999 | zgc:136333 |  |  | 31649 | -3.456 | -0.0424 | No |
| 2171 | 561471 | pigl |  |  | 31652 | -3.472 | -0.0400 | No |
| 2172 | 394103\_1 | sardh |  |  | 31656 | -3.486 | -0.0377 | No |
| 2173 | 568001\_2 | pfkmb |  |  | 31666 | -3.543 | -0.0356 | No |
| 2174 | 791167 | zgc:158222 |  |  | 31768 | -4.275 | -0.0360 | No |
| 2175 | 447942\_1 | cox6a2 |  |  | 31777 | -4.401 | -0.0332 | No |
| 2176 | 492517\_1 | zgc:103586 |  |  | 31787 | -4.517 | -0.0304 | No |
| 2177 | 445250 | neu3.2 |  |  | 31793 | -4.548 | -0.0274 | No |
| 2178 | 447815\_1 | rimkla |  |  | 31806 | -4.678 | -0.0246 | No |
| 2179 | 445250\_1 | neu3.2 |  |  | 31824 | -4.790 | -0.0218 | No |
| 2180 | 724010 | dgkaa |  |  | 31827 | -4.806 | -0.0185 | No |
| 2181 | 406449 | ptges3a |  |  | 31841 | -4.882 | -0.0156 | No |
| 2182 | 394230\_1 | st3gal5 |  |  | 31847 | -4.897 | -0.0124 | No |
| 2183 | 564754\_1 | nat8l |  |  | 31851 | -4.905 | -0.0091 | No |
| 2184 | 550579 | idh3g |  |  | 31862 | -4.927 | -0.0060 | No |
| 2185 | 571201 | si:ch73-97h19.2 |  |  | 31873 | -4.947 | -0.0029 | No |
| 2186 | 447801 | aldh8a1 |  |  | 31875 | -4.952 | 0.0005 | No |
Table: GSEA details [plain text format]

  

Fig 2: METABOLIC\_PATHWAYS(DRE01100)      
 Blue-Pink O' Gram in the Space of the Analyzed GeneSet

  

Fig 3: METABOLIC\_PATHWAYS(DRE01100): Random ES distribution      
 Gene set null distribution of ES for **METABOLIC\_PATHWAYS(DRE01100)**
